# Functional connectomics reveals general wiring rule in mouse visual cortex

**DOI:** 10.1101/2023.03.13.531369

**Published:** 2023-03-14

**Authors:** Zhuokun Ding, Paul G. Fahey, Stelios Papadopoulos, Eric Wang, Brendan Celii, Christos Papadopoulos, Alexander B. Kunin, Andersen Chang, Jiakun Fu, Zhiwei Ding, Saumil Patel, Kayla Ponder, J. Alexander Bae, Agnes L. Bodor, Derrick Brittain, JoAnn Buchanan, Daniel J. Bumbarger, Manuel A. Castro, Erick Cobos, Sven Dorkenwald, Leila Elabbady, Akhilesh Halageri, Zhen Jia, Chris Jordan, Dan Kapner, Nico Kemnitz, Sam Kinn, Kisuk Lee, Kai Li, Ran Lu, Thomas Macrina, Gayathri Mahalingam, Eric Mitchell, Shanka Subhra Mondal, Shang Mu, Barak Nehoran, Sergiy Popovych, Casey M. Schneider-Mizell, William Silversmith, Marc Takeno, Russel Torres, Nicholas L. Turner, William Wong, Jingpeng Wu, Wenjing Yin, Szi-chieh Yu, Emmanouil Froudarakis, Fabian Sinz, H. Sebastian Seung, Forrest Collman, Nuno Maçarico da Costa, R. Clay Reid, Edgar Y. Walker, Xaq Pitkow, Jacob Reimer, Andreas S. Tolias

**Affiliations:** 1 Center for Neuroscience and Artificial Intelligence, Baylor College of Medicine, Houston, USA; 2 Department of Neuroscience, Baylor College of Medicine, Houston, USA; 3 Allen Institute for Brain Science, Seattle, USA; 4 Princeton Neuroscience Institute, Princeton University, Princeton, USA; 5 Electrical and Computer Engineering Department, Princeton University, Princeton, USA; 6 Computer Science Department, Princeton University, Princeton, USA; 7 Brain & Cognitive Sciences Department, Massachusetts Institute of Technology, Cambridge, USA; 8 Department of Mathematics, Creighton University, Omaha, USA; 9 Department of Electrical and Computer Engineering, Rice University, Houston, USA; 10 Department of Physiology and Biophysics, University of Washington, Seattle, USA; 11 UW Computational Neuroscience Center, University of Washington, Seattle, USA; 12 Institute of Molecular Biology and Biotechnology, Foundation for Research and Technology Hellas, Heraklion, Greece; 13 Institute for Bioinformatics and Medical Informatics, University Tübingen, Tübingen, Germany; 14 Institute for Computer Science and Campus Institute Data Science, University Göttingen, Göttingen, Germany

## Abstract

To understand how the neocortex underlies our ability to perceive, think, and act, it is important to study the relationship between circuit connectivity and function. Previous research has shown that excitatory neurons in layer 2/3 of the primary visual cortex of mice with similar response properties are more likely to form connections. However, technical challenges of combining synaptic connectivity and functional measurements have limited these studies to few, highly local connections. Utilizing the millimeter scale and nanometer resolution of the MICrONS dataset, we studied the connectivity-function relationship in excitatory neurons of the mouse visual cortex across interlaminar and interarea projections, assessing connection selectivity at the coarse axon trajectory and fine synaptic formation levels. A digital twin model of this mouse, that accurately predicted responses to arbitrary video stimuli, enabled a comprehensive characterization of the function of neurons. We found that neurons with highly correlated responses to natural videos tended to be connected with each other, not only within the same cortical area but also across multiple layers and visual areas, including feedforward and feedback connections, whereas we did not find that orientation preference predicted connectivity. The digital twin model separated each neuron’s tuning into a feature component (what the neuron responds to) and a spatial component (where the neuron’s receptive field is located). We show that the feature, but not the spatial component, predicted which neurons were connected at the fine synaptic scale. Together, our results demonstrate the “like-to-like” connectivity rule generalizes to multiple connection types, and the rich MICrONS dataset is suitable to further refine a mechanistic understanding of circuit structure and function.

## Introduction

In the late 1800’s, Santiago Ramón y Cajal — while poring over the structure of Golgi-stained neurons using only light microscopy — imagined the Neuron Doctrine, the idea that individual neurons are the fundamental units of the nervous system (Ramón y Cajal, 1911). From that moment, understanding how cortical computation emerges from those individual neurons was linked to understanding the relationship between their connectivity and function. A variety of influential proposals about this relationship have been advanced in the past century. For example, Donald Hebb’s “cell assembly” hypothesis (Hebb, 1949) — colloquially stated as “neurons that fire together, wire together” — predicted that interconnected neuronal subnetworks “reverberate” to stabilize functionally relevant activity patterns. In the visual system, Hubel and Wiesel proposed that the hierarchical organization of connected neurons might build feature selectivity; for example the orientation selectivity of simple cells might be derived from convergent inputs from neurons in the lateral geniculate nucleus whose receptive fields are arranged along a straight line in the visual field. (Hubel and Wiesel, 1962). Although significant insight can be gleaned from functional or structural analysis alone, thoroughly testing these predictions requires information about both neural activity and connectivity in the same set of neurons. In the mammalian visual cortex, evidence for several varieties of “like-to-like” connectivity (i.e. increased connectivity for cells with similar response preferences) has been found via spine imaging (Iacaruso et al., 2017), combined *in vivo* imaging and *in vitro* multipatching (Ko et al., 2011; Cossell et al., 2015), combined *in vivo* imaging and rabies monosynaptic retrograde tracing (Wertz et al., 2015), and combined *in vivo* imaging with electron microscopy (EM) reconstruction (Bock et al., 2011; Lee et al., 2016; Scholl et al., 2021). The results of these early functional connectomics studies are consistent with an organization reminiscent of Hebbian cell assemblies, where interconnected pyramidal subnetworks with similar feature preferences amplify sensory input, perhaps to sharpen tuning or overcome a strong inhibitory tone (Lien and Scanziani, 2013; Reinhold et al., 2015; Lee et al., 2016).

However, these important early studies that yielded the first glimpses of functional-structural rules required monumental effort just to examine small populations of neurons restricted to small volumes of primary visual cortex (V1), and typically limited to cortical Layers 2 and 3 (L2/3). This is due in part to the challenge of collecting functional connectomics data and especially the challenge of identifying synaptic connections between neurons across distances larger than a few hundred microns. Therefore, many questions remain unanswered about how these rules generalize across areas and layers, including connections within and between different layers and areas (including feedforward and feedback), and how they relate to local and hierarchical mechanisms of sensory processing.

Enormous strides have been made over the past decade in our ability to record activity over large populations of neurons distributed across multiple regions of the brain (Sofroniew et al., 2016; Pachitariu et al., 2017; Allen et al., 2019; Stringer et al., 2019; Demas et al., 2021; Steinmetz et al., 2021; Jun et al., 2017). Recent technological innovations in serial electron microscopy (Yin et al., 2020; Phelps et al., 2021) and automatic dense reconstruction using deep learning (Turner et al., 2020; Dorkenwald et al., 2022b; Mitchell et al., 2019; Lu et al., 2021; Wu et al., 2021; Dorkenwald et al., 2022a; Lee et al., 2017), when combined with mesoscopic two-photon imaging (Sofroniew et al., 2016), have converged to enable collection of the MICrONS dataset, the largest functionally-imaged and densely-reconstructed calcium imaging/EM dataset to date (MICrONS Consortium et al., 2021).

Here, dense reconstruction means that every membrane compartment in the volume is segmented into an axon, dendrite, glia, etc. — in contrast with previous studies that have sparsely reconstructed connections from or to a limited number of functionally-characterized target cells (Lee et al., 2016; Bock et al., 2011). As a result, we were able to gather information from a higher density of reconstructed “bystanders”, nearby neurons that could have formed connections, yet didn’t. This allows for a multi-tiered analysis at different spatial scales, with a coarse level corresponding to the axonal trajectory past the dendrites of some neurons but not others, and a fine level at which it may form synapses only with a subset of those candidate neurons. This multi-tiered analysis enables a more comprehensive understanding of the mechanisms by which neurites select their synaptic partners, including sharing certain functional properties, and thus help shed light on the complex interplay between structure and function in the nervous system.

Our functional analysis utilized a digital twin model of the recorded neurons (Wang et al., 2023), which was able to accurately predict how neurons responded to dynamic natural stimuli. With this model, we were able to conduct a thorough characterization of neuronal function. Our findings revealed that neurons with highly correlated responses to natural videos tended to be connected with each other, not only within the same cortical areas but also across multiple layers and visual areas, including feedforward and feedback connections. Interestingly, we did not find evidence that connected neurons share similar orientation tuning. The digital twin model allowed us to separate each neuronal tuning into two components: a feature component (what the neuron responded to), and a spatial component (the location of the neuron’s receptive field). Further analysis showed that the feature component, rather than the spatial component, predicted fine-scale synaptic connections between neurons. Lastly, we showed that signal correlation and feature tuning each uniquely contribute to predicting synaptic level connectivity. Our results provide support for the “like-to-like” connectivity rule across different types of connections (local, interarea, interlaminar, etc) and highlight the potential of the MICrONS dataset in enhancing our understanding of circuit structure and function.

## Results

### MICrONS functional connectomic dataset.

**Figure 1.**
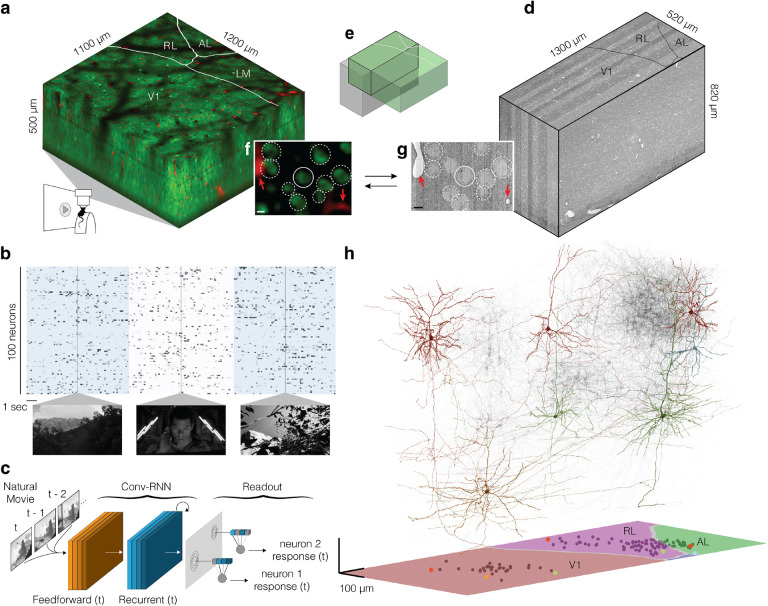
Overview of MICrONS Dataset. **a**, Depiction of functionally characterized volume in right visual cortex (green: GCaMP6s, red: vascular label), using two-photon (2P) mesoscopic imaging of awake, head-mounted mouse with movie visual stimuli presented to the left eye. Visual areas: primary visual cortex (V1), anterolateral (AL), lateromedial (LM) and rostrolateral (RL). **b**, Representation of deconvolved calcium traces of 100 imaged neurons. Alternating blue/white column overlay represents the duration of serial video trials, with sample frames of natural videos depicted below. Parametric stimuli (not pictured) were also shown for a shorter duration and included in the model training set. **c**, Schematic representing digital twin deep recurrent architecture. During training, subsequent movie frames (left) are inputted into a shared convolutional deep recurrent core (orange, blue layers) resulting in extracted representation of local spatiotemporal stimulus features. Each neuron learns the location (*spatial component* in the visual field (gray layer) to read out feature activations (shaded blue vectors), and the dot product with the neuron-specific learned feature weights (shaded lines, *feature component*) results in the predicted mean neural activation for that time point. **d**, Depiction of the structurally characterized and densely reconstructed EM subvolume 65. **e**, Overlap of the functional 2P (green) and structural EM (gray) volumes, from which somas were recruited. **f, g**, Demonstration of corresponding structural features in 2P (**f**) and EM (**g**) volumes, including soma constellations (dotted white circles) and unique local vasculature (red arrowheads), used to build confidence in the manually assigned 2P-EM cell match (solid white circle). Scale bars = 5μm. **h**, Depiction of 122 manually proofread mesh reconstructions (gray), including representative samples from Layer 2/3 (red), Layer 4 (blue), Layer 5 (green), and Layer 6 (yellow). Bottom panel: presynaptic soma locations relative to visual area boundaries.

Data were collected and processed as described in the MICrONS data release publication (MICrONS Consortium et al. 2021, Fig. 1). Briefly, a single mouse expressing GCaMP6s in excitatory neurons underwent fourteen two-photon scans of a 1200 × 1100 × 500μm^3^ volume (anteroposterior × mediolateral × radial depth) spanning layers 2 through 6 at the conjunction of lateral primary visual cortex and anterolateral (AL), lateromedial (LM) and rostrolateral (RL) higher visual areas (HVAs, Fig. 1a). Neuronal responses in the awake, behaving animal from 115,372 functional units representing an estimated 75,909 unique excitatory neurons were collected in response to visual stimuli composed of natural and rendered movies and parametric dynamic stimuli (Fig. 1b). A state-of-the-art deep recurrent neural network was trained to predict neural responses to arbitrary stimuli (Wang et al., 2023), and used to characterize the *in silico* functional properties of imaged neurons (Fig. 1c).

After functional imaging, the tissue was fixed and a block encompassing the functionally characterized volume was dissected for osmium staining, resin embedding, and ultra-thin sectioning for electron microscopy (Yin et al., 2020) at 4 × 4 × 40nm^3^ resolution (Fig. 1d). The EM images were aligned (Mitchell et al., 2019) and automatically segmented using 3D convolutional networks into “atomic” supervoxels, which were agglomerated to create objects (e.g. neurons) with corresponding 3D meshes (Lee et al., 2017; Dorkenwald et al., 2022b; Lu et al., 2021; Wu et al., 2021; Dorkenwald et al., 2022a). Synaptic clefts were predicted from the EM data and assigned to presynaptic and postsynaptic partners by 3D convolutional networks similar to that used for segmentation (Dorkenwald et al., 2022b; Turner et al., 2020; Wu et al., 2021). The densely reconstructed EM volume spanned roughly 870 × 1300 × 820μm^3^ (anteroposterior × mediolateral × radial depth) after alignment with the functional volume. The analysis presented here is restricted to the contents of subvolume 65 (roughly 65% of the total EM volume along the anteroposterior axis, see MICrONS Consortium et al. 2021 for details), which contained an approximately 560×1100×500μm^3^ volume (*in vivo* dimensions) of overlapping two-photon and EM that has been both densely functionally and structurally characterized. Of 82,247 automatically extracted neuronal nuclei in subvolume 65, 45,334 were both classified as excitatory and located within the intersection of the EM reconstructed volume and functional volume.

The two-photon and EM volumes were approximately aligned (Fig. 1e), and 8905 excitatory neurons were manually matched between the two volumes (Fig. 1f, g; MICrONS Consortium et al. 2021). Visually responsive and well characterized neurons in retinotopically matched areas in V1 and HVA were chosen for manual morphological proofreading focused on extending axonal branches projecting across the boundary of primary visual cortex and removing inappropriate merges (MICrONS Consortium et al., 2021). Postsy napticpartners of the proofread neurons were automatically cleaned of inappropriate merge errors (Celii et al., 2023). In total, this resulted in a connectivity graph consisting of 122 presynaptic neurons and 1975 postsynaptic partners with function characterized in the digital twin (Fig. 1h).

### Multi-tiered anatomical controls.

**Figure 2.**
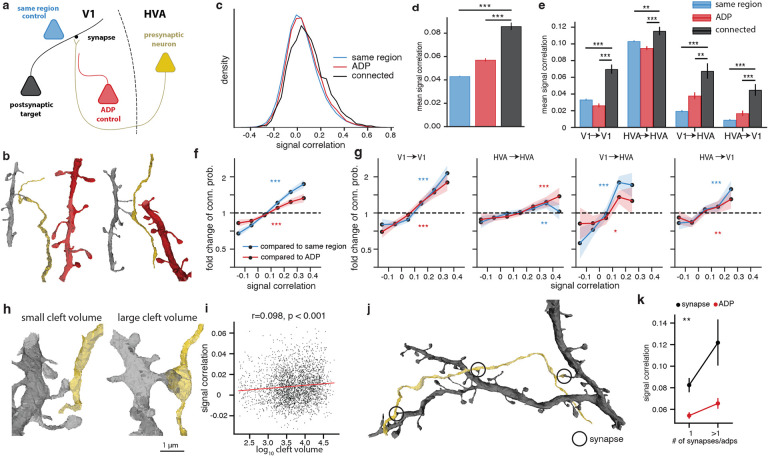
Neurons with higher signal correlation are more likely to form synapses. **a**, Schematic illustrating inclusion criteria for anatomical controls of increasing specificity. For each proofread presynaptic neuron (yellow), controls for its true postsynaptic partners (black) are drawn from neurons located in the same cortical region (blue), or from neurons with at least one Axonal-Dendritic Proximity (“ADP”) on the presynaptic axonal arbor (red). **b**, Representative meshes demonstrating a true presynaptic (yellow) to postsynaptic (black) pair and corresponding ADP control (red). **c**, Density histogram of pairwise signal correlation between observed presynaptic - postsynaptic partners (black) is right-shifted with respect to the control groups described in **a**. **d**, Mean signal correlation is different (mean ± sem, two-sample t-test) between observed presynaptic - postsynaptic partners (black), same region control (blue), and ADP control (red). **e**, Mean signal correlation of connected neurons is increased with respect to controls as in **d**, for within-area (V1 and HVA), feedforward, and feedback connectivity. **f**, Fold change of connection probability given signal correlation of true synaptic connectivity relative to controls is decreased for low signal correlations and increased for high signal correlations. (Error bar = ± 1 STD by bootstrap, p values by Cochran-Armitage two-sided test for trend). **g**, Fold change of connection probability is decreased for low signal correlations and increased for high signal correlations as in **d** for within-area (V1 and HVA), feedforward, and feedback connectivity. **h**, Representative meshes demonstrating synapses with low cleft volume (896 voxels, left) and high cleft volume (41716 voxels, right). **i**, Synapse size (*log*_10_ cleft volume in voxels) positively correlated with signal correlation. **j**, Representative meshes demonstrating a multisynaptic presynaptic (yellow) to postsynaptic (black) pair. **k**, Signal correlations between connected neurons with more than 1 observed synapse are higher than connected neurons with 1 observed synapse, after controlling for synapse opportunity by ADP (p values by two-way ANOVA). (For all panels,* = p-value < 0.05, = p-value < 0.01, * * * = p-value < 0.001, multiple comparison correction by BH procedure)

Connectivity between neurons is affected by numerous mechanisms, ranging from developmental processes that organize broad patterns of functional tuning and neurite growth, to mechanisms of synaptic formation and plasticity that modulate the strength of individual connections between neurons. Thus, it is important to differentiate connectivity patterns that can be explained by the spatial locations of cell bodies, axons and dendrites, from those which require additional specificity. Because the dense reconstruction provides information not only on the detailed morphology of the axonal arbor of presynaptic neurons and dendritic arbor of postsynaptic neurons, but also on the dendritic arbors of “bystander” neurons with no observed connection, it allows for the creation of specific and multi-tiered controls for testing hypotheses on the relationship between function and connectivity. In this study, we compare the population of connected neurons against two groups of control neurons with progressively tighter inclusion criteria (Fig. 2a, b). The first is the “same region” control, which includes all reliably visually responsive excitatory neurons (*CC*_*max*_ > 0.6) that are accurately predicted by the digital twin (*CC*_*abs*_ > 0.35), have been matched to the EM volume, and are located in the same cortical region (V1 vs HVA) as the postsynaptic target, but were not observed to form a synapse with the presynaptic neuron. The second control group is the Axonal-Dendritic Proximity (ADP) control, which further restricts the neurons in the same region control to those with the opportunity to synapse, as defined by a dendrite passing within 5μm of the presynaptic neuron axonal skeleton and also within 10μm of at least one synapse in the presynaptic axonal arbor (3D euclidean distance). The functional properties of the two control groups and the observed synaptic partners can be compared against each other to better interpret the different contributions to synaptic partner selectivity. The difference between the ADP control and same region control represents a coarse level selectivity related to the axon’s trajectory to some areas and layers of visual cortex but not others. For example, if the targeted cortical area is organized with respect to functional properties such as receptive field location (i.e. retinotopy) or preferred orientation (Fahey et al., 2019), then the functional similarity of the synaptic partners of the axon could be due to where it projects within that area, even if it synapses randomly within that location. On the other hand, the difference between the connected neurons and the ADP group represents a fine level selectivity related to which ADPs are converted into synapses.

### Signal correlation is selected at axon trajectory and synaptic levels.

For pairs of connected neurons and controls included under the constraints described above, the digital twin was used to calculate the *in silico* signal correlation in response to a large battery of novel natural movies (250 clips, 10 seconds per clip). By using the predicted mean response from the digital twin, which would require many repeats to obtain with *in vivo* measurements, we are able to explore a much larger stimulus space with *in silico* experiments than would be possible with *in vivo* measurements (Wang et al., 2023). We found that the distribution of *in silico* signal correlations for observed synapses had a small but significant positive shift relative to both controls (Fig. 2c, d; p-value < 0.001 for both comparisons, two-sided two-sample t-test corrected for multiple comparisons with Benjamini-Hochberg (BH) procedure). This pattern was also independently observed when subsets of neuron pairs were grouped into local V1, local HVA, feedforward (V1 *→* HVA) and feedback (HVA *→* V1) connections (Fig. 2e). Notably, when testing local and interarea selectivity separately, it may potentially be confounded by differences in spatial distribution between local and interarea arbors, for example due to incomplete reconstruction following proofreading emphasis on projecting interarea axons. To measure how signal correlation affects connection probability compared to either same region or ADP control, we quantified the fold changes in connection probability as a function of signal correlation. We observed that connection probability is higher for neurons with larger signal correlations (Fig. 2f, p-value <0.001 for both comparisons, Cochran-Armitage two-sided tests for trend). This increased connectivity was stronger in the same region control but remained positive in the more restrictive ADP control. For a small group of highly correlated neurons (>0.3 signal correlation, 5.2% of neurons), connection probability reached as high as 1.8 fold increase relative to the same region control and 1.5 fold increase relative to the ADP control. This relationship was observed in both local projections within V1 and HVA, feedforward, and feedback projections (Fig. 2g).

### Functional similarity predicts volume and number of synapses.

Previous studies have found that presynaptic-postsynaptic pairs with greater functional similarity have greater synapse strength (Cossell et al., 2015) and larger postsynaptic density (PSD) area (Lee et al., 2016). In the MICrONS dataset, synapses were automatically segmented with cleft volume measurements, which is related to spine head volume, PSD area, and synaptic strength (Arellano et al., 2007; Holler et al., 2021; Dorkenwald et al., 2022b). We found that signal correlation positively correlates with cleft volume (Fig. 2h, i; pearson r = 0.098, p < 0.001). We also found that presynaptic-postsynaptic pairs with multiple synapses had higher signal correlation (Fig. 2j, k) when compared to monosynaptic pairs.

### Factorized *in silico* functional representation.

Due to the architecture of the digital twin (Fig.1c, Wang et al. 2023), each modeled neuron’s predicted response is determined by two factors: readout **location** in visual space—a pair of azimuth/altitude coordinates; and readout **feature weights**—the relative contribution of the core’s learned nonlinear output features in predicting the target neuron’s activity. For each neuron, the combination of this receptive field location and feature weights together encode everything the model has learned about that neuron’s functional properties, and enable the model’s predictive capacity for that neuron. This factorized *in silico* representation allowed us to examine the extent to which these two elements independently contribute to the relationship between signal correlation and connectivity seen in Fig. 2.

### Postsynaptic feature tuning is selected at the synaptic level.

**Figure 3.**
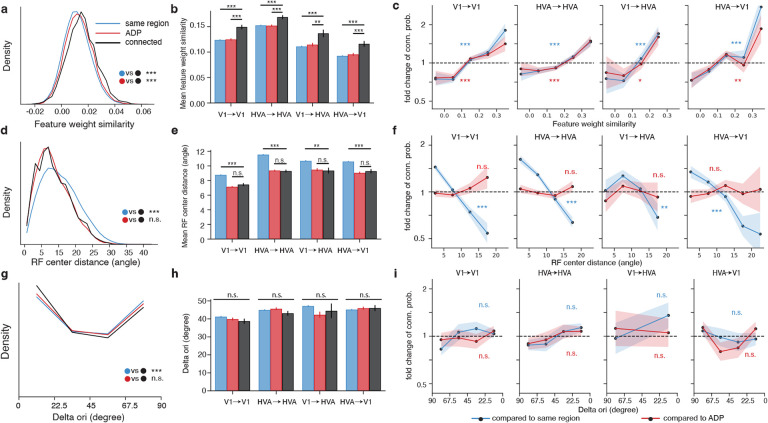
Feature weight similarity predicts synaptic selectivity better than receptive field center distance and difference in preferred orientation. **a, d, g**, Density histograms of pairwise model feature weight similarity (**a**), RF center distance (**d**), and difference in preferred orientation (Delta ori, **g**) for observed presynaptic-postsynaptic pairs (black) and controls. Mean signal correlation, mean RF center distance and mean difference in preferred orientation are compared across the connected neuron pairs and two controls (two sample t-test). **b, e, h**, Mean feature weight similarity (**b**), mean RF center distance (**e**), and mean difference in preferred orientation (**h**) for connected and control populations (two-sample t-test). **c, f, i**, Fold change in connection probability conditioned on feature weight similarity (**c**), receptive field center pairwise distance (**f**), and difference in preferred orientation (**i**). (Cochran-Armitage two-sided tests for trend) Error bars are bootstrapped STD. (For all panels, * = p-value < 0.05, ** = p-value < 0.01, * * * = p-value < 0.001, p-values are corrected for multiple comparison using BH procedure)

As seen with signal correlation above (Fig. 2c), the mean cosine similarity between the presynaptic and postsynaptic feature weights of the connected population is larger than both control populations (Fig. 3a, p-value <0.001, two-sample t-test). The local V1, local HVA, feedforward, and feedback projection breakout analyses further demonstrate selectivity at the synaptic level with respect to the model feature weight similarity (Fig. 3b, c). Higher feature weight similarity is also associated with larger synapse volume and multisynapse connectivity (Supp. Fig. 1a, b).

### Postsynaptic receptive field location is selected at the axon trajectory level.

Receptive field location similarity was measured as the visual angle difference between the model receptive field centers, with lesser center distance corresponding to greater location similarity. In contrast to signal correlation and feature weight similarity, receptive field location similarity is selected at the axon trajectory level, as evidenced by the leftward shift in receptive field location distance between connected neurons and same region control (black vs blue, Fig. 3d). This pattern is consistent with the decrease in receptive field location distance between same region control and connected neurons in the projection breakout (black vs blue, Fig. 3e) and with the decreasing trend in connection probability (blue, Fig. 3f). However, there is no statistically significant difference between the connected population and ADP control across all three analyses (black vs red, Fig. 3d, e, f), suggesting that there is not an additional synaptic selectivity on the basis of receptive field location beyond axonal targeting of retinotopically matched regions. Receptive field center distance also does not correlate with synapse volume (Supp. Fig. 1c), nor with multisynapse connectivity (Supp. Fig. 1d).

### Postsynaptic orientation tuning is selected at the axon trajectory level.

Previous work has found like-to-like connectivity with respect to similarity in orientation preference (Supp. Fig. 2a, b; Ko et al. 2011; Lee et al. 2016). Similar to the *in silico* signal correlation computed above, we extracted neuronal orientation tuning from the responses to *in silico* presentations of noise-based stimuli with coherent orientation and direction. Only orientation-tuned neurons are included in the analysis (global OSI > 0.25) which were shown to have similar *in silico* and *in vivo* orientation tuning properties in a separate set of experiments (Supp. Fig. 4). Overall orientation tuning of the volume revealed a cardinal bias (Kondo and Ohki, 2016; Salinas et al., 2017; Kreile et al., 2011), resulting in a U-shaped distribution in the difference in preferred orientation between presynaptic and postsynaptic neurons (Fig. 3g). While we did find a leftward shift of the overall connected distribution relative to the same region control (Fig. 3g, p-value = 0.003), we did not observe synapse level selectivity when comparing *in silico* orientation tuning in connected pairs against ADP controls, either at the overall level (Fig. 3g) or in the projection breakout analysis (Fig. 3h, i). Thus, for the portion of V1 captured in the connectivity graph used for these analyses, like-to-like connectivity with respect to *in silico* orientation tuning was only detected at the axon trajectory level, and not at the synapse level. However, in order to recruit an unbiased presynaptic population, candidates for proofreading were not chosen based on orientation tuning, and consequently only 87/122 (71%) of the presynaptic neurons were significantly tuned for orientation. To control for the decrease in sample size, we re-tested the relationship between connectivity and signal correlation (Fig. 2c, e, g), and between connectivity and feature weight similarity (Fig. 3a – c) with only the subsampled population with statistically significant orientation tuning (Supp. Fig. 3). We found that both signal correlation and feature weight relationships remained similar overall, suggesting the subsampling alone cannot account for the lack of relationship between similarity in orientation preference and connectivity.

### Like-to-like rule generalizes across joint layer and area membership of cells.

**Figure 4.**
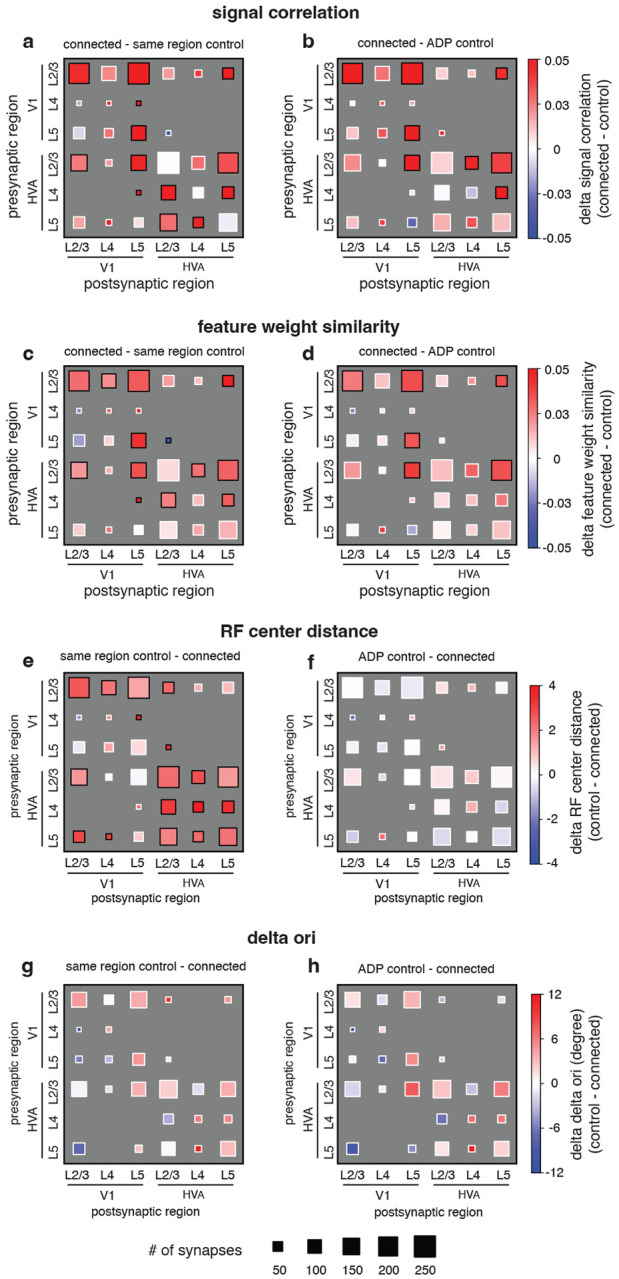
Like-to-like effects are widespread but vary across joint area / layer membership and tuning similarity metric. **a-h**, Hinton plot of functional similarity between presynaptic and postsynaptic neurons, broken down by area and layer membership, and relative to either same region control (**a, c, e, g**) or ADP control (**b, d, f, h**). (black border = significant at p-value < 0.05, white border = p-value > 0.05, by 2-sample t-test after BH correction for multiple comparisons).

To get a more detailed understanding of the organization of connections across layers and areas, for each functional similarity metric (signal correlation, feature weight similarity, receptive field center distance, and difference in preferred orientation), we also tested the relationship with connectivity across the joint distribution of two area groups (primary visual cortex, V1; higher visual areas AL and RL, HVA) and three layer groups (L2/3, L4, and L5, Fig. 4). For signal correlation (Fig. 4a, b) and feature weight similarity (Fig. 4c, d), like-to-like effects (red squares) were widespread across many area and layer combinations, relative to both same region and ADP controls. However, pertinent negatives include HVA L2/3 *→* HVA L2/3, for which we failed to reject the null hypothesis of no difference from ADP control, despite that this group had 296 synapses between 46 unique presynaptic and 207 unique postsynaptic neurons, suggesting that in at least some subgroups the like-to-like is either greatly diminished, highly variable, or perhaps entirely absent. In the case of RF center distance, while like-to-like effects (red squares) were widespread when compared to the same region control across many groups and layers, none were significant in the ADP comparison, suggesting that RF distance selectivity at the axon trajectory but not synaptic level is also consistent across tested excitatory cell types (Fig. 4e, f). Lastly, in the case of difference in preferred orientation, we were not able to detect significant differences between connected and control neuron pairs (Fig. 4g, h). When we examine orientation tuning for V1 L2/3 *→* V1 L2/3 connections specifically, we observed a similar trend of connection probability compared to previous literature (Supp. Fig. 2a, b), however the trend was not significant (p = 0.090 vs region control, p = 0.750 vs ADP control, Cochran-Armitage two-sided test for trend). Because the data are constrained to only presynaptic and postsynaptic pairs with significant orientation tuning, it is possible that the tuning and selectivity of a few individual presynaptic neurons may have an outsized influence on single categories. However, the V1 L2/3 *→* V1 L2/3 group in our data has a greater number of connections when compared to previous studies (Ko et al. 2011: 25 connections; Lee et al. 2016: 29 connections; this study: 126 connections), and only slightly fewer unique presynaptic neurons (Lee et al. 2016: 15 presyn., 21 postsyn.; this study: 9 presyn., 115 postsyn.).

### Signal correlation and feature weight similarity independently contribute to connectivity.

**Figure 5.**
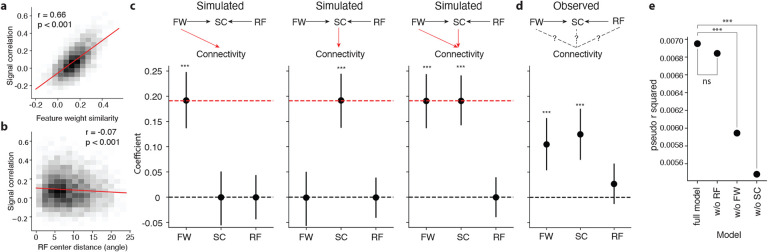
Feature weight similarity predicts synaptic connectivity beyond signal correlation. **a, b**, Signal correlation is strongly correlated with feature weight similarity (**a**, two-sample t-test) and weakly anti-correlated with receptive field (RF) center distance (**b**, two-sample t-test). **c**, After simulating connectivity graphs in which pairwise functional properties were preserved (top row) but with injected causal relationships (red arrows) between connectivity and feature weight similarity (FW, left), signal correlation (SC, center), or both (right), logistic regression models trained to predict connectivity from SC, FW, and RF (bottom row) recover injected relationships in the model coefficients (mean ± 95% c.i. by Monte Carlo simulation, p values = difference from zero, red dotted line = ground truth contribution). **d**, Logistic regression model coefficients as in (**c**), fitted to observed connectivity data, revealing significant non-zero contribution from both SC and FW (mean ± 95% c.i. by MLE, p value by Wald test). The contributions from SC and FW were not significantly different from each other (likelihood ratio test (LRT), *p* = 0.062)). **e**, Logistic regression model connectivity prediction performance on observed connectivity data (measured as likelihood relative to an intercept-only model) for three models trained on only two of the three functional pairwise properties (FW + RF, SC + RF, SC + FW, left) versus the model trained on all three properties (SC + FW + RF, right). Removing SC and FW both significantly reduce model performance (likelihood ratio test). (* = p-value < 0.05, ** = p-value < 0.01, * * * = p-value < 0.001).

We next examined the relationships between pairwise functional properties. Signal correlation is correlated to feature weight similarity (pearson r=0.66, p < 0.001) (Fig. 5a), and weakly anti-correlated to receptive field center distance (pearson r=−0.08, p < 0.001) (Fig. 5b). To test whether *in silico* signal correlation and feature weight similarity both independently contribute to higher connection probability, we used a logistic regression model to analyze the relationship between feature weight, signal correlation and connectivity. We first tested the efficacy of our selected model on simulated data with known effect sizes. A graph of potential connectivity was constructed, where nodes are neurons, and edges were assigned between neurons with an observed synapse or ADP in the dataset. For each edge, observed pairwise signal correlation (SC), feature weight similarity (FW), and receptive field center distance (RF) were inherited from the corresponding neurons. In three toy models, synapses in the graph were stochastically simulated at the overall observed connectivity rate (Fig. 5c, red dotted line), with synapse probability determined per ADP by feature weight similarity alone (Fig. 5c, left), signal correlation alone (Fig. 5c, center), or an equal contribution of signal correlation and feature weight similarity (Fig. 5c, right). We are able to recover the simulated contributions through the estimated coefficients of the logistic regression model (*connectivity* ~ *SC* + *F W* + *RF* , *p* < 0.001 by Monte-Carlo simulation) despite the high correlation between SC and FW (Fig. 5c). Interestingly, when we applied the logistic regression model to the observed connectivity, we found that the coefficients of both signal correlation and feature weight were statistically significant (Fig. 5d, coefficients significantly different from zero, Wald test, *p* < 0.001 for both SC and FW, *p* = 0.091 for RF). Additionally, we found that including both signal correlation and feature weight similarity as predictors in the model significantly improved prediction accuracy compared to models in which either one was excluded (Fig. 5e, likelihood ratio test, *p* < 0.001 for reducing SC, *p* < 0.001 for reducing FW, *p* = 0.092 for reducing RF). Overall, our results show that both signal correlation and feature weight similarity carry independent information about the connection probability between neurons, that could not be fully captured by either metric alone.

## Discussion

Discovering the principles that relate structure to function is central in the pursuit of a circuit-level mechanistic understanding of brain computations. Here, we used the MICrONS multi-area dataset — the largest of its kind — to study the relationship between the connections and functional responses of excitatory neurons in mouse visual cortex across cortical layers and visual areas. Our findings revealed that neurons with highly correlated responses to natural videos (i.e. high signal correlations) tended to be connected with each other, not only within the same cortical areas but also across multiple layers and visual areas, including feedforward and feedback connections. While the overall principle of “like-to-like” connectivity that we describe here is consistent with a number of previous studies, this work leverages three unique strengths of the MICrONS dataset to extend and refine these previous findings.

First, the **scale of the volume** enabled us to look at connection principles across all layers of cortex, not just within V1, but also in projections between V1 and higher visual areas. In agreement with previous findings from V1 L2/3, we found that pairs of cells with higher signal correlations were more likely to be connected (Ko et al., 2011; Cossell et al., 2015). This general principle held not just in V1 L2/3, but also in higher visual areas and for interarea feedforward and feedback projections.

Second, we were able to take advantage of the **dense reconstruction** to compute a set of null distributions for the expected connectivity between neurons. These controls enable us to distinguish whether the relationships we observed between connectivity and function are due to the overall geometry of axonal and dendritic arbors in the volume, or whether they reflect a more precise connectivity rule at the level of individual synapses. For example, it is only with the inclusion of both same region and ADP controls that we are able to observe the diverging findings of axon trajectory level selectivity for receptive field center distance (Fig. 3 d, e, f) and synaptic level selectivity for feature weight similarity (Fig. 3 a, b, c). These different controls can be mapped onto potential developmental or adult plasticity mechanisms that may shape the coarse axon trajectory and fine-scale synaptic connectivity across the brain.

Finally, our **deep learning neural predictive modeling approach** enabled us to not only comprehensively characterize signal correlation, but also to separate (i.e. factorize) neuronal tuning into spatial and feature tuning components. While the model feature weights represent the feature tuning preferences of a given neuron, signal correlation represents how those feature tuning preferences interact with the statistics of the stimulus set used to measure them. Although these two metrics are correlated, by comparing the relationship to connectivity in cases where feature weight similarity and signal correlation diverge, we can attempt to separate the contributions of feature tuning (e.g. “like-to-like”) and coincident activity (e.g. “fire together, wire together”) to how neural circuits are wired. In fact, we find that signal correlation and feature weight similarity do independently contribute to predictions of connectivity between pairs of neurons. The causes of these independent contributions provide the opportunity for some interesting speculation. For example, both feature weight similarity and signal correlation could predict connectivity arising from coincident activity due to features within the overlapping classical receptive field. However, the independent contribution of signal correlation may be due to long range spatiotemporal correlations in natural scene statistics (Simoncelli and Olshausen, 2003), including those beyond the range of the classical receptive field. This is because under the “fire together, wire together” hypothesis, the observed synapses are due to the coincident firing of neuron ensembles shaped by the statistics of the lifetime visual experience of the animal, which we mimic with the statistics of our natural dynamic stimuli. On the other hand, the independent contribution of feature weights may be a result of mechanisms that influence both neuronal tuning properties and connectivity. One example would be if functionally similar neurons are genetically predestined to form more synapses between them.

A more banal explanation might be that one could falsely detect an independent contribution when two similarity metrics are correlated — as one metric fails to capture the functional similarity between a neuron pair, the second metric provides the missing information. One potential cause would be if our similarity metrics inadequately capture the relevant features influencing connectivity. For example, to the extent that the high dimensional feature representation in the digital twin suffers from a redundant (i.e. non-identifiable) embedding, decreasing predictive power of feature weight cosine similarity may emerge as an apparently independent signal correlation contribution. While the focus in this work was on creating a model optimized for predictive performance, and model training included dropout (Srivastava et al., 2014) which has been shown to decorrelate features in neural network (Cogswell et al., 2015), future work might improve by also including model architecture and training regime changes guaranteeing a non-redundant penultimate layer of feature weights. Alternatively, if the *in vivo* or *in silico* stimulus poorly approximates the lifetime statistics of the animal’s visual experience, it may result in an apparently independent feature weight similarity contribution. To mitigate this, the MICrONS dataset utilized a high entropy, natural video stimulus and hours-long recordings to characterize functional properties, although future work could expand in this dimension by continuing to design more immersive or ethologically linked recording conditions (Froudarakis et al., 2014; Hoy et al., 2016; Parker et al., 2022)

In order to compare with previous work, we also used the digital twin to extract a more classical form of feature tuning preference, orientation tuning. However, in contrast to previous studies (Lee et al., 2016; Ko et al., 2011), we did not see a significant relationship between orientation tuning and connection probability, except at the axon trajectory level. This may be due to practical differences, such as the parametric stimulus used to characterize orientation tuning (*in silico* drifting noise with orientation coherence in our study versus drifting gratings), or the location and size of the area being studied (anterolateral V1 and HVA in our study versus posterior V1 in Lee et al. 2016 and monocular V1 in Ko et al. 2011). In the case of the latter, previous work has described an orientation tuning bias across V1 (Fahey et al., 2019). As a consequence, the same connectivity rule may be more difficult to observe under different orientation biases in different parts of V1, for example if the presynaptic or postsynaptic population was unusually homogeneous with respect to preferred orientation. It is also possible that connectivity rules might differ across V1. However, given that stimuli optimized to drive the responses of neurons even in mouse V1 exhibit complex spatial features deviating strikingly from Gabor-like stimuli (Walker et al., 2019), this may highlight the advantages of studying more complete tuning functions, such as the model feature weights, that go beyond classical orientation preference.

Lastly, many of the relationships we describe here, while statistically significant, have an apparently small effect size. One possibility is that small effects, applied broadly in the context of large neural populations, may have emergent effects across the circuit with large consequences. Another orthogonal possibility is that the small effect sizes we see here are actually the average of more complicated rules that net out to a small effect in aggregate. Future work could address this by expanding the descriptive model to take into account additional features that inform the likelihood of a synapse for a particular presynaptic-postsynaptic pair, such as transcriptomic / morphological features, role in higher order circuit motifs, or location of the synapse opportunity within the arbor.

This work provides a first glimpse of principles of cortical organization that can be discovered with large datasets combining detailed functional characterization with synaptic-scale connectivity. While the incredible accuracy of machine learning-based reconstruction methods has rightly increased optimism about the potential discoveries that can be made from large EM volumes — especially when combined with functional characterization — we should also not forget the magnitude of the challenge contained in even a 1mm^3^ volume of mouse cortex. The analyses in this paper are based on only a small number of manually proofread neurons, but even this limited view of the dataset represents an impressive volume of axonal and dendritic reconstruction. Ongoing investments in proofreading, matching, and extension efforts within this volume will have exponential returns for future analyses as they yield a more complete functional connectomic graph. There is much more to discover about this relationship from this dataset, and others like it that are currently in preparation. Our hope is that this dataset, including both the structural anatomy and the immortalized digital twin for ongoing *in silico* experiments, will be a community resource that will yield both concrete insights as well as inspiration about the scale of investigation that is now possible in Neuroscience.

## Methods

### MICrONS Dataset.

MICrONS dataset was collected as described in MICrONS Consortium et al. (2021), including neurophysiological data collection, visual stimulation, stimulus composition, EM data collection, automatic EM segmentation and reconstruction, manual EM proofreading, volume coregistration, and manual soma-soma matching between the functional and EM volumes. Neurophysiological experiments, Visual Stimulution,and Stimulus Composition sections below are specific to additional experiments described in Supp. Fig. 4.

### Neurophysiological experiments.

All procedures were approved by the Institutional Animal Care and Use Committee of Baylor College of Medicine. Three mice (Mus musculus, 1 female, 2 males, 78–86 days old at first experimental scan) expressing GCaMP6s in excitatory neurons via Slc17a7-Cre and Ai162 transgenic lines (recommended and generously shared by Hongkui Zeng at Allen Institute for Brain Science; JAX stock 023527 and 031562, respectively) were anesthetized and a 4 mm craniotomy was made over the visual cortex of the right hemisphere as described previously (Reimer et al., 2014; Froudarakis et al., 2014).

For additional experiments, mice were head-mounted above a cylindrical treadmill and calcium imaging was performed with an experimental mesoscope (Sofroniew et al., 2016) as described in release (MICrONS Consortium et al., 2021), with surface power not exceeding 20 mW, depth constant of 220 μm, and greatest laser power of ~ 86 mW was used at approximately 400 μm from the surface.

The craniotomy window was leveled with regards to the objective with six degrees of freedom. Pixel-wise responses from an ROI spanning the cortical window (3600 × 4000 μm, 0.2 px/μm, 200 μm from surface, 2.5 Hz) to drifting bar stimuli were used to generate a sign map for delineating visual areas (Garrett et al., 2014).

For the orientation tuning validation data in Supp. Fig. 4, our target imaging site was a 1200 × 1100μm^2^ area spanning L2-L5 at the conjunction of lateral primary visual cortex (V1) and three lateral higher visual areas: anterolateral (AL), lateromedial (LM), and rostrolateral (RL). This resulted in an imaging volume that was roughly 50% V1 and 50% higher visual area. This target was chosen in order to mimic the area membership and functional property distribution in the MICrONS animal. Each scan was performed at 6.3 Hz, collecting eight 620 × 1100μm^2^ fields per frame at 0.4 px*/*μm xy resolution to tile a 1190 − 1200 × 1100μm^2^ FOV at four depths (two planes per depth, 40 − 50μm overlap between coplanar fields). The four imaging planes were distributed across layers with at least 50μm spacing, with two planes in L2/3 (depths: 180μm,230μm), one in L4 (325μm), and one in L5 (400μm).

Movie of the animal’s eye and face was captured throughout the experiment. A hot mirror (Thorlabs FM02) positioned between the animal’s left eye and the stimulus monitor was used to reflect an IR image onto a camera (Genie Nano C1920M, Teledyne Dalsa) without obscuring the visual stimulus. The position of the mirror and camera were manually calibrated per session and focused on the pupil. Field of view was manually cropped for each session. The field of view contained the left eye in its entirety, 250–310 pixels height × 350–400 pixels width at 20 Hz. Frame times were time stamped in the behavioral clock for alignment to the stimulus and scan frame times. Video was compressed using Labview’s MJPEG codec with quality constant of 600 and stored the frames in AVI file.

Light diffusing from the laser during scanning through the pupil was used to capture pupil diameter and eye movements. A DeepLabCut model (Mathis et al., 2018) was trained on 17 manually labeled samples from 11 animals to label each frame of the compressed eye video (intraframe only H.264 compression, CRF:17) with 8 eyelid points and 8 pupil points at cardinal and intercardinal positions. Pupil points with likelihood >0.9 (all 8 in 69.8–91.0% of frames per scan) were fit with the smallest enclosing circle, and the radius and center of this circle was extracted. Frames with < 3 pupil points with likelihood >0.9 (<0.5% frames per scan), or producing a circle fit with outlier > 5.5 standard deviations from the mean in any of the three parameters (center x, center y, radius, <0.1% frames per scan) were discarded (total <0.6% frames per scan). Gaps of <= 10 discarded frames were replaced by linear interpolation. Trials affected by remaining gaps were discarded (<4 trials per scan, <0.5%).

The mouse was head-restrained during imaging but could walk on a treadmill. Rostro-caudal treadmill movement was measured using a rotary optical encoder (Accu-Coder 15T-01SF-2000NV1ROC-F03-S1) with a resolution of 8000 pulses per revolution, and was recorded at ~100 Hz in order to extract locomotion velocity.

### Visual stimulation.

For the orientation tuning validation data in Supp. Fig. 4, monitor size and positioning relative to the mouse were as described in MICrONS Consortium et al. (2021), with the exception of replacing the dot stimulus with 10 × 10 grid tiling a central square (approx 90 degrees width and height) with 10 repetitions of 200 ms presentation at each location.

A photodiode (TAOS TSL253) was sealed to the top left corner of the monitor, and the voltage was recorded at 10 KHz and timestamped with a 10 MHz behavior clock. Simultaneous measurement with a luminance meter (LS-100 Konica Minolta) perpendicular to and targeting the center of the monitor was used to generate a lookup table for linear interpolation between photodiode voltage and monitor luminance in cd/m^2^ for 16 equidistant values from 0–255, and one baseline value with the monitor unpowered.

At the beginning of each experimental session, we collected photodiode voltage for 52 full-screen pixel values from 0 to 255 for one second trials. The mean photodiode voltage for each trial was collected with an 800 ms boxcar window with 200 ms offset. The voltage was converted to luminance using previously measured relationship between photodiode voltage and luminance and the resulting luminance vs voltage curve was fit with the function *L* = *B* +*A·P*^*γ*^ where L is the measured luminance for pixel value P, and the *γ* of the monitor was fit as 1.73. All stimuli were shown without linearizing the monitor (i.e. with monitor in normal gamma mode).

During the stimulus presentation, display frame sequence information was encoded in a 3 level signal, derived from the photodiode, according to the binary encoding of the display frame (flip) number assigned in-order. This signal underwent a sine convolution, allowing for local peak detection to recover the binary signal together with its behavioral time stamps. The encoded binary signal was reconstructed for >93% of the flips. Each flip was time stamped by a stimulus clock (MasterClock PCIe-OSC-HSO-2 card). A linear fit was applied to the flip timestamps in the behavioral and stimulus clocks, and the parameters of that fit were used to align stimulus display frames with scanner and camera frames. The mean photodiode voltage of the sequence encoding signal at pixel values 0 and 255 was used to estimate the luminance range of the monitor during the stimulus, with minimum values of approximately 0.005 cd/m^2^ and maximum values of approximately 9.0 cd/m^2^.

### Stimulus Composition.

Dynamic stimuli libraries of natural movies, global directional parametric stimuli (“Monet”), and local directional parametric stimuli (“Trippy”), are as described in MICrONS Consortium et al. (2021). In addition to the 84 minutes of trials as described in MICrONS Consortium et al. (2021), each stimulus contained an additional 40 minutes of trials, randomly intermixed, as follows:

- **Unique Global Directional Parametric Stimulus (“Monet”):** 120 seeds, 15 seconds each, 1 repeat per scan, 30 minutes total. Seeds conserved across all scans.
- **Oracle Global Directional Parametric Stimulus (“Monet”):** 4 seeds, 15 seconds each, 10 repeats, 10 minutes total. Seeds conserved across all scans.

### Preprocessing of neural responses and behavioral data.

Fluorescence traces from the MICrONS dataset and the additional data for Supp. Fig. 4 were detrended, deconvolved, and aligned to stimulus and behavior as described in Wang et al. (2023), and all traces were resampled at 29.967 Hz. Possible redundant traces, where a single neuron produced segmented masks in multiple imaging fields, were all kept for downstream model training. We elected to remove one of the 14 released scans from the analysis due to compromised optics (water ran out from under the objective for ~ 20 minutes), leaving 13 scans. Trials with more than 10 consecutive untracked pupil frames were discarded (18–180 trials per scan, 2–39%).

### Model architecture.

Model architecture was similar to Wang et al. (2023) with the following differences in the core component of the neural network:

- a feedforward network with 7 3D convolutional layers with an ELU nonlinearity, instead of 3 layers with a GeLU nonlinearity.
- a recurrent network with a Conv-LSTM architecture, instead of the newly proposed recurrent vision transformer (RvT) architecture.

### Model training of digital twin.

We utilized transfer learning to train the digital twin model as described in Wang et al. (2023). Briefly, the core network of the models was trained on 8 scans collected from 8 mice to capture cortical representations of visual stimuli shared across mice. The parameters of the core network are then frozen and the rest of the network parameters are trained for each scan in the MICrONS dataset independently.

### Functional unit inclusion criteria.

In order to focus our analyses on neurons that are visually responsive and well modeled by the digital twin, we applied a dual functional threshold over two metrics prior to all analyses related to signal correlation, receptive field center distance, and feature weight similarity.

#### In vivo reliability threshold:

In order to estimate the reliability of neuronal responses to visual stimuli, we computed the upper bound of correlation coefficient (*CC*_*max*_, Schoppe et al. 2016) across 60 seconds of natural movie stimuli repeated 10 times across the stimulus period (10 min total). *CC*_*max*_ was computed as:

$$CC_{\text{max}}=\sqrt{\frac{\text{NVar}(\bar{y})-\bar{\text{Var}(y)}}{\left(N-1)\text{Var}(\bar{y}\right)}},$$

where *y* is the *in vivo* responses, and *N* is the number of trials. A threshold of *CC*_*max*_ > 0.6 was applied.

#### Model prediction performance threshold:

In order to focus our analyses on neurons for which adequate model performance indicated sufficiently accurate representation of the neuronal tuning features, we computed the test correlation coefficient on the withheld oracle test dataset, which was not part of the training set. Test correlation coefficient (*CC*_*abs*_) was computed as:

$$CC_{\text{abs}}=\frac{\text{Cov}(\bar{x},\bar{y})}{\sqrt{\text{Var}(\bar{x})\text{Var}(\bar{y})}},$$

where *x* is the *in silico* response and *y* is the *in vivo* response. A threshold of *CC*_*abs*_ > 0.35 was applied.

122 out of 152 presynaptic neurons and 1975 out of 5502 postsynaptic neurons passed the dual functional unit inclusion criteria.

#### Oracle score:

Lastly, the oracle score was computed for all units as described in (MICrONS Consortium et al., 2021). Where more than one two-photon functional unit was matched to a given EM unit, the functional trace with the higher oracle score was used for analysis.

### Anatomical controls.

In order to control for anatomy at the coarse axon projection level (“same region” control), we recruited all visually responsive, well predicted, matched excitatory neurons (*CC*_*max*_ > 0.6, *CC*_*abs*_ > 0.35, EM *→* 2P soma matched) that are located in the same region as the postsynaptic target, but are not observed to form a synapse with the presynaptic neuron. Area membership labels per neuron were used from the MICrONS release (MICrONS Consortium et al., 2021). Additionally, control candidates that meet criteria for both the same region control and the ADP control will only be included in ADP control.

In order to control for anatomy at the finer synaptic level (“ADP” control), we recruited all visually responsive, well predicted, matched excitatory neurons (*CC*_*max*_ > 0.6, *CC*_*abs*_ > 0.35, EM *→* 2P soma matched) with a dendritic skeleton passing within 5μm of the presynaptic neuron axonal skeleton and also within 10μm of at least one synapse in the presynaptic axonal arbor (3D euclidean distance), but are not observed to form a synapse with the presynaptic neuron. Presynaptic axonal skeletons were computed using the pcg_skel package developed by collaborators at the Allen Institute for Brain Science (Schneider-Mizell et al., 2023; Schneider-Mizell and Collman, 2023). For postsynaptic dendritic skeletons, we used the automatically proofread and skeletonized dendritic arbors as described in Celii et al. (2023). ADP detection was also run as described in Celii et al. (2023), with the exception of using pcg_skel presynaptic skeletons as described above.

In the case of the joint area and layer analysis (Fig. 4), candidates in both the “same region” and “ADP” controls must additionally match the same layer classification as the postsynaptic target in order to be included. Layer membership was classified by depth of imaged soma respect to the dura in the structural two-photon stack: L1: 0 − 98μm; L2/3: 98 − 283μm; L4: 283 − 371μm; L5: 371 − 574μm; L6: 574 − 713μm.

### Measuring functional similarities.

#### In silico response correlations.

To characterize the pair-wise tuning similarity between two modeled neurons, we computed the Pearson correlation of their responses to 2500 seconds of natural movies. The natural movies were fed in to the model as trials of 10 sec. Model responses were generated at 29.967 Hz and Pearson correlations were computed after binning the responses into 500msec non-overlapping bins and concatenating across trials.

#### In silico feature weight similarity and receptive field center distance.

The digital twin model architecture includes a shared core which is trained to represent spatiotemporal features in the stimulus input, and a final layer where the spatiotemporal features at a specific readout location are linearly weighted in order to produce the predicted activity of a specific neuron at the current time point Wang et al. (2023). The readout location and linear feature weight are independently learned for each neuron. In order to measure the feature weight similarity between two units, we extract the linear feature weights from this final step as vector of length 512, and take the cosine similarity between the two vectors. In order to measure the receptive field center distance between two units, we extract the readout location as 2D coordinates on the monitor, and take the angle between them with respect to the mouse’s eye, assuming the monitor is centered on, 15 cm away from, and normal to the surface of the mouse’s eye at the closest point.

#### In silico difference in preferred orientation.

240 blocks of parametric directional visual stimuli (“Monet”) are shown to the model, with each fifteen second block consisting of 16 trials of equally distributed and randomly ordered unique directions of motion between 0–360 degrees. A modeled neuron’s direction tuning curve is computed as its mean responses to 16 directions averaged across blocks. We calculated the global orientation selectivity index (gOSI) from the modeled neuron’s tuning curve as follows:

(1) $$\text{gOSI}=\frac{\Sigma R_{\theta }e^{2i\theta }}{\Sigma R_{\theta }}$$

where *θ* is the direction of the stimulus and *R*_*θ*_ is the mean modeled response to the stimulus at direction *θ*. Only neurons with *gOSI* > 0.25 were included in the analyses in this paper. Unit-wise direction tuning curves are then modeled by a bivariate von Mises function with an offset:

(2) $$f(\theta |\mu ,\kappa ,p)=\frac{1}{2\pi I_{0}(\kappa )}\{p\text{exp}(\kappa \text{cos}(\theta -\mu ))\;+(1-p)\text{exp}(-\kappa \text{cos}(\theta -\mu ))\}\;+b$$

where *I*_0_ is the modified Bessel function, *μ* is the preferred direction, *κ* measures the concentration of the two peaks (larger *κ* means higher peaks thus higher orientation selectivity), *p* measures the relative height of the two peaks (*p* = 0.5 means two peaks of the same height, when *p* approaches 0 or 1, the bi-modal distribution reduces to a uni-model von Mises distribution), *b* is the offset. *μ*, *κ*, *p*, and *b* are fit by minimizing least squared error. The preferred orientation of a neuron is taken as the modulus of *μ* to 180 degrees.

In three scans not included in the MICrONS release, we characterized both the *in vivo* orientation tuning in response to 30 minutes of global directional parametric stimulus (“Monet”, Supp. Fig. 4a), as well as the *in silico* orientation tuning as described above for digital twin models with shared cores and readouts trained on neurons from the same scans, in response to stimuli matching the composition and duration of the MICrONS release scans (Supp. Fig. 4b). When we applied a threshold of *gOSI* > 0.25, we found that 95% of cells had an absolute difference between their *in silico* and *in vivo* preferred orientations less than 9.77deg.

### Statistical analysis of functional similarities and connectivity.

To compare functional similarities among the three neuron pair populations (connected neuron populations and two control neuron populations), independent t-tests were performed, with the Benjamini-Hochberg (BH) procedure used to correct for multiple comparisons. For region breakout analysis, multiple comparisons across four groups (V1 *→* V1, HVA *→* HVA, V1 *→* HVA, HVA *→* V1) and two control designs (connected v.s. same region control and connected v.s. ADP) were accounted for (total eight groups). For layer breakout analysis, we started with six presynaptic/postsynaptic groups (two regions; L2/3, L4, L5; total 36 groups) and two control comparisons (connected vs same region control, connected vs ADP; total 72 groups), and only groups with >10 connected neuron pairs were included in the analysis and accounted for in multiple comparisons (58 for Fig. 4a–f, 50 for Fig. 4g, h). To quantify fold changes in connection probability as a function of functional similarities, we followed these steps: 1) We binned all neuron pairs by their functional similarities (signal correlation, feature weight similarity, RF center distance, or difference in preferred orientation). 2) We calculated connection probability within each bin as the fraction of connected neuron pairs out of the total number of connected and control neuron pairs. 3) We normalized the connection probability by overall connection probability across all bins. Only bins with more than 10 connected neuron pairs and more than 2.5% of all connected neuron pairs are included in the analysis. To estimate standard deviation of fold changes in connection probability, we resampled the connected and control neuron pairs with replacement, binned the resampled distribution, and calculated the standard deviation of fold change in connection probability within each bin.

Pearson correlation coefficients were used to quantify relationships between functional similarities and cleft volume sizes. P values of two-sided tests on the Pearson correlation coefficients were reported. To test if multi-synaptic connected neuronal pairs share more similar functional properties when compared to ADP controls for spatial proximity, we grouped all connected neuron pairs and ADP neuron pairs into two groups: single synapse/ADP contact and multiple synapse/ADP contacts. Two-way ANOVA is performed to test whether functional similarity changes significantly across the interaction term of connectivity (synapses vs ADPs) and number of contacts (single vs multiple).

### Simulated connectivity graphs.

A graph of potential connectivity was constructed, where vertices are all visually responsive, well predicted, matched excitatory neurons (*CC*_max_ > 0.6, *CC*_abs_ > 0.35, EM *→* 2P soma matched), and vertices are connected by an edge if the pair of neurons has an observed synapse or ADP in the dataset. For each edge, we included the pairwise signal correlation (SC), feature weight similarity (FW), and receptive field center distance (RF) from the pair of corresponding vertices as potential causal variables for connectivity. Thus the colinearities among them were kept the same as the observed data. Fisher transformations were applied to SC and FW to Gaussianize the respective marginal distributions. Transformed SC, FW, and RF are then mean-subtracted and scaled by standard deviations for simulation and logistic regression analysis downstream.

The simulated connection probabilities were determined by three causal models: 1). signal correlation alone (Fig. 5c, left), 2). feature weight similarity alone (Fig. 5c, center), or 3). an equal contribution of signal correlation and feature weight similarity (Fig. 5c, right) through a logistic function

$$p_{\text{sim}}=\frac{1}{1+e^{-(Ax+B)}}$$,

where *p*_*sim*_ is the simulated connection probability, *x* is the determining functional property, *A* is the coefficient of the logistic regression *p* ~ *SC* fit on the observed data and *B* is optimized such that the simulated overall connection probability matches the observed overall connection probability.

Lastly, we sample 1000 simulated connectivity graphs per causal model. Each simulated graph is generated by stochastically sampling edges according to the edge probability. For each simulated graph, we fitted a multivariable logistic regression to predict connectivity probability between two vertices and included the SC, FW, and RF from the pair of corresponding vertices as covariates (*p* ~ *SC* + *F W* + *RF*). We derived the 95% confidence intervals of the coefficients for each causal model from empirical distributions of the coefficients across simulations.

### Logistic regression analysis of connectivity prediction.

We modeled the connection probability between two neurons as a multivariable logistic regression of form *p* ~ *SC* + *F W* + *RF*, with three coefficients corresponding to the signal correlation, feature weight similarity, and receptive field center distance between the two neurons. Mean and variance of the coefficients are estimated through Maximum Likelihood Estimation (MLE). We next compared the full model with reduced models where each of the three variables (SC, FW, and RF) are removed from the model. Mc-Fadden’s pseudo-R-squared of the full model and the reduced models are reported. Likelihood ratio tests (LRT) were used to compare the performance of the full models to the reduced models in order to assess the significance and importance of each individual feature for connectivity prediction.

### Software.

Experiments and analysis are carried out with custom built data pipelines. The data pipeline is developed in Matlab and Python with the following tools: Psychtoolbox, ScanImage, DeepLabCut, CAIMAN, and Labview were used for data collection. DataJoint, MySQL, and CAVE were used for storing and managing data. Meshparty, NEURD, and pcg_skel were used for morphology analysis. Numpy, pandas, SciPy, statsmodels, scikit-learn, and PyTorch were used for model training and statistical analysis. Matplotlib, seaborn, HoloViews, Ipyvolume, and Neuroglancer were used for graphical visualization. Jupyter, Docker, and Kubernetes were used for code development and deployment.

## Supplementary Material

1

## Data Availability

All MICrONS data have already been released on BossDB (https://bossdb.org/project/microns-minnie, please also see https://www.microns-explorer.org/cortical-mm3 for details). Additional code and data including digital twin architecture, learned weights, and *in silico* similarity metrics will be made publicly available in an online repository latest upon journal publication. Please contact us if you would like to get access before that time.
