# Supplementary material for: Functional connectomics reveals general wiring rule in mouse visual cortex
Source: bioRxiv. 2024 Oct 14:2023.03.13.531369. Originally published 2023 Mar 14. Preprint. [Version 3] doi: 10.1101/2023.03.13.531369 (PMC10054929; doi:10.1101/2023.03.13.531369)
Supplement: 1 [file NIHPP2023.03.13.531369v3-supplement-1.pdf]

bioRxiv preprint doi: https://doi.org/10.1101/2023.03.13.531369; this version posted October 15, 2024. The copyright holder for this preprint (which was not certified by peer review) is the author/funder, who has granted bioRxiv a license to display the preprint in perpetuity. It is made available under aCC-BY 4.0 International license.

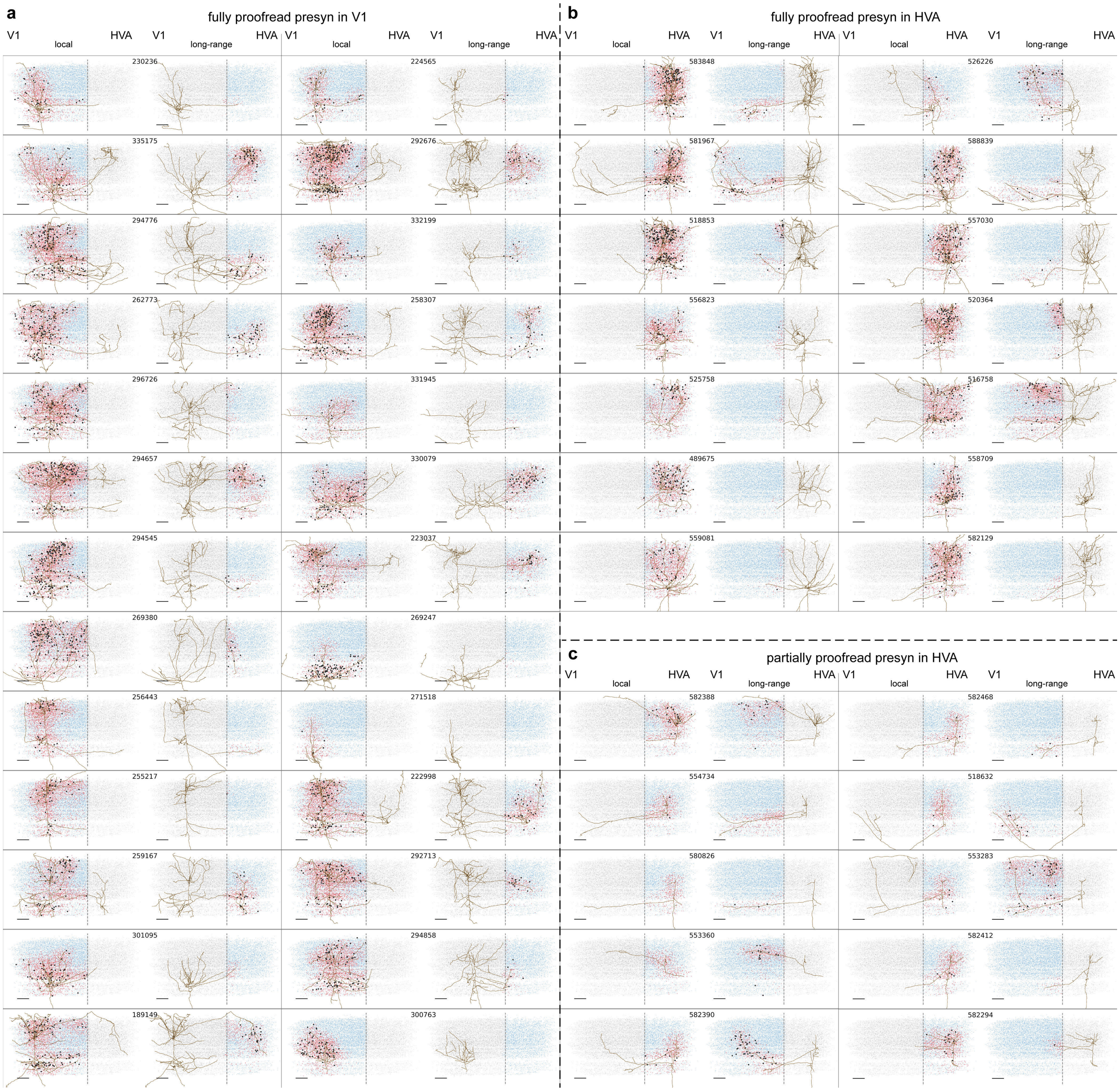

Supplemental Figure 1. Example proofread presynaptic axons in EM cortical space and their connected, ADP, and same region controls. The axon for every presynaptic (presyn) neuron is shown twice, once as a "local" projection type and again as a "long-range" type (even if the neuron has no local or long-range projections). The six digit ID from Table "nucleus_detection_v0" (MICrONS Consortium et al., 2021) is displayed above both plots. For each plot, the soma centroids of connected neurons, ADP controls, and same region controls are plotted in black, red, and blue, respectively. Gray dots are soma centroids of all other functionally matched neurons not used as controls for that presyn. The dashed gray line represents the V1-HVA boundary. Scale bar = $100\mu m$. **a**, Example fully proofread presynaptic axons with somas in V1. "Fully proofread" neurons are those where a proofreader attempted to extend every axonal branch to completion. **b**, Example fully proofread presynaptic axons with somas in HVA **c**, Example partially proofread presynaptic axons with somas in HVA. "Partially proofread" neurons are those where a proofreader only extended axonal branches that were pre-screened for whether they projected inter-areally (specifically to enrich for feedback connections).

bioRxiv preprint doi: https://doi.org/10.1101/2023.03.13.531369; this version posted October 15, 2024. The copyright holder for this preprint (which was not certified by peer review) is the author/funder, who has granted bioRxiv a license to display the preprint in perpetuity. It is made available under aCC-BY 4.0 International license.

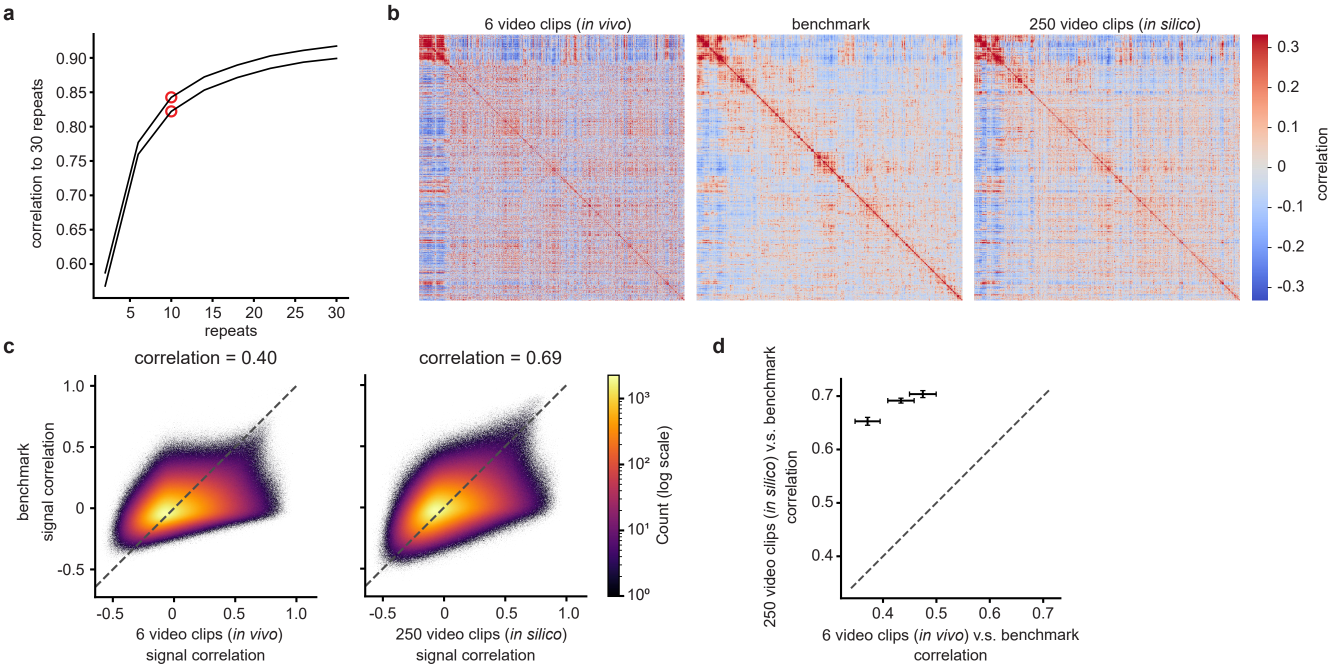

Supplemental Figure 2. The digital twin signal correlations align better with the *in vivo* benchmark than *in vivo* signal correlations generated with less data. **a**, Correlation of *in vivo* signal correlations generated with 6 video clips and varying numbers of repeats to *in vivo* signal correlations generated with 6 clips and 30 repeats, for two animals. 10 repeats (red marker) reasonably approximates the saturation point and is the number used for all other analyses. **b**, Signal correlation matrices of 1000 neurons generated from *in vivo* responses to 6 video clips (left), *in vivo* responses to 30 video clips (benchmark, middle) and digital twin responses to 250 video clips (*in silico*, right). The benchmark matrix is ordered by ward's hierarchical clustering. The *in vivo* and *in silico* signal correlation matrices are ordered in the same order as the benchmark matrix. The fine structure of the *in silico* matrix is qualitatively more similar to the benchmark than the *in vivo* matrix generated with 6 video clips is to the benchmark. **c**, 2D heatmaps of signal correlations from the benchmark (same benchmark as in **b**) vs *in vivo* responses to 6 video clips (left) and *in silico* responses to 250 clips (right). The correlation of *in silico* signal correlations to the benchmark is higher than the correlation of *in vivo* signal correlations generated with 6 video clips to the benchmark (0.69 vs 0.40). Colorbar: 2D bin counts in log scale. **d**, The correlation of *in silico* signal correlations to the benchmark vs the correlation of *in vivo* signal correlations generated with 6 video clips to the benchmark for three animals. Error bars are standard deviations estimated through resampling. All data points are in the upper left corner indicating that *in silico* signal correlations outperform *in vivo* signal correlations generated with 6 video clips. (p-value < 0.001 for all three animals)

bioRxiv preprint doi: https://doi.org/10.1101/2023.03.13.531369; this version posted October 15, 2024. The copyright holder for this preprint (which was not certified by peer review) is the author/funder, who has granted bioRxiv a license to display the preprint in perpetuity. It is made available under aCC-BY 4.0 International license.

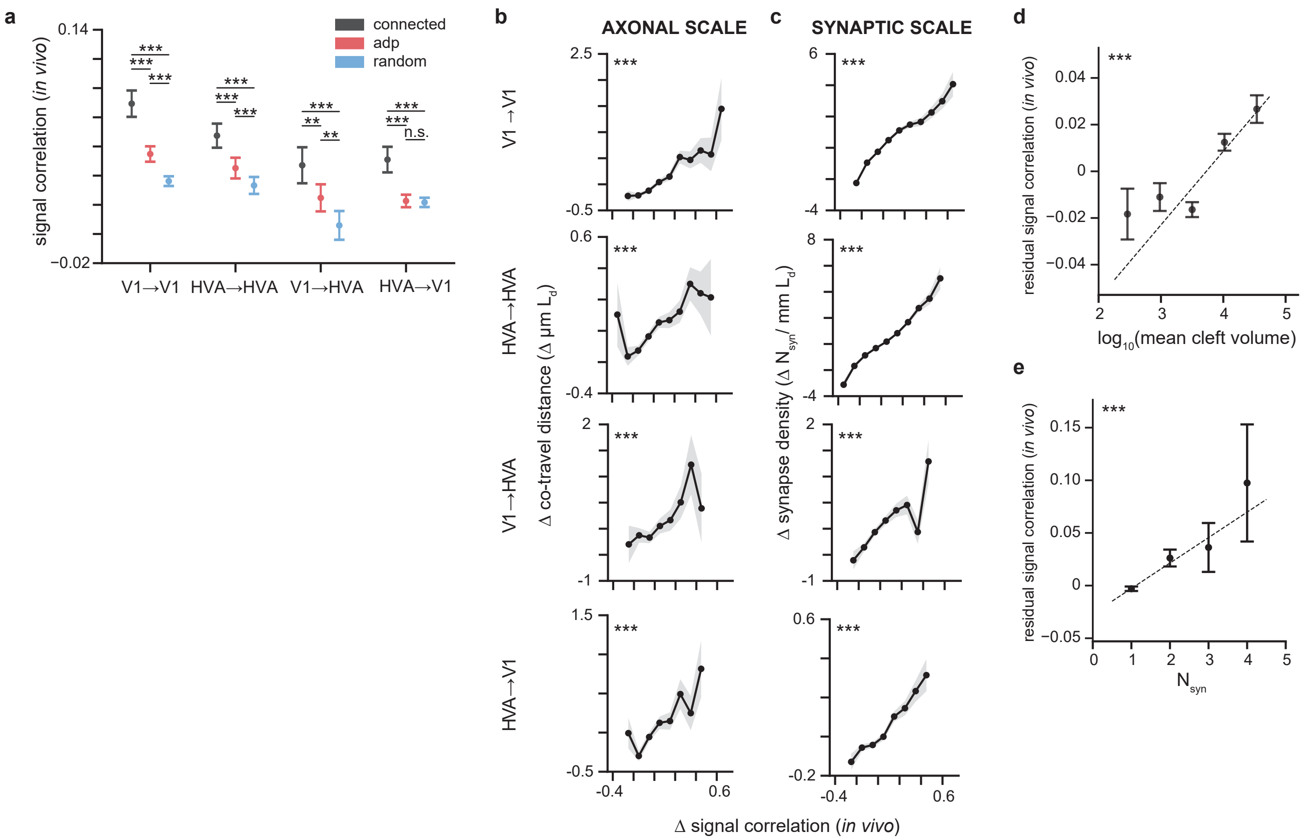

Supplemental Figure 3. Synaptic connectivity increases with empirical signal correlations measured directly *in vivo* rather than via the digital twin. **a**, Mean *in vivo* signal correlation is different (mean $\pm$ sem, paired t-test) for connected pairs, ADP controls, and same area controls for all projection types, as in Fig 2d. **b**, Axon-dendrite co-travel distance ($\mu m L_d$) increases in a graded fashion with *in vivo* signal correlation for all projection types, as in Fig 2e. **c** Synapse density (N_{syn}/mmL_d) increases in a graded fashion with signal correlation, for all projection types, as in Fig 2f. The shaded regions in **b** and **c** are bootstrap-based standard deviation. **d**, Synapse size (log_{10} cleft volume in voxels) is positively correlated with *in vivo* signal correlation after regressing out L_d (p-value by linear regression), as in Fig 2h. **e**, *In vivo* signal correlations increases with number of synapses after regressing out L_d (p-values by linear regression), as in Fig 2j. (For all panels, $*$ = p-value < 0.05, $**$ = p-value < 0.01, $***$ = p-value < 0.001, multiple comparison correction by BH procedure)

bioRxiv preprint doi: https://doi.org/10.1101/2023.03.13.531369; this version posted October 15, 2024. The copyright holder for this preprint (which was not certified by peer review) is the author/funder, who has granted bioRxiv a license to display the preprint in perpetuity. It is made available under aCC-BY 4.0 International license.

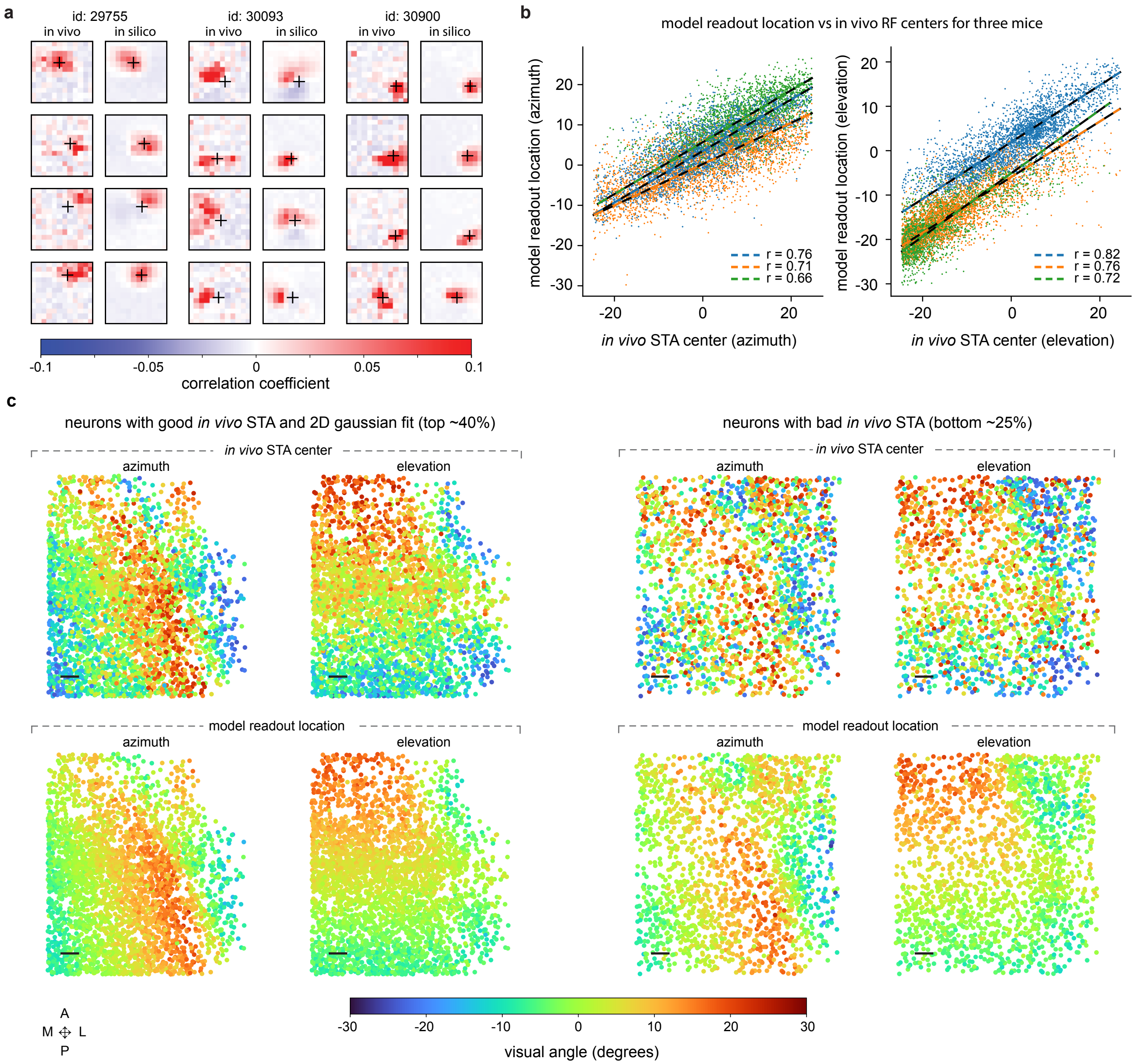

Supplemental Figure 4. Model readout center aligns with receptive field center measured *in vivo* with sparse noise stimuli. **a**, Visual comparison of STAs generated from *in vivo* responses to a sparse noise stimulus (left) vs STAs generated from *in silico* responses to the same stimulus (right) for three animals (blue, orange, and green). The black cross represents the model readout location. Examples are randomly chosen from the top ≈ 40% of neurons remaining after a threshold on *in vivo* STA quality is applied. **b**, Model readout location vs *in vivo* STA center for azimuth coordinate (left) and elevation coordinate (right). **c**, Retinotopic maps for animal id: 29755. Left: Maps generated with top ≈ 40% of neurons after an *in vivo* STA quality threshold is applied. Right: Maps for the bottom ≈ 25% of neurons. Top row: maps generated from *in vivo* STA's centers. Bottom row: maps generated from the digital twin model readout location. The maps generated from the model are qualitatively less noisy, even for maps generated from neurons with poor STA quality. Colorbar: degree of visual angle for both azimuth and elevation coordinates. Anatomical axes: A = anterior, P = posterior, M = medial, L = lateral. Scale bar: 100 μm.

bioRxiv preprint doi: https://doi.org/10.1101/2023.03.13.531369; this version posted October 15, 2024. The copyright holder for this preprint (which was not certified by peer review) is the author/funder, who has granted bioRxiv a license to display the preprint in perpetuity. It is made available under aCC-BY 4.0 International license.

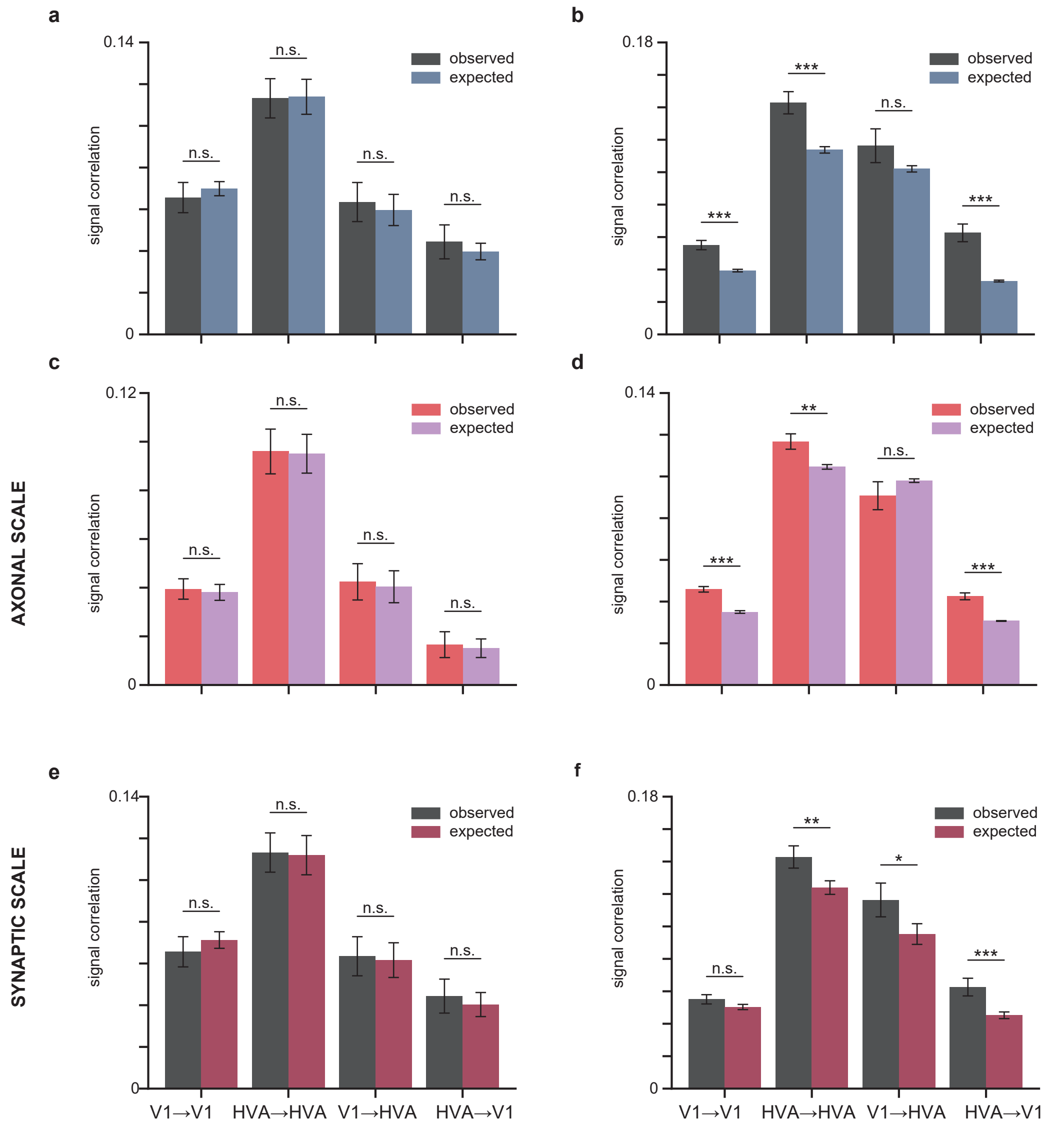

Supplemental Figure 5. Postsyns with a common input are more similar to each other than expected by a pairwise like-to-like rule at both axonal and synaptic scale. a, Mean pre-post signal correlations in the data (dark gray, "observed") and the model (blue, "expected") are not significantly different, indicating that the model reproduces the expected pairwise like-to-like rule **b**, Mean pairwise *in silico* signal correlation of postsyns, reproduced from Fig 5c. The observed data shows significantly higher postsyn to postsyn similarity than predicted by the model fit with only a pairwise rule, for three out of four projection types. **c**, As in **a**, but at “Axonal" scale. **d**, As in **b**, but at “Axonal" scale. **e**, As in **c**, but at “Synaptic" scale. **f**, As in **d**, but at “Synaptic" scale.

bioRxiv preprint doi: https://doi.org/10.1101/2023.03.13.531369; this version posted October 15, 2024. The copyright holder for this preprint (which was not certified by peer review) is the author/funder, who has granted bioRxiv a license to display the preprint in perpetuity. It is made available under aCC-BY 4.0 International license.

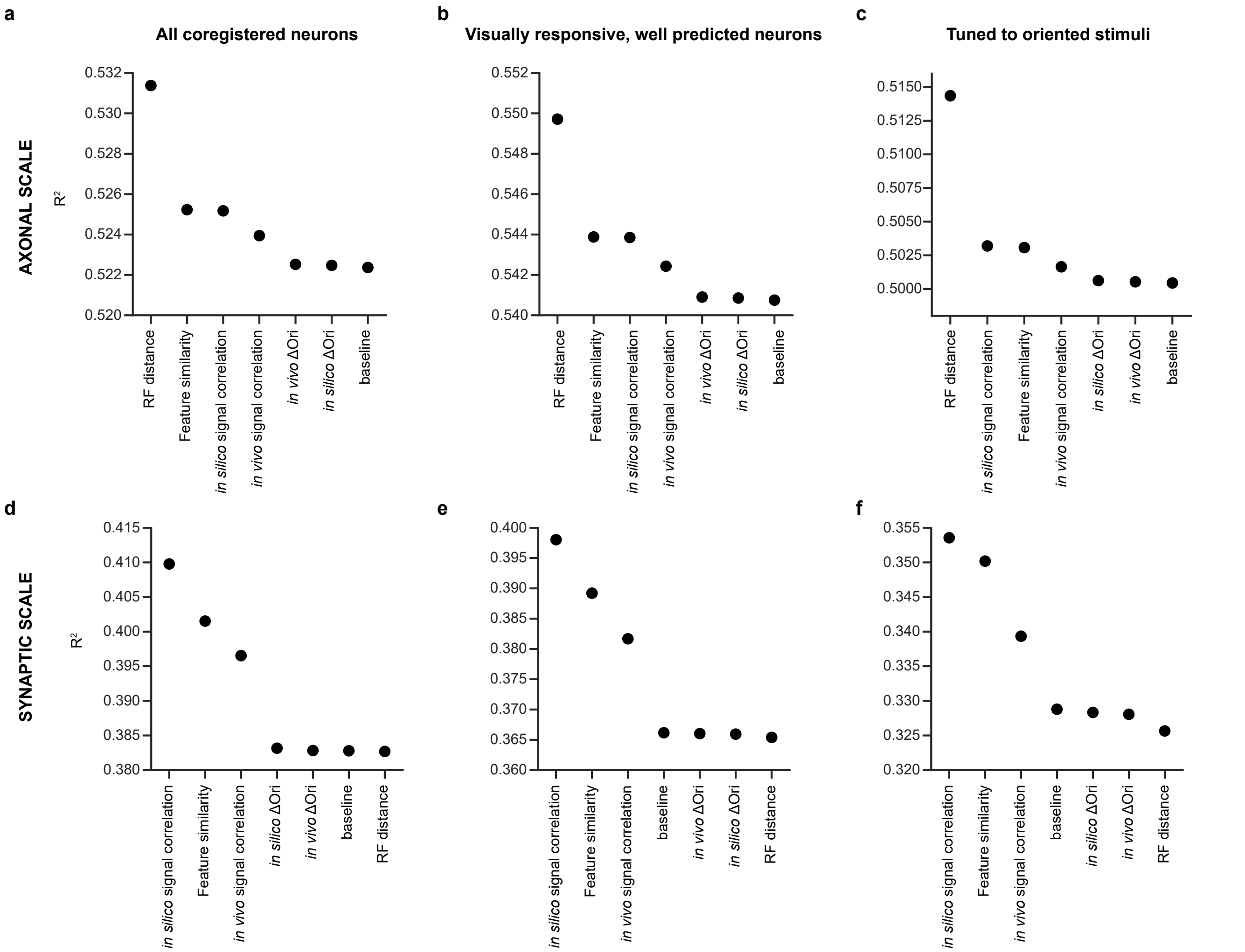

Supplemental Figure 6. Performance of various functional metrics in predicting axon-dendrite co-travel distance (L_d, Axonal scale) or synapse density (N_{syn}/mmL_d, Synaptic scale). Model performance of GLMMs (Nakagawa's conditional R^2) for predicting axon-dendrite co-travel distance (L_d): **a**, **b**, **c** and synapse density (N_{syn}/mmL_d): **d**, **e**, **f**, for all coregistered neurons: **a**, **d**, all visually responsive, well predicted neurons: **b**, **e**, and neurons tuned to oriented stimuli: **c**, **f**. The GLMMs are fit to predict axon-dendrite co-travel distance or synapse density independently with each functional metric, the projection type, and the interaction between the two while considering the interaction term of projection type and presynaptic neuron identity as random effects. The baseline models were not fitted with information about functional metrics. They predict axon-dendrite co-travel distance or synapse density with the projection type alone while considering the interaction term of projection type and presynaptic neuron identity as random effects.

bioRxiv preprint doi: https://doi.org/10.1101/2023.03.13.531369; this version posted October 15, 2024. The copyright holder for this preprint (which was not certified by peer review) is the author/funder, who has granted bioRxiv a license to display the preprint in perpetuity. It is made available under aCC-BY 4.0 International license.

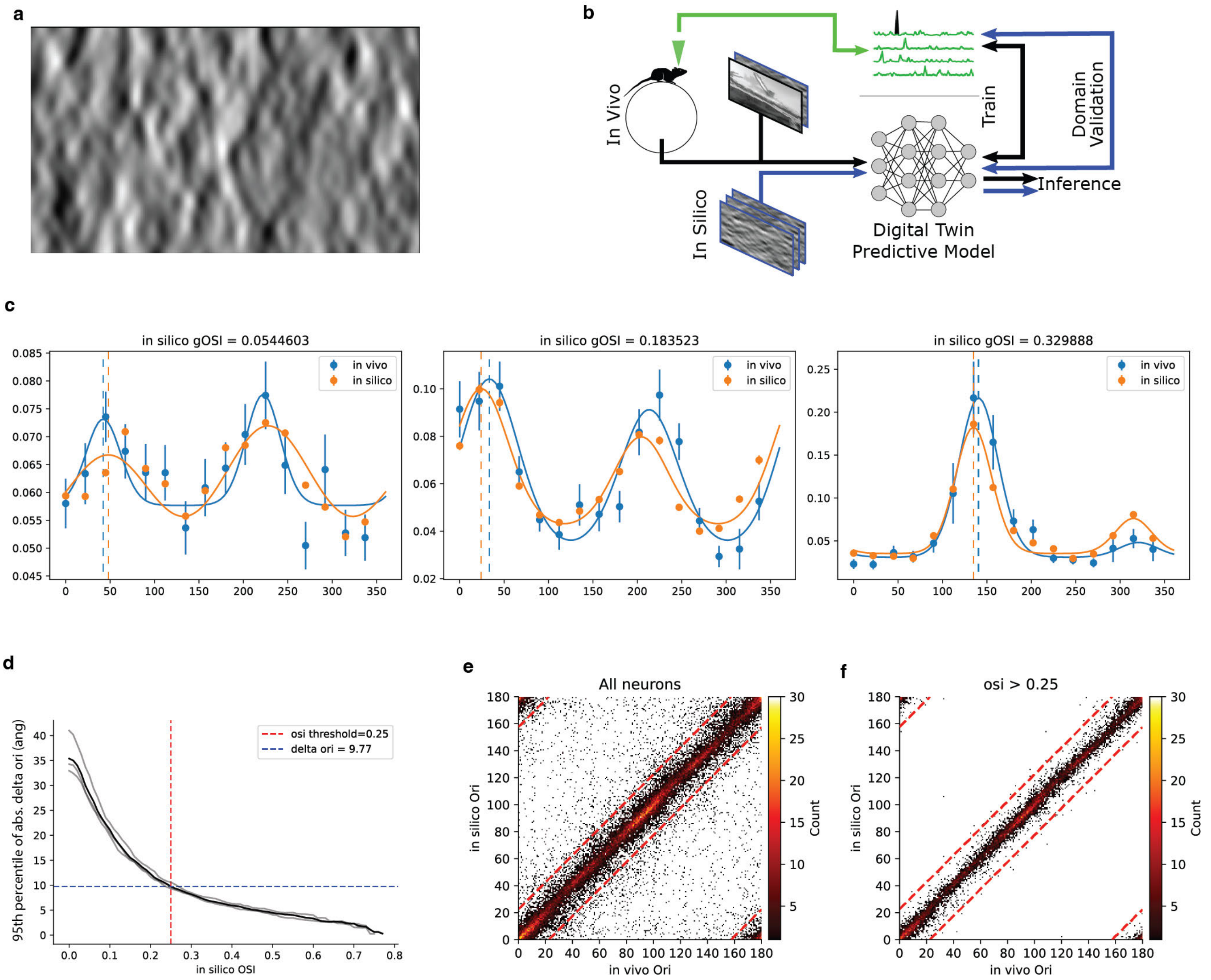

Supplemental Figure 7. In silico orientation tuning is consistent with in vivo orientation tuning a, Sample frame from global directional parametric stimulus ("Monet") used to characterize orientation and direction selectivity. Directional motion was orthogonal to orientation, and was tested at 22.5° intervals. **b**, Schematic of domain validation experimental design. In a single scan in a new animal, neuronal responses are collected in response to sufficient stimuli to both train the digital twin model (natural stimuli) and characterize orientation tuning (Monet) from *in vivo* responses. Later, *in silico* orientation tuning is extracted from model responses to parametric stimuli, and compared against *in vivo* orientation tuning for the same neurons. **c**, Comparison of *in silico* and *in vivo* mean responses per stimulus direction (mean $\pm$ SEM), fitted tuning curves (lines), and extracted preferred orientation (dotted lines) for three neurons. **d**, 95th percentile difference in preferred orientation between *in silico* and *in vivo* fitted responses as a function of gOSI threshold. Dotted lines correspond to gOSI > 0.25 threshold applied for all analyses and resulting 95th percentile difference in preferred orientation $\approx 9.77^\circ$ across all three animals imaged. Lines correspond to individual animals (gray) or cumulative across all animals (black). **e, f**, Two-dimensional histogram of *in silico* versus *in vivo* preferred orientation for all neurons across three animals (**e**) and only neurons with gOSI > 0.25 (**f**).

bioRxiv preprint doi: https://doi.org/10.1101/2023.03.13.531369; this version posted October 15, 2024. The copyright holder for this preprint (which was not certified by peer review) is the author/funder, who has granted bioRxiv a license to display the preprint in perpetuity. It is made available under aCC-BY 4.0 International license.

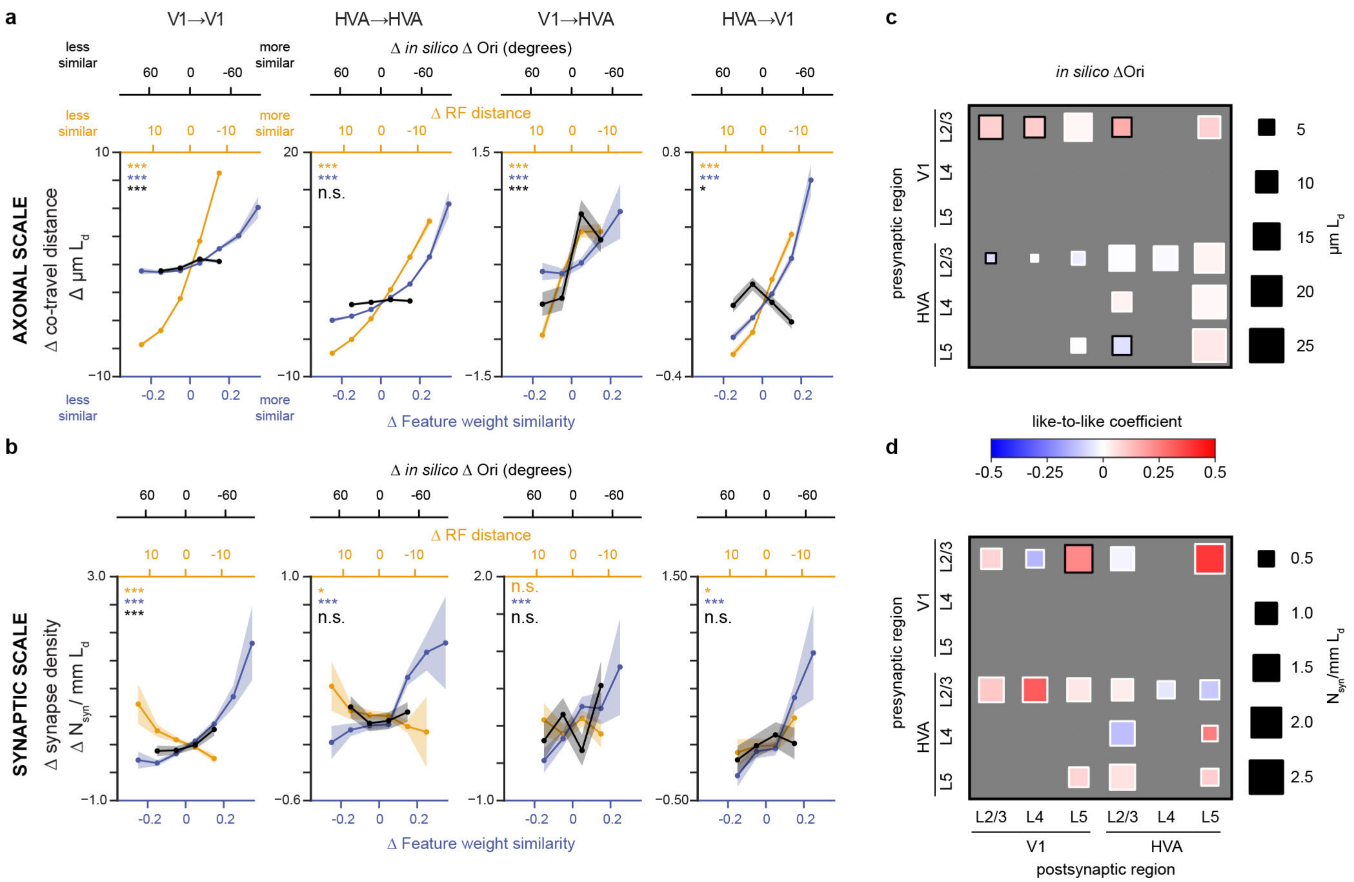

Supplemental Figure 8. Analysis repeated with *in silico* orientation preference. **a**, Difference in preferred orientation (Δ Ori) derived from *in silico* responses to parametric stimuli for tuned (gOSI > 0.25) neurons along with both feature weight similarity and receptive field center distance (reproduced from Fig 3) at axonal scale. **b**, same as in **a**, at synaptic scale. **c**, Area/ layer joint membership breakout as in Fig 4 for *in silico* Δ ori at axonal scale. **d**, As in **c** but at synaptic scale. All analyses are centered per presyn by accounting for the presyn mean (e.g. Δ feature weight similarity). For details, see Supplemental Tab. 13, 14, 17, 18, 21, 22, 31, 32,

bioRxiv preprint doi: https://doi.org/10.1101/2023.03.13.531369; this version posted October 15, 2024. The copyright holder for this preprint (which was not certified by peer review) is the author/funder, who has granted bioRxiv a license to display the preprint in perpetuity. It is made available under aCC-BY 4.0 International license.

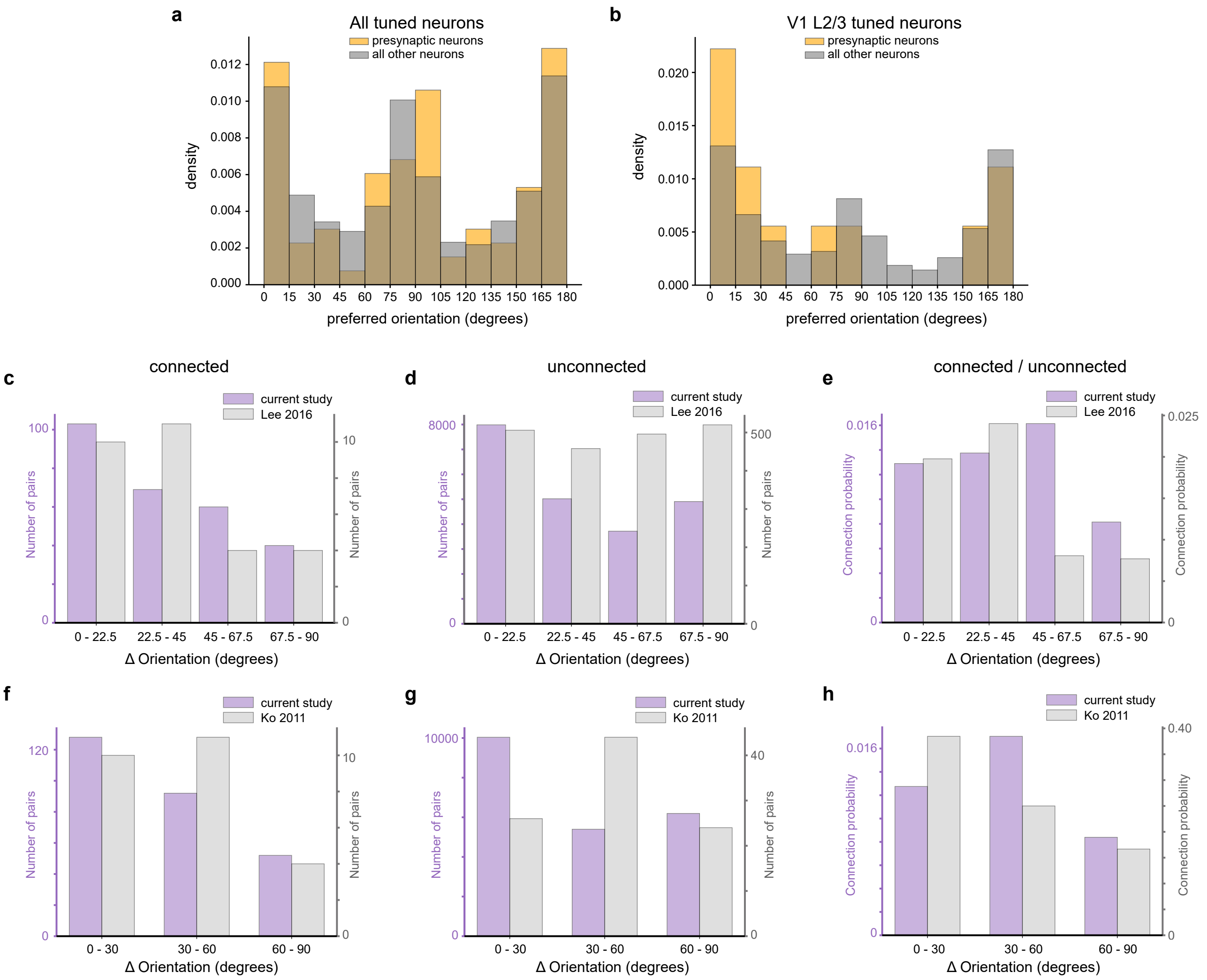

Supplemental Figure 9. Distribution of *in silico* orientation preference and comparison to previous literature. **a**, Distribution of orientation preference of tuned neurons (gOSI > 0.25) derived from *in silico* responses to parametric stimuli (see Methods). Note the cardinal bias in orientation preference distribution, in which orientation preference for 0 and 90 degree angles is overrepresented. Gold: presynaptic neurons, Gray: all other neurons. **b**, As in **a** but for tuned neurons in V1 L2/3. Difference in preferred orientation (Δ Orientation) for neurons in V1 L2/3 for connected pairs (**c, f**), unconnected pairs (**d, g**), and the ratio of connected / unconnected ("connection probability", **e, h**) for our study vs Lee et al. 2016 (**c-e**) and vs Ko et al. 2011 (**f-h**). The connected V1 L2/3 neurons in our study show a strong like-to-like effect, consistent with both Lee et al. 2016 and Ko et al. 2011 (**c, f**), however unlike Lee et al. 2016 and Ko et al. 2011, the unconnected neurons in our study also show a strong like-to-like effect (**d, g**) indicating that the like-to-like effect seen in connected pairs results from an orientation preference bias. This bias likely explains why we do not observe significant a like-to-like effect between V1 L2/3 neurons at axonal scale or synaptic scale in Supplemental. Fig 8, (i.e. when pairs are tested against region-matched controls).

bioRxiv preprint doi: https://doi.org/10.1101/2023.03.13.531369; this version posted October 15, 2024. The copyright holder for this preprint (which was not certified by peer review) is the author/funder, who has granted bioRxiv a license to display the preprint in perpetuity. It is made available under aCC-BY 4.0 International license.

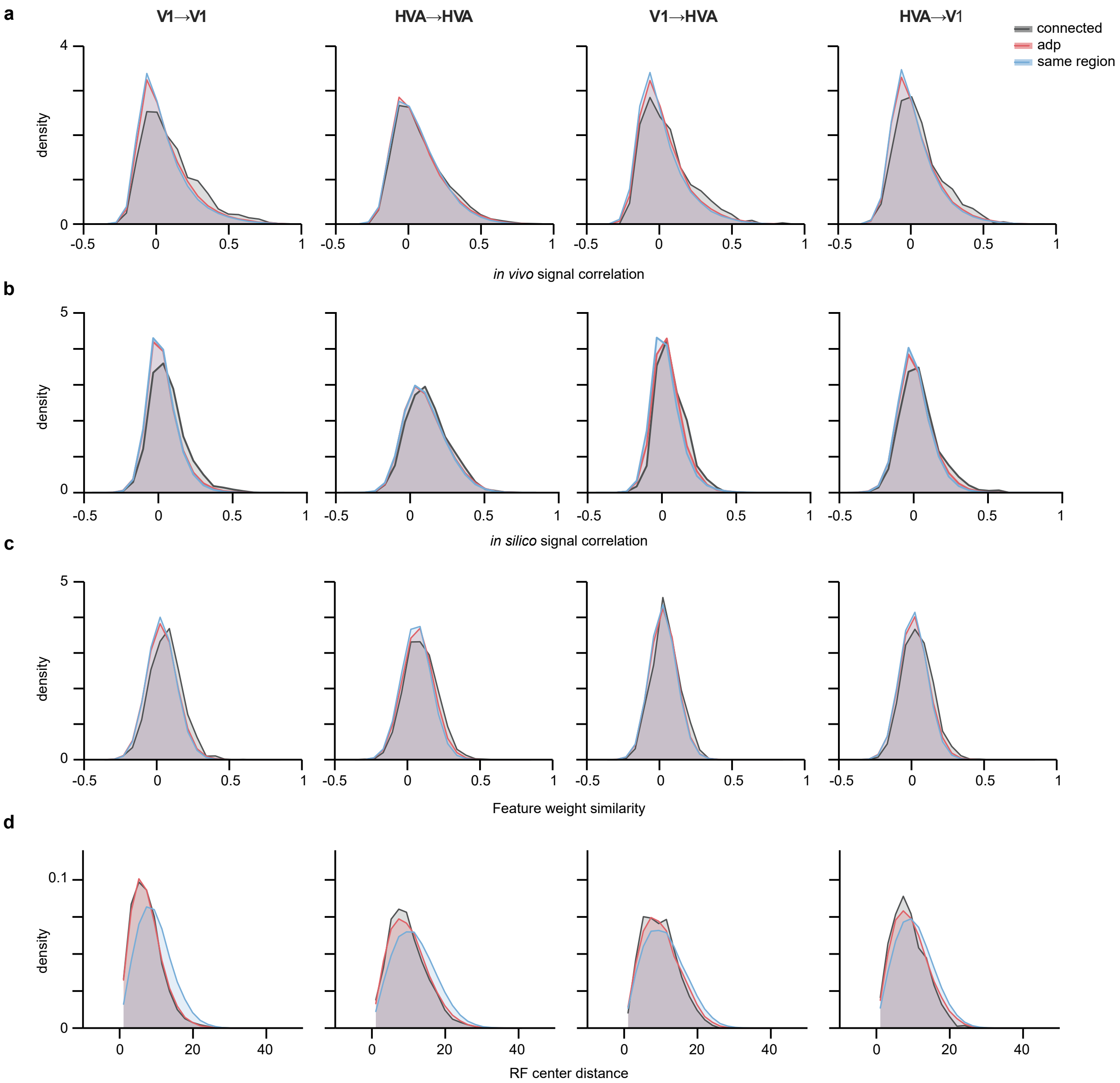

Supplemental Figure 10. Distribution of pairwise functional measurements. Density distribution of connected pairs (black), ADP control pairs (red) and same region control pairs (blue) for *in vivo* signal correlations (**a**), *in silico* signal correlations (**b**), feature weight similarity (**c**), and RF center distance (**d**) for all projection types.

bioRxiv preprint doi: https://doi.org/10.1101/2023.03.13.531369; this version posted October 15, 2024. The copyright holder for this preprint (which was not certified by peer review) is the author/funder, who has granted bioRxiv a license to display the preprint in perpetuity. It is made available under aCC-BY 4.0 International license.

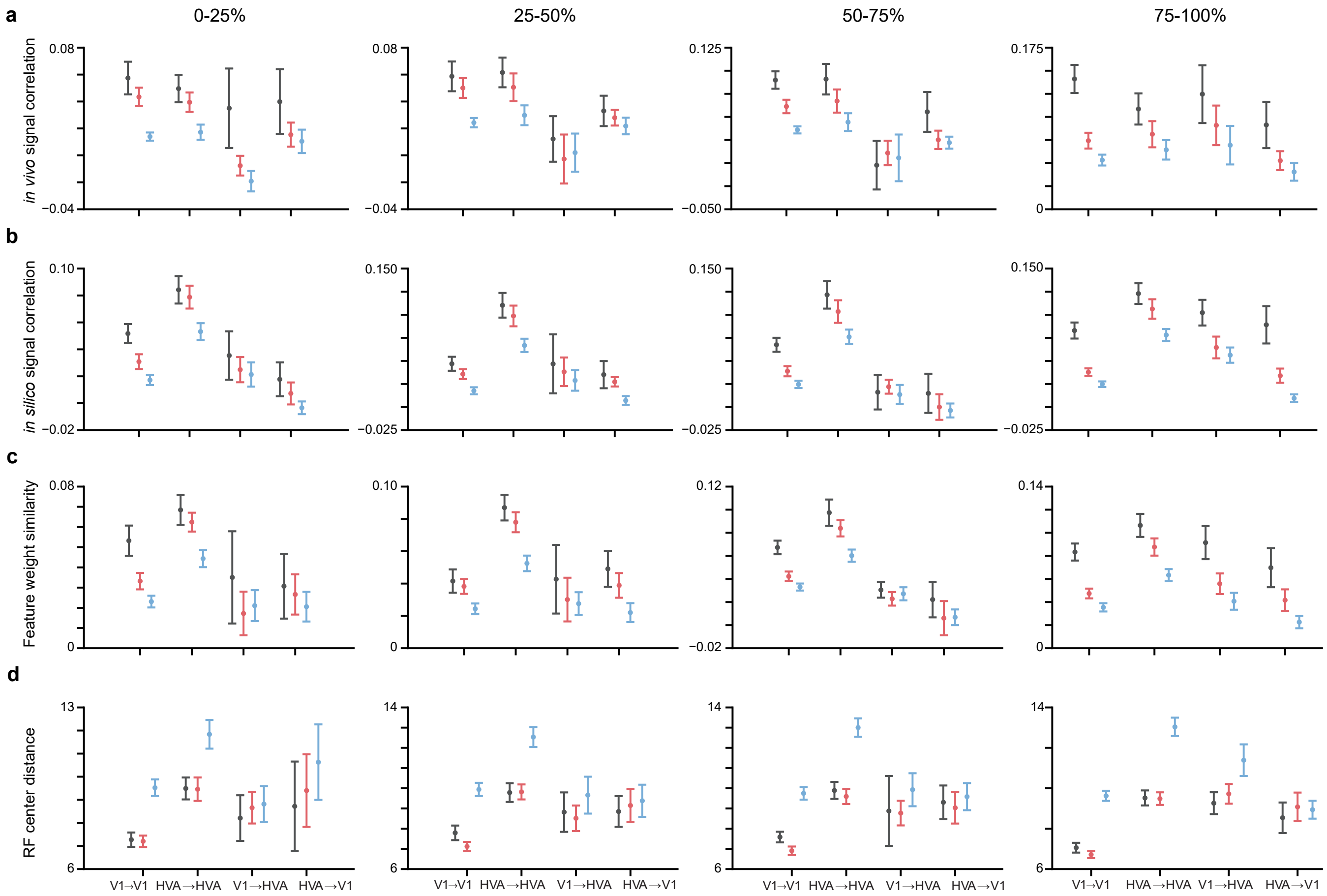

Supplemental Figure 11. Pairwise functional measurements across varying levels of model predictive performance. Mean of *in vivo* signal correlations (**a**), *in silico* signal correlations (**b**), feature weight similarity (**c**), and RF center distance (**d**) for all projection types across 4 quantiles of model predictive performance (CC_{abs}). All panels share a base filtering for visual responsiveness (CC_{max} > 0.4, 90% of neurons pass this threshold). Presynaptic neurons are filtered to CC_{abs} > 0.2 (4 did not pass this threshold).

bioRxiv preprint doi: https://doi.org/10.1101/2023.03.13.531369; this version posted October 15, 2024. The copyright holder for this preprint (which was not certified by peer review) is the author/funder, who has granted bioRxiv a license to display the preprint in perpetuity. It is made available under aCC-BY 4.0 International license.

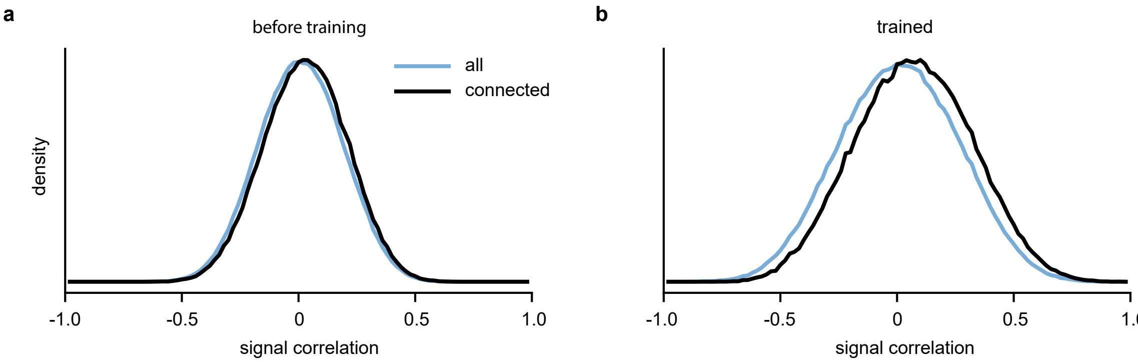

Supplemental Figure 12. Signal correlation distributions for connected neurons vs all neurons in the RNN before and after training.

a, Signal correlation distribution for connected neurons vs all neurons in the RNN before training. A neuron pair was classified as connected if the associated weight was in the top 35th percentile of all weights. **b**, Same as **a** except after training.

bioRxiv preprint doi: https://doi.org/10.1101/2023.03.13.531369; this version posted October 15, 2024. The copyright holder for this preprint (which was not certified by peer review) is the author/funder, who has granted bioRxiv a license to display the preprint in perpetuity. It is made available under aCC-BY 4.0 International license.

Supplemental Table 1. Proofread presynaptic neuron nucleus ID's, area, layer, and proofreading strategy. nucleus_id's are from CAVE table `nucleus_detection_v0`

index	nucleus_id	area	layer	proofreading strategy
1	189149	V1	L2/3	full cleaning and extension
2	222998	V1	L2/3	full cleaning and extension
3	223037	V1	L2/3	full cleaning and extension
4	224565	V1	L2/3	full cleaning and extension
5	225498	V1	L4	full cleaning and extension
6	230236	V1	L5	full cleaning and extension
7	236197	V1	L6	full cleaning and extension
8	255217	V1	L2/3	full cleaning and extension
9	256443	V1	L2/3	full cleaning and extension
10	256576	V1	L2/3	full cleaning and extension
11	258307	V1	L2/3	full cleaning and extension
12	259167	V1	L2/3	full cleaning and extension
13	262773	V1	L4	full cleaning and extension
14	264870	V1	L4	full cleaning and extension
15	269247	V1	L6	full cleaning and extension
16	269380	V1	L6	full cleaning and extension
17	271518	V1	L6	full cleaning and extension
18	292676	V1	L2/3	full cleaning and extension
19	292685	V1	L2/3	full cleaning and extension
20	292713	V1	L2/3	full cleaning and extension
21	294484	V1	L2/3	full cleaning and extension
22	294545	V1	L2/3	full cleaning and extension
23	294657	V1	L2/3	full cleaning and extension
24	294776	V1	L2/3	full cleaning and extension
25	294858	V1	L2/3	full cleaning and extension
26	294897	V1	L2/3	full cleaning and extension
27	296726	V1	L2/3	full cleaning and extension
28	300763	V1	L5	full cleaning and extension
29	301095	V1	L5	full cleaning and extension
30	301189	V1	L5	full cleaning and extension
31	327859	V1	L2/3	full cleaning and extension
32	330079	V1	L4	full cleaning and extension
33	330326	V1	L4	full cleaning and extension
34	331945	V1	L5	full cleaning and extension
35	332199	V1	L4	full cleaning and extension
36	335175	V1	L5	full cleaning and extension
37	460053	RL	L4	full cleaning and extension
38	460391	RL	L5	full cleaning and extension
39	487512	RL	L2/3	full cleaning and extension
40	489675	RL	L2/3	full cleaning and extension
41	493419	RL	L5	full cleaning and extension
42	493806	RL	L5	full cleaning and extension
43	493885	RL	L5	full cleaning and extension
44	493968	RL	L4	full cleaning and extension
45	516758	RL	L2/3	full cleaning and extension
46	517056	RL	L2/3	full cleaning and extension
47	518848	RL	L2/3	full cleaning and extension
48	518853	AL	L2/3	full cleaning and extension
49	518898	RL	L2/3	full cleaning and extension
50	520364	RL	L4	full cleaning and extension
51	522656	RL	L4	full cleaning and extension
52	524491	RL	L5	full cleaning and extension
53	525405	RL	L5	full cleaning and extension
54	525498	RL	L5	full cleaning and extension
55	525758	RL	L5	full cleaning and extension
56	553325	RL	L2/3	full cleaning and extension
57	554200	AL	L2/3	full cleaning and extension
58	554741	RL	L2/3	full cleaning and extension
59	554833	RL	L4	full cleaning and extension
60	554921	RL	L2/3	full cleaning and extension
61	556823	RL	L4	full cleaning and extension
62	557030	RL	L4	full cleaning and extension
63	557121	RL	L4	full cleaning and extension
64	558684	RL	L5	full cleaning and extension
65	558709	RL	L4	full cleaning and extension
66	559081	RL	L5	full cleaning and extension

Continued on next page

bioRxiv preprint doi: https://doi.org/10.1101/2023.03.13.531369; this version posted October 15, 2024. The copyright holder for this preprint (which was not certified by peer review) is the author/funder, who has granted bioRxiv a license to display the preprint in perpetuity. It is made available under aCC-BY 4.0 International license.

Supplemental Table 1. Proofread presynaptic neuron nucleus ID's, area, layer, and proofreading strategy

index	nucleus_id	area	layer	proofreading strategy
67	559381	RL	L5	full cleaning and extension
68	560109	RL	L5	full cleaning and extension
69	560217	RL	L5	full cleaning and extension
70	560530	RL	L5	full cleaning and extension
71	560732	RL	L5	full cleaning and extension
72	562808	RL	L5	full cleaning and extension
73	581967	AL	L2/3	full cleaning and extension
74	582056	AL	L2/3	full cleaning and extension
75	582129	AL	L2/3	full cleaning and extension
76	582210	AL	L2/3	full cleaning and extension
77	583848	AL	L2/3	full cleaning and extension
78	583961	RL	L2/3	full cleaning and extension
79	585723	RL	L4	full cleaning and extension
80	587426	AL	L4	full cleaning and extension
81	588839	RL	L5	full cleaning and extension
82	588983	AL	L5	full cleaning and extension
83	610498	AL	L2/3	full cleaning and extension
84	616159	AL	L5	full cleaning and extension
85	516621	RL	L2/3	full cleaning and partial axonal extension
86	516988	RL	L2/3	full cleaning and partial axonal extension
87	517993	RL	L2/3	full cleaning and partial axonal extension
88	518004	RL	L2/3	full cleaning and partial axonal extension
89	518134	RL	L2/3	full cleaning and partial axonal extension
90	518224	RL	L2/3	full cleaning and partial axonal extension
91	518312	RL	L2/3	full cleaning and partial axonal extension
92	518623	RL	L2/3	full cleaning and partial axonal extension
93	518632	RL	L2/3	full cleaning and partial axonal extension
94	519746	RL	L2/3	full cleaning and partial axonal extension
95	520027	RL	L4	full cleaning and partial axonal extension
96	520182	RL	L2/3	full cleaning and partial axonal extension
97	551802	RL	L2/3	full cleaning and partial axonal extension
98	553216	RL	L2/3	full cleaning and partial axonal extension
99	553283	RL	L2/3	full cleaning and partial axonal extension
100	553321	RL	L2/3	full cleaning and partial axonal extension
101	553339	RL	L2/3	full cleaning and partial axonal extension
102	553360	RL	L2/3	full cleaning and partial axonal extension
103	553469	RL	L2/3	full cleaning and partial axonal extension
104	553556	RL	L2/3	full cleaning and partial axonal extension
105	553585	RL	L2/3	full cleaning and partial axonal extension
106	553589	RL	L2/3	full cleaning and partial axonal extension
107	554734	RL	L2/3	full cleaning and partial axonal extension
108	554775	RL	L2/3	full cleaning and partial axonal extension
109	554891	RL	L2/3	full cleaning and partial axonal extension
110	554900	RL	L2/3	full cleaning and partial axonal extension
111	555010	RL	L2/3	full cleaning and partial axonal extension
112	580774	AL	L2/3	full cleaning and partial axonal extension
113	580826	AL	L2/3	full cleaning and partial axonal extension
114	580905	AL	L2/3	full cleaning and partial axonal extension
115	580948	RL	L2/3	full cleaning and partial axonal extension
116	580988	AL	L2/3	full cleaning and partial axonal extension
117	581988	AL	L2/3	full cleaning and partial axonal extension
118	581998	AL	L2/3	full cleaning and partial axonal extension
119	582011	AL	L2/3	full cleaning and partial axonal extension
120	582091	AL	L2/3	full cleaning and partial axonal extension
121	582294	AL	L2/3	full cleaning and partial axonal extension
122	582313	RL	L2/3	full cleaning and partial axonal extension
123	582353	AL	L2/3	full cleaning and partial axonal extension
124	582388	RL	L2/3	full cleaning and partial axonal extension
125	582390	RL	L2/3	full cleaning and partial axonal extension
126	582409	RL	L2/3	full cleaning and partial axonal extension
127	582412	RL	L2/3	full cleaning and partial axonal extension
128	582414	RL	L2/3	full cleaning and partial axonal extension
129	582444	RL	L2/3	full cleaning and partial axonal extension
130	582468	RL	L2/3	full cleaning and partial axonal extension
131	582471	RL	L2/3	full cleaning and partial axonal extension
132	583659	AL	L2/3	full cleaning and partial axonal extension
133	583739	AL	L2/3	full cleaning and partial axonal extension
134	583741	AL	L2/3	full cleaning and partial axonal extension

Continued on next page

bioRxiv preprint doi: https://doi.org/10.1101/2023.03.13.531369; this version posted October 15, 2024. The copyright holder for this preprint (which was not certified by peer review) is the author/funder, who has granted bioRxiv a license to display the preprint in perpetuity. It is made available under aCC-BY 4.0 International license.

Supplemental Table 1. Proofread presynaptic neuron nucleus ID's, area, layer, and proofreading strategy

index	nucleus_id	area	layer	proofreading strategy
135	583792	AL	L2/3	full cleaning and partial axonal extension
136	583891	RL	L2/3	full cleaning and partial axonal extension
137	584004	RL	L2/3	full cleaning and partial axonal extension
138	608166	AL	L2/3	full cleaning and partial axonal extension
139	608213	AL	L2/3	full cleaning and partial axonal extension
140	610396	AL	L2/3	full cleaning and partial axonal extension
141	610403	AL	L2/3	full cleaning and partial axonal extension
142	610434	AL	L2/3	full cleaning and partial axonal extension
143	610535	AL	L2/3	full cleaning and partial axonal extension
144	610607	AL	L2/3	full cleaning and partial axonal extension
145	610615	AL	L2/3	full cleaning and partial axonal extension
146	612143	AL	L2/3	full cleaning and partial axonal extension
147	612266	AL	L2/3	full cleaning and partial axonal extension
148	612352	AL	L2/3	full cleaning and partial axonal extension

bioRxiv preprint doi: https://doi.org/10.1101/2023.03.13.531369; this version posted October 15, 2024. The copyright holder for this preprint (which was not certified by peer review) is the author/funder, who has granted bioRxiv a license to display the preprint in perpetuity. It is made available under aCC-BY 4.0 International license.

Supplemental Table 2. Pairwise comparison of the presynaptic mean in silico signal correlation between different neuron pair populations. For each comparison, a pairwise t-test was performed to test the null hypothesis that for each presynaptic neuron, the mean in silico signal correlation is the same between two postsynaptic populations. adjusted p-value is the adjusted p-value through the BH multicomparison correction procedure.

Comparison	Projection type	Mean pairwise difference	p-value	adjusted p-value	t statistic	n
ADP vs Same region	HVA->HVA	0.015	5.30e-05	7.96e-05	4.405	53
ADP vs Same region	HVA->V1	0.007	1.14e-02	1.14e-02	2.661	39
ADP vs Same region	V1->HVA	0.011	3.12e-03	3.41e-03	3.475	17
ADP vs Same region	V1->V1	0.009	3.18e-05	5.45e-05	4.793	35
Connected vs ADP	HVA->HVA	0.026	3.58e-08	2.15e-07	6.460	53
Connected vs ADP	HVA->V1	0.029	7.85e-06	1.57e-05	5.168	39
Connected vs ADP	V1->HVA	0.023	1.25e-03	1.50e-03	3.908	17
Connected vs ADP	V1->V1	0.030	7.33e-06	1.57e-05	5.285	35
Connected vs Same region	HVA->HVA	0.042	3.37e-10	4.04e-09	7.733	53
Connected vs Same region	HVA->V1	0.036	5.77e-06	1.57e-05	5.266	39
Connected vs Same region	V1->HVA	0.034	9.21e-05	1.23e-04	5.175	17
Connected vs Same region	V1->V1	0.039	4.05e-07	1.62e-06	6.253	35

bioRxiv preprint doi: https://doi.org/10.1101/2023.03.13.531369; this version posted October 15, 2024. The copyright holder for this preprint (which was not certified by peer review) is the author/funder, who has granted bioRxiv a license to display the preprint in perpetuity. It is made available under aCC-BY 4.0 International license.

Supplemental Table 3. Number of neurons and neuron pairs invovled in the visualization of the correlation between in silico signal correlation and L_d / neuron pair (synapses excluded) in different projection types across brain areas.

Projection type	Δ in silico signal correlation bin	# of presynaptic neurons	# of postsynaptic neurons	# of ADP control neurons	# of same region control neurons	# of pre-post pairs	# of pre-ADP pairs	# of pre-'same region' pairs	# of synapses	total L_d (mm)
$V1 \to V1$	-0.30 - -0.20	27	0	427	27	0	570	1518	0	14.618620
$V1 \to V1$	-0.20 - -0.10	36	0	3624	36	0	8358	22716	0	235.352922
$V1 \to V1$	-0.10 - -0.00	36	0	5939	36	0	31843	82271	0	943.324036
$V1 \to V1$	-0.00 - 0.10	36	0	5575	36	0	22037	53619	0	704.008419
$V1 \to V1$	0.10 - 0.20	36	0	3884	36	0	8136	18497	0	268.080820
$V1 \to V1$	0.20 - 0.30	36	0	1938	36	0	2662	5310	0	86.811831
$V1 \to V1$	0.30 - 0.40	36	0	754	36	0	862	1373	0	29.303881
$V1 \to V1$	0.40 - 0.50	34	0	245	27	0	256	350	0	8.436686
$HVA \to HVA$	-0.30 - -0.20	100	0	737	98	0	2642	9566	0	76.177507
$HVA \to HVA$	-0.20 - -0.10	102	0	2207	102	0	13596	36699	0	417.557033
$HVA \to HVA$	-0.10 - -0.00	102	0	2593	102	0	29335	70079	0	994.561220
$HVA \to HVA$	-0.00 - 0.10	102	0	2549	102	0	24611	58614	0	853.487712
$HVA \to HVA$	0.10 - 0.20	102	0	2264	102	0	13152	29881	0	484.795597
$HVA \to HVA$	0.20 - 0.30	102	0	1677	102	0	5266	10534	0	203.187307
$HVA \to HVA$	0.30 - 0.40	102	0	828	102	0	1469	2700	0	57.001171
$V1 \to HVA$	-0.20 - -0.10	29	0	958	29	0	1430	7995	0	30.452029
$V1 \to HVA$	-0.10 - -0.00	29	0	2203	29	0	5725	32680	0	141.454788
$V1 \to HVA$	-0.00 - 0.10	29	0	2027	29	0	4825	23561	0	123.475999
$V1 \to HVA$	0.10 - 0.20	29	0	1038	29	0	1541	7314	0	38.443692
$V1 \to HVA$	0.20 - 0.30	29	0	348	29	0	398	1794	0	9.596663
$HVA \to V1$	-0.30 - -0.20	87	0	450	87	0	731	8861	0	13.161841
$HVA \to V1$	-0.20 - -0.10	92	0	2834	92	0	6850	88153	0	123.501498
$HVA \to V1$	-0.10 - -0.00	92	0	5315	92	0	17932	245585	0	345.186645
$HVA \to V1$	-0.00 - 0.10	92	0	4582	92	0	13701	180600	0	279.289998
$HVA \to V1$	0.10 - 0.20	92	0	2753	92	0	5823	70851	0	119.710286
$HVA \to V1$	0.20 - 0.30	92	0	1345	92	0	2135	21317	0	44.995031
$HVA \to V1$	0.30 - 0.40	92	0	479	92	0	616	5487	0	13.275195

bioRxiv preprint doi: https://doi.org/10.1101/2023.03.13.531369; this version posted October 15, 2024. The copyright holder for this preprint (which was not certified by peer review) is the author/funder, who has granted bioRxiv a license to display the preprint in perpetuity. It is made available under aCC-BY 4.0 International license.

Supplemental Table 4. Number of neurons and neuron pairs invovled in the visualization of the correlation between in silico signal correlation and $N_{syn}/mm\ L_d$ in different projection types across brain areas.

Projection type	Δ in silico signal correlation bin	# of presynaptic neurons	# of postsynaptic neurons	# of ADP control neurons	# of same region control neurons	# of pre-post pairs	# of pre-ADP pairs	# of pre-'same region' pairs	# of synapses	totalL_d (mm)
$V1 \rightarrow V1$	-0.30 - -0.20	25	14	519	0	14	723	0	15	18.799029
$V1 \rightarrow V1$	-0.20 - -0.10	36	205	3928	0	214	9922	0	232	283.947056
$V1 \rightarrow V1$	-0.10 - -0.00	36	736	5943	0	850	33384	0	945	1018.234754
$V1 \rightarrow V1$	-0.00 - 0.10	36	664	5534	0	767	21646	0	881	721.429779
$V1 \rightarrow V1$	0.10 - 0.20	36	305	3713	0	337	7791	0	392	267.558394
$V1 \rightarrow V1$	0.20 - 0.30	36	135	1817	0	145	2574	0	182	86.912818
$V1 \rightarrow V1$	0.30 - 0.40	36	46	693	0	47	823	0	54	29.728849
$V1 \rightarrow V1$	0.40 - 0.50	29	27	220	0	27	256	0	32	8.926083
$HVA \rightarrow HVA$	-0.30 - -0.20	92	49	830	0	52	3241	0	59	94.472855
$HVA \rightarrow HVA$	-0.20 - -0.10	99	285	2252	0	328	14928	0	359	481.976078
$HVA \rightarrow HVA$	-0.10 - -0.00	99	624	2596	0	778	30495	0	836	1077.808884
$HVA \rightarrow HVA$	-0.00 - 0.10	99	584	2538	0	755	24558	0	841	893.638912
$HVA \rightarrow HVA$	0.10 - 0.20	99	324	2231	0	392	12399	0	440	472.381883
$HVA \rightarrow HVA$	0.20 - 0.30	99	160	1590	0	169	4537	0	194	180.483568
$HVA \rightarrow HVA$	0.30 - 0.40	95	52	714	0	54	1168	0	59	49.267130
$V1 \rightarrow HVA$	-0.20 - -0.10	28	38	1053	0	38	1684	0	39	37.691653
$V1 \rightarrow HVA$	-0.10 - -0.00	29	187	2231	0	200	6180	0	226	156.738585
$V1 \rightarrow HVA$	-0.00 - 0.10	29	159	1975	0	169	4723	0	194	126.623281
$V1 \rightarrow HVA$	0.10 - 0.20	28	76	938	0	80	1468	0	94	38.089134
$V1 \rightarrow HVA$	0.20 - 0.30	26	24	301	0	24	368	0	26	9.252531
$HVA \rightarrow V1$	-0.30 - -0.20	69	13	531	0	13	879	0	13	15.455242
$HVA \rightarrow V1$	-0.20 - -0.10	90	133	3029	0	138	7559	0	150	142.347339
$HVA \rightarrow V1$	-0.10 - -0.00	90	364	5363	0	385	18588	0	425	370.298550
$HVA \rightarrow V1$	-0.00 - 0.10	90	327	4534	0	349	13530	0	370	281.465665
$HVA \rightarrow V1$	0.10 - 0.20	90	142	2648	0	146	5607	0	168	118.052918
$HVA \rightarrow V1$	0.20 - 0.30	90	67	1257	0	70	2008	0	77	43.367913
$HVA \rightarrow V1$	0.30 - 0.40	76	25	423	0	27	559	0	38	12.949679

bioRxiv preprint doi: https://doi.org/10.1101/2023.03.13.531369; this version posted October 15, 2024. The copyright holder for this preprint (which was not certified by peer review) is the author/funder, who has granted bioRxiv a license to display the preprint in perpetuity. It is made available under aCC-BY 4.0 International license.

Supplemental Table 5. Estimated marginal means of linear trends for the effect of in silico signal correlation on L_d / neuron pair (synapses excluded) in different projection types across brain areas. z and p-value are the z statistics and p-value of the marginal mean linear trends estimated from the fitted GLMMs. adjusted p-value is the adjusted p value through the BH multicomparison correction procedure.

Projection type	Coefficient	adjusted p-value	p-value	# of presynaptic neurons	# of postsynaptic neurons	# of ADP control neurons	# of same region control neurons	# of synapses	# of pre-post pairs	# of pre-ADP pairs	# of pre-'same region' pairs
$V1 \rightarrow V1$	1.125	5.18e-160	2.59e-160	36	0	6237	36	0	0	74829	185807
$HVA \rightarrow HVA$	1.109	4.24e-278	1.06e-278	99	0	2635	99	0	0	89611	212583
$V1 \rightarrow HVA$	1.101	5.36e-25	5.36e-25	29	0	2525	29	0	0	14126	74633
$HVA \rightarrow V1$	0.872	1.48e-82	1.11e-82	90	0	6148	90	0	0	47811	608388

bioRxiv preprint doi: https://doi.org/10.1101/2023.03.13.531369; this version posted October 15, 2024. The copyright holder for this preprint (which was not certified by peer review) is the author/funder, who has granted bioRxiv a license to display the preprint in perpetuity. It is made available under aCC-BY 4.0 International license.

Supplemental Table 6. Estimated marginal means of linear trends for the effect of in silico signal correlation on $N_{syn}/mm\ L_d$ in different projection types across brain areas. z and p-value are the z statistics and p-value of the marginal mean linear trends estimated from the fitted GLMMs. adjusted p-value is the adjusted p value through the BH multicomparison correction procedure.

Projection type	Coefficient	adjusted p-value	p-value	# of presynaptic neurons	# of postsynaptic neurons	# of ADP control neurons	# of same region control neurons	# of synapses	# of pre-post pairs	# of pre-ADP pairs	# of pre-'same region' pairs
$V1 \rightarrow V1$	2.297	9.17e-50	2.29e-50	36	1719	6237	0	2744	2411	77240	0
$HVA \rightarrow HVA$	1.043	3.84e-12	2.88e-12	99	1396	2635	0	2803	2543	92154	0
$V1 \rightarrow HVA$	1.985	8.57e-07	8.57e-07	29	448	2525	0	584	515	14641	0
$HVA \rightarrow V1$	1.603	2.59e-12	1.29e-12	90	974	6148	0	1255	1139	48950	0

bioRxiv preprint doi: https://doi.org/10.1101/2023.03.13.531369; this version posted October 15, 2024. The copyright holder for this preprint (which was not certified by peer review) is the author/funder, who has granted bioRxiv a license to display the preprint in perpetuity. It is made available under aCC-BY 4.0 International license.

Supplemental Table 7. Number of neurons and neuron pairs invovled in the visualization of the correlation between in vivo signal correlation and L_d / neuron pair (synapses excluded) in different projection types across brain areas.

Projection type	Δ in vivo signal correlation bin	# of presynaptic neurons	# of postsynaptic neurons	# of ADP control neurons	# of same region control neurons	# of pre-post pairs	# of pre-ADP pairs	# of pre-'same region' pairs	# of synapses	total L_d (mm)
$V1 \rightarrow V1$	-0.30 - -0.20	36	0	1552	36	0	2656	9132	0	69.390757
$V1 \rightarrow V1$	-0.20 - -0.10	36	0	5565	36	0	19022	54877	0	543.353655
$V1 \rightarrow V1$	-0.10 - -0.00	36	0	6114	36	0	23976	60410	0	728.386124
$V1 \rightarrow V1$	-0.00 - 0.10	36	0	5449	36	0	15642	37077	0	501.565276
$V1 \rightarrow V1$	0.10 - 0.20	36	0	4178	36	0	9062	20187	0	296.744655
$V1 \rightarrow V1$	0.20 - 0.30	36	0	2902	36	0	4947	10613	0	158.206795
$V1 \rightarrow V1$	0.30 - 0.40	36	0	1800	36	0	2584	5761	0	82.535045
$V1 \rightarrow V1$	0.40 - 0.50	36	0	1091	36	0	1399	3143	0	45.476341
$V1 \rightarrow V1$	0.50 - 0.60	35	0	625	35	0	768	1571	0	24.006232
$V1 \rightarrow V1$	0.60 - 0.70	31	0	317	29	0	384	747	0	12.507211
$HVA \rightarrow HVA$	-0.40 - -0.30	65	0	225	64	0	446	1904	0	11.872237
$HVA \rightarrow HVA$	-0.30 - -0.20	104	0	1212	104	0	4695	15354	0	141.825146
$HVA \rightarrow HVA$	-0.20 - -0.10	106	0	2805	106	0	20166	54570	0	653.240105
$HVA \rightarrow HVA$	-0.10 - -0.00	106	0	2972	106	0	32117	79749	0	1072.653987
$HVA \rightarrow HVA$	-0.00 - 0.10	106	0	2910	106	0	22291	56261	0	763.312753
$HVA \rightarrow HVA$	0.10 - 0.20	106	0	2621	106	0	12457	31246	0	436.743130
$HVA \rightarrow HVA$	0.20 - 0.30	106	0	2107	106	0	6603	15249	0	235.733457
$HVA \rightarrow HVA$	0.30 - 0.40	106	0	1408	106	0	3156	6797	0	120.009172
$HVA \rightarrow HVA$	0.40 - 0.50	106	0	842	106	0	1479	2788	0	57.110029
$HVA \rightarrow HVA$	0.50 - 0.60	99	0	417	95	0	575	977	0	24.809982
$V1 \rightarrow HVA$	-0.30 - -0.20	29	0	381	29	0	472	3237	0	11.502273
$V1 \rightarrow HVA$	-0.20 - -0.10	29	0	1740	29	0	3188	17931	0	77.885536
$V1 \rightarrow HVA$	-0.10 - -0.00	29	0	2299	29	0	5273	29111	0	131.740088
$V1 \rightarrow HVA$	-0.00 - 0.10	29	0	1896	29	0	3584	17887	0	87.290731
$V1 \rightarrow HVA$	0.10 - 0.20	29	0	1232	29	0	1850	8793	0	46.915050
$V1 \rightarrow HVA$	0.20 - 0.30	29	0	706	29	0	915	4306	0	23.142961
$V1 \rightarrow HVA$	0.30 - 0.40	29	0	376	29	0	432	2088	0	9.855496
$V1 \rightarrow HVA$	0.40 - 0.50	29	0	212	29	0	253	1004	0	6.288603
$HVA \rightarrow V1$	-0.30 - -0.20	92	0	1188	92	0	1960	24054	0	34.934123
$HVA \rightarrow V1$	-0.20 - -0.10	94	0	4640	94	0	12391	153177	0	234.769489
$HVA \rightarrow V1$	-0.10 - -0.00	94	0	5673	94	0	17333	227262	0	341.230850
$HVA \rightarrow V1$	-0.00 - 0.10	94	0	4812	94	0	11544	145012	0	230.704006
$HVA \rightarrow V1$	0.10 - 0.20	94	0	3291	94	0	6244	75588	0	127.891275
$HVA \rightarrow V1$	0.20 - 0.30	94	0	1974	94	0	3115	37057	0	64.396003
$HVA \rightarrow V1$	0.30 - 0.40	94	0	1167	94	0	1611	17422	0	31.785912
$HVA \rightarrow V1$	0.40 - 0.50	94	0	578	94	0	699	7804	0	14.966269

bioRxiv preprint doi: https://doi.org/10.1101/2023.03.13.531369; this version posted October 15, 2024. The copyright holder for this preprint (which was not certified by peer review) is the author/funder, who has granted bioRxiv a license to display the preprint in perpetuity. It is made available under aCC-BY 4.0 International license.

Supplemental Table 8. Number of neurons and neuron pairs invovled in the visualization of the correlation between in vivo signal correlation and $N_{syn}/mm\ L_d$ in different projection types across brain areas.

Projection type	Δ in vivo signal correlation bin	# of presynaptic neurons	# of postsynaptic neurons	# of ADP control neurons	# of same region control neurons	# of pre-post pairs	# of pre-ADP pairs	# of pre-'same region' pairs	# of synapses	total L_d (mm)
$V1 \rightarrow V1$	-0.30 - -0.20	34	101	2202	0	104	4393	0	116	120.196380
$V1 \rightarrow V1$	-0.20 - -0.10	36	483	5747	0	520	20983	0	569	614.701246
$V1 \rightarrow V1$	-0.10 - -0.00	36	588	6111	0	657	23822	0	734	749.965351
$V1 \rightarrow V1$	-0.00 - 0.10	36	477	5353	0	525	15389	0	582	510.116138
$V1 \rightarrow V1$	0.10 - 0.20	36	299	4038	0	327	8806	0	382	301.521896
$V1 \rightarrow V1$	0.20 - 0.30	36	205	2774	0	223	4735	0	263	160.163966
$V1 \rightarrow V1$	0.30 - 0.40	36	111	1665	0	112	2461	0	135	82.914655
$V1 \rightarrow V1$	0.40 - 0.50	36	64	1006	0	66	1325	0	79	43.172834
$V1 \rightarrow V1$	0.50 - 0.60	35	29	571	0	32	723	0	42	24.178848
$V1 \rightarrow V1$	0.60 - 0.70	30	21	286	0	22	367	0	33	12.753262
$HVA \rightarrow HVA$	-0.40 - -0.30	50	13	332	0	13	742	0	15	20.548477
$HVA \rightarrow HVA$	-0.30 - -0.20	99	87	1393	0	93	5788	0	103	180.512305
$HVA \rightarrow HVA$	-0.20 - -0.10	105	406	2837	0	471	21472	0	509	716.812856
$HVA \rightarrow HVA$	-0.10 - -0.00	105	688	2969	0	872	32905	0	947	1142.705436
$HVA \rightarrow HVA$	-0.00 - 0.10	105	552	2911	0	661	22349	0	715	794.109314
$HVA \rightarrow HVA$	0.10 - 0.20	105	304	2599	0	344	12079	0	391	435.790667
$HVA \rightarrow HVA$	0.20 - 0.30	105	173	2058	0	188	6251	0	218	234.891093
$HVA \rightarrow HVA$	0.30 - 0.40	105	104	1345	0	109	2938	0	126	117.834260
$HVA \rightarrow HVA$	0.40 - 0.50	101	46	781	0	48	1315	0	51	51.536756
$HVA \rightarrow HVA$	0.50 - 0.60	83	20	376	0	20	519	0	24	23.481751
$V1 \rightarrow HVA$	-0.30 - -0.20	24	18	450	0	18	593	0	21	14.369431
$V1 \rightarrow HVA$	-0.20 - -0.10	29	117	1875	0	123	3741	0	140	95.546797
$V1 \rightarrow HVA$	-0.10 - -0.00	29	168	2317	0	175	5532	0	196	141.870319
$V1 \rightarrow HVA$	-0.00 - 0.10	29	121	1822	0	125	3417	0	140	86.935079
$V1 \rightarrow HVA$	0.10 - 0.20	29	69	1139	0	71	1715	0	78	44.698868
$V1 \rightarrow HVA$	0.20 - 0.30	26	44	661	0	44	889	0	50	23.830929
$V1 \rightarrow HVA$	0.30 - 0.40	27	25	358	0	26	449	0	31	10.845062
$V1 \rightarrow HVA$	0.40 - 0.50	25	11	195	0	11	237	0	13	6.017091
$HVA \rightarrow V1$	-0.30 - -0.20	85	46	1263	0	46	2180	0	50	40.028150
$HVA \rightarrow V1$	-0.20 - -0.10	93	211	4711	0	220	12816	0	242	248.419138
$HVA \rightarrow V1$	-0.10 - -0.00	93	375	5690	0	400	17857	0	434	361.524099
$HVA \rightarrow V1$	-0.00 - 0.10	93	291	4804	0	305	11709	0	333	241.200378
$HVA \rightarrow V1$	0.10 - 0.20	93	159	3250	0	164	6214	0	181	129.824307
$HVA \rightarrow V1$	0.20 - 0.30	93	96	1933	0	98	3084	0	116	65.574084
$HVA \rightarrow V1$	0.30 - 0.40	91	44	1119	0	45	1531	0	49	31.773139
$HVA \rightarrow V1$	0.40 - 0.50	82	30	549	0	30	689	0	33	15.452123

bioRxiv preprint doi: https://doi.org/10.1101/2023.03.13.531369; this version posted October 15, 2024. The copyright holder for this preprint (which was not certified by peer review) is the author/funder, who has granted bioRxiv a license to display the preprint in perpetuity. It is made available under aCC-BY 4.0 International license.

Supplemental Table 9. Estimated marginal means of linear trends for the effect of in vivo signal correlation on L_d / neuron pair (synapses excluded) in different projection types across brain areas. z and p-value are the z statistics and p-value of the marginal mean linear trends estimated from the fitted GLMMs. adjusted p-value is the adjusted p value through the BH multicomparison correction procedure.

Projection type	Coefficient	adjusted p-value	p-value	# of presynaptic neurons	# of postsynaptic neurons	# of ADP control neurons	# of same region control neurons	# of synapses	# of pre-post pairs	# of pre-ADP pairs	# of pre-'same region' pairs
$V1 \rightarrow V1$	0.779	1.96e-210	4.89e-211	36	0	6807	36	0	0	80703	204150
$HVA \rightarrow HVA$	0.698	3.51e-198	1.75e-198	105	0	3010	105	0	0	103839	262309
$V1 \rightarrow HVA$	0.624	1.60e-23	1.60e-23	29	0	2887	29	0	0	16164	85137
$HVA \rightarrow V1$	0.428	5.69e-43	4.27e-43	93	0	6720	93	0	0	55313	685610

bioRxiv preprint doi: https://doi.org/10.1101/2023.03.13.531369; this version posted October 15, 2024. The copyright holder for this preprint (which was not certified by peer review) is the author/funder, who has granted bioRxiv a license to display the preprint in perpetuity. It is made available under aCC-BY 4.0 International license.

Supplemental Table 10. Estimated marginal means of linear trends for the effect of in vivo signal correlation on $N_{syn}/mm\ L_d$ in different projection types across brain areas. z and p-value are the z statistics and p-value of the marginal mean linear trends estimated from the fitted GLMMs. adjusted p-value is the adjusted p value through the BH multicomparison correction procedure.

Projection type	Coefficient	adjusted p-value	p-value	# of presynaptic neurons	# of postsynaptic neurons	# of ADP control neurons	# of same region control neurons	# of synapses	# of pre-post pairs	# of pre-ADP pairs	# of pre-'same region' pairs
$V1 \rightarrow V1$	1.126	3.66e-30	9.16e-31	36	1865	6807	0	2947	2600	83303	0
$HVA \rightarrow HVA$	0.667	1.96e-09	9.79e-10	105	1566	3010	0	3114	2832	106671	0
$V1 \rightarrow HVA$	0.857	1.63e-04	1.63e-04	29	522	2887	0	679	602	16766	0
$HVA \rightarrow V1$	0.861	9.53e-08	7.15e-08	93	1116	6720	0	1445	1315	56628	0

bioRxiv preprint doi: https://doi.org/10.1101/2023.03.13.531369; this version posted October 15, 2024. The copyright holder for this preprint (which was not certified by peer review) is the author/funder, who has granted bioRxiv a license to display the preprint in perpetuity. It is made available under aCC-BY 4.0 International license.

Supplemental Table 11. Number of neurons and neuron pairs invovled in the visualization of the correlation between feature weight similarity and L_d / neuron pair (synapses excluded) in different projection types across brain areas.

Projection type	Δ feature weight similarity bin	# of presynaptic neurons	# of postsynaptic neurons	# of ADP control neurons	# of same region control neurons	# of pre-post pairs	# of pre-ADP pairs	# of pre-'same region' pairs	# of synapses	total L_d (mm)
$V1 \rightarrow V1$	-0.30 - -0.20	36	0	1072	36	0	1295	3229	0	36.335238
$V1 \rightarrow V1$	-0.20 - -0.10	36	0	4396	36	0	9834	25779	0	285.168739
$V1 \rightarrow V1$	-0.10 - 0.00	36	0	5808	36	0	25602	67324	0	768.876584
$V1 \rightarrow V1$	0.00 - 0.10	36	0	5735	36	0	25230	62013	0	783.990533
$V1 \rightarrow V1$	0.10 - 0.20	36	0	4410	36	0	10333	22756	0	335.696665
$V1 \rightarrow V1$	0.20 - 0.30	36	0	1629	36	0	2112	4129	0	69.293019
$V1 \rightarrow V1$	0.30 - 0.40	36	0	301	35	0	318	436	0	10.072933
$HVA \rightarrow HVA$	-0.30 - -0.20	102	0	815	102	0	1392	4489	0	41.334690
$HVA \rightarrow HVA$	-0.20 - -0.10	102	0	2359	102	0	10985	31232	0	335.521478
$HVA \rightarrow HVA$	-0.10 - 0.00	102	0	2619	102	0	30066	78862	0	988.398935
$HVA \rightarrow HVA$	0.00 - 0.10	102	0	2600	102	0	31946	74147	0	1124.617864
$HVA \rightarrow HVA$	0.10 - 0.20	102	0	2358	102	0	13356	27119	0	493.620990
$HVA \rightarrow HVA$	0.20 - 0.30	102	0	1339	102	0	2686	4189	0	109.398149
$HVA \rightarrow HVA$	0.30 - 0.40	90	0	250	76	0	289	358	0	14.959203
$V1 \rightarrow HVA$	-0.20 - -0.10	29	0	1136	29	0	1620	8739	0	40.188636
$V1 \rightarrow HVA$	-0.10 - 0.00	29	0	2146	29	0	5204	28801	0	128.424763
$V1 \rightarrow HVA$	0.00 - 0.10	29	0	2122	29	0	5175	26929	0	126.070146
$V1 \rightarrow HVA$	0.10 - 0.20	29	0	1134	29	0	1729	8279	0	43.275205
$V1 \rightarrow HVA$	0.20 - 0.30	29	0	224	29	0	248	1072	0	6.141092
$HVA \rightarrow V1$	-0.20 - -0.10	92	0	3143	92	0	5593	78638	0	104.073655
$HVA \rightarrow V1$	-0.10 - 0.00	92	0	5364	92	0	17167	231272	0	327.750993
$HVA \rightarrow V1$	0.00 - 0.10	92	0	5214	92	0	17054	220016	0	341.745469
$HVA \rightarrow V1$	0.10 - 0.20	92	0	3111	92	0	6369	73731	0	132.250047
$HVA \rightarrow V1$	0.20 - 0.30	92	0	846	92	0	1111	10568	0	24.540542

bioRxiv preprint doi: https://doi.org/10.1101/2023.03.13.531369; this version posted October 15, 2024. The copyright holder for this preprint (which was not certified by peer review) is the author/funder, who has granted bioRxiv a license to display the preprint in perpetuity. It is made available under aCC-BY 4.0 International license.

Supplemental Table 12. Number of neurons and neuron pairs invovled in the visualization of the correlation between feature weight similarity and $N_{syn}/mm\ L_d$ in different projection types across brain areas.

Projection type	Δ feature weight similarity bin	# of presynaptic neurons	# of postsynaptic neurons	# of ADP control neurons	# of same region control neurons	# of pre-post pairs	# of pre-ADP pairs	# of pre-'same region' pairs	# of synapses	total L_d (mm)
$V1 \rightarrow V1$	-0.30 - -0.20	36	36	1182	0	36	1509	0	38	43.620452
$V1 \rightarrow V1$	-0.20 - -0.10	36	230	4537	0	237	10895	0	258	322.934569
$V1 \rightarrow V1$	-0.10 - 0.00	36	630	5812	0	708	27056	0	794	833.029064
$V1 \rightarrow V1$	0.00 - 0.10	36	729	5720	0	852	25425	0	969	819.256558
$V1 \rightarrow V1$	0.10 - 0.20	36	388	4292	0	438	9940	0	509	337.394190
$V1 \rightarrow V1$	0.20 - 0.30	36	102	1496	0	112	2007	0	139	69.123548
$V1 \rightarrow V1$	0.30 - 0.40	33	24	259	0	24	294	0	31	10.411849
$HVA \rightarrow HVA$	-0.30 - -0.20	99	33	968	0	35	1852	0	37	55.775849
$HVA \rightarrow HVA$	-0.20 - -0.10	99	254	2421	0	276	12617	0	301	398.118425
$HVA \rightarrow HVA$	-0.10 - 0.00	99	663	2619	0	807	32094	0	876	1097.132235
$HVA \rightarrow HVA$	0.00 - 0.10	99	649	2594	0	847	31140	0	926	1149.553761
$HVA \rightarrow HVA$	0.10 - 0.20	99	386	2318	0	450	11911	0	514	461.647156
$HVA \rightarrow HVA$	0.20 - 0.30	98	104	1189	0	109	2216	0	128	98.577172
$HVA \rightarrow HVA$	0.30 - 0.40	71	15	183	0	15	213	0	16	11.813672
$V1 \rightarrow HVA$	-0.20 - -0.10	28	45	1187	0	47	1799	0	50	45.872386
$V1 \rightarrow HVA$	-0.10 - 0.00	29	174	2162	0	179	5474	0	196	139.731979
$V1 \rightarrow HVA$	0.00 - 0.10	29	196	2103	0	204	5296	0	242	132.939401
$V1 \rightarrow HVA$	0.10 - 0.20	29	67	1102	0	70	1684	0	80	44.564126
$V1 \rightarrow HVA$	0.20 - 0.30	27	13	196	0	13	227	0	14	5.948031
$HVA \rightarrow V1$	-0.20 - -0.10	90	106	3354	0	109	6261	0	121	120.205383
$HVA \rightarrow V1$	-0.10 - 0.00	90	370	5397	0	389	18035	0	429	355.383651
$HVA \rightarrow V1$	0.00 - 0.10	90	365	5192	0	396	16927	0	425	348.693858
$HVA \rightarrow V1$	0.10 - 0.20	90	187	2962	0	195	5992	0	218	128.408223
$HVA \rightarrow V1$	0.20 - 0.30	84	34	709	0	36	941	0	45	20.898991

bioRxiv preprint doi: https://doi.org/10.1101/2023.03.13.531369; this version posted October 15, 2024. The copyright holder for this preprint (which was not certified by peer review) is the author/funder, who has granted bioRxiv a license to display the preprint in perpetuity. It is made available under aCC-BY 4.0 International license.

Supplemental Table 13. Estimated marginal means of linear trends for the effect of feature weight similarity on L_d / neuron pair (synapses excluded) in different projection types across brain areas. z and p-value are the z statistics and p-value of the marginal mean linear trends estimated from the fitted GLMMs. adjusted p-value is the adjusted p value through the BH multicomparison correction procedure.

Projection type	Coefficient	adjusted p-value	p-value	# of presynaptic neurons	# of postsynaptic neurons	# of ADP control neurons	# of same region control neurons	# of synapses	# of pre-post pairs	# of pre-ADP pairs	# of pre-'same region' pairs
$V1 \rightarrow V1$	0.947	3.48e-107	1.74e-107	36	0	6237	36	0	0	74829	185807
$HVA \rightarrow HVA$	1.702	0.00e+00	0.00e+00	99	0	2635	99	0	0	89611	212583
$V1 \rightarrow HVA$	0.701	5.01e-10	5.01e-10	29	0	2525	29	0	0	14126	74633
$HVA \rightarrow V1$	1.109	3.18e-94	2.39e-94	90	0	6148	90	0	0	47811	608388

bioRxiv preprint doi: https://doi.org/10.1101/2023.03.13.531369; this version posted October 15, 2024. The copyright holder for this preprint (which was not certified by peer review) is the author/funder, who has granted bioRxiv a license to display the preprint in perpetuity. It is made available under aCC-BY 4.0 International license.

Supplemental Table 14. Estimated marginal means of linear trends for the effect of feature weight similarity on $N_{syn}/mm\ L_d$ in different projection types across brain areas. z and p-value are the z statistics and p-value of the marginal mean linear trends estimated from the fitted GLMMs. adjusted p-value is the adjusted p value through the BH multicomparison correction procedure.

Projection type	Coefficient	adjusted p-value	p-value	# of presynaptic neurons	# of postsynaptic neurons	# of ADP control neurons	# of same region control neurons	# of synapses	# of pre-post pairs	# of pre-ADP pairs	# of pre-'same region' pairs
$V1 \rightarrow V1$	2.216	2.45e-35	6.12e-36	36	1719	6237	0	2744	2411	77240	0
$HVA \rightarrow HVA$	1.398	1.13e-13	5.64e-14	99	1396	2635	0	2803	2543	92154	0
$V1 \rightarrow HVA$	1.754	9.51e-05	9.51e-05	29	448	2525	0	584	515	14641	0
$HVA \rightarrow V1$	1.948	1.49e-11	1.11e-11	90	974	6148	0	1255	1139	48950	0

bioRxiv preprint doi: https://doi.org/10.1101/2023.03.13.531369; this version posted October 15, 2024. The copyright holder for this preprint (which was not certified by peer review) is the author/funder, who has granted bioRxiv a license to display the preprint in perpetuity. It is made available under aCC-BY 4.0 International license.

Supplemental Table 15. Number of neurons and neuron pairs invovled in the visualization of the correlation between receptive field center distance and L_d / neuron pair (synapses excluded) in different projection types across brain areas.

Projection type	Δ receptive field center distance bin	# of presynaptic neurons	# of postsynaptic neurons	# of ADP control neurons	# of same region control neurons	# of pre-post pairs	# of pre-ADP pairs	# of pre-'same region' pairs	# of synapses	total L_d (mm)
$V1 \rightarrow V1$	-10.55 - -5.28	36	0	3587	36	0	13098	16616	0	504.444179
$V1 \rightarrow V1$	-5.28 - 0.00	36	0	5491	36	0	36890	71763	0	1189.389824
$V1 \rightarrow V1$	0.00 - 5.28	36	0	5010	36	0	19930	68768	0	498.660138
$V1 \rightarrow V1$	5.28 - 10.55	36	0	1599	36	0	4266	24166	0	86.320064
$V1 \rightarrow V1$	10.55 - 15.83	36	0	211	36	0	538	4188	0	9.094726
$HVA \rightarrow HVA$	-15.83 - -10.55	44	0	513	44	0	1213	1456	0	53.734766
$HVA \rightarrow HVA$	-10.55 - -5.28	102	0	2281	102	0	17614	26718	0	723.876676
$HVA \rightarrow HVA$	-5.28 - 0.00	102	0	2555	102	0	37564	77929	0	1331.043600
$HVA \rightarrow HVA$	0.00 - 5.28	102	0	2449	102	0	25371	76129	0	770.831112
$HVA \rightarrow HVA$	5.28 - 10.55	102	0	1177	102	0	7797	32087	0	202.978877
$HVA \rightarrow HVA$	10.55 - 15.83	102	0	254	102	0	1139	5848	0	25.291692
$V1 \rightarrow HVA$	-10.55 - -5.28	29	0	985	29	0	2253	11576	0	58.076011
$V1 \rightarrow HVA$	-5.28 - 0.00	29	0	1835	29	0	5668	26875	0	146.551732
$V1 \rightarrow HVA$	0.00 - 5.28	29	0	1623	29	0	4369	23177	0	104.541012
$V1 \rightarrow HVA$	5.28 - 10.55	29	0	620	29	0	1562	10214	0	33.327909
$HVA \rightarrow V1$	-10.55 - -5.28	92	0	3503	92	0	5944	68076	0	127.650382
$HVA \rightarrow V1$	-5.28 - 0.00	92	0	5412	92	0	20967	251016	0	422.868388
$HVA \rightarrow V1$	0.00 - 5.28	92	0	4764	92	0	15792	226867	0	297.769153
$HVA \rightarrow V1$	5.28 - 10.55	92	0	1734	92	0	4574	68340	0	82.355277

bioRxiv preprint doi: https://doi.org/10.1101/2023.03.13.531369; this version posted October 15, 2024. The copyright holder for this preprint (which was not certified by peer review) is the author/funder, who has granted bioRxiv a license to display the preprint in perpetuity. It is made available under aCC-BY 4.0 International license.

Supplemental Table 16. Number of neurons and neuron pairs invovled in the visualization of the correlation between receptive field center distance and $N_{syn}/mm\ L_d$ in different projection types across brain areas.

Projection type	Δ receptive field center distance bin	# of presynaptic neurons	# of postsynaptic neurons	# of ADP control neurons	# of same region control neurons	# of pre-post pairs	# of pre-ADP pairs	# of pre-'same region' pairs	# of synapses	total L_d (mm)
$V1 \to V1$	-10.55 - -5.28	35	168	2274	0	178	5317	0	194	210.846986
$V1 \to V1$	-5.28 - 0.00	36	909	5290	0	1202	36735	0	1380	1313.420605
$V1 \to V1$	0.00 - 5.28	36	695	5647	0	811	27327	0	929	753.483234
$V1 \to V1$	5.28 - 10.55	36	173	2336	0	186	6783	0	204	142.873189
$V1 \to V1$	10.55 - 15.83	34	30	406	0	32	1006	0	35	18.160384
$HVA \to HVA$	-15.83 - -10.55	28	10	243	0	11	416	0	14	17.918761
$HVA \to HVA$	-10.55 - -5.28	99	321	2096	0	378	11349	0	428	500.622918
$HVA \to HVA$	-5.28 - 0.00	99	774	2530	0	1107	37168	0	1220	1438.852620
$HVA \to HVA$	0.00 - 5.28	99	609	2581	0	756	30304	0	827	980.251453
$HVA \to HVA$	5.28 - 10.55	99	199	1584	0	246	10984	0	267	294.805748
$HVA \to HVA$	10.55 - 15.83	99	39	384	0	43	1765	0	45	39.990970
$V1 \to HVA$	-10.55 - -5.28	27	58	819	0	61	1816	0	66	47.293930
$V1 \to HVA$	-5.28 - 0.00	29	203	1796	0	228	5917	0	263	157.158156
$V1 \to HVA$	0.00 - 5.28	29	153	1809	0	158	4861	0	181	123.251450
$V1 \to HVA$	5.28 - 10.55	29	58	754	0	62	1775	0	67	40.294284
$HVA \to V1$	-10.55 - -5.28	86	92	2385	0	93	3677	0	106	73.522190
$HVA \to V1$	-5.28 - 0.00	90	494	5289	0	535	21967	0	584	458.510473
$HVA \to V1$	0.00 - 5.28	90	382	5185	0	416	18503	0	467	368.061118
$HVA \to V1$	5.28 - 10.55	90	85	1770	0	89	4319	0	91	80.279113

bioRxiv preprint doi: https://doi.org/10.1101/2023.03.13.531369; this version posted October 15, 2024. The copyright holder for this preprint (which was not certified by peer review) is the author/funder, who has granted bioRxiv a license to display the preprint in perpetuity. It is made available under aCC-BY 4.0 International license.

Supplemental Table 17. Estimated marginal means of linear trends for the effect of receptive field center distance on L_d / neuron pair (synapses excluded) in different projection types across brain areas. z and p-value are the z statistics and p-value of the marginal mean linear trends estimated from the fitted GLMMs. adjusted p-value is the adjusted p value through the BH multicomparison correction procedure.

Projection type	Coefficient	adjusted p-value	p-value	# of presynaptic neurons	# of postsynaptic neurons	# of ADP control neurons	# of same region control neurons	# of synapses	# of pre-post pairs	# of pre-ADP pairs	# of pre-'same region' pairs
$V1 \rightarrow V1$	-0.127	0.00e+00	0.00e+00	36	0	6237	36	0	0	74829	185807
$HVA \rightarrow HVA$	-0.080	0.00e+00	0.00e+00	99	0	2635	99	0	0	89611	212583
$V1 \rightarrow HVA$	-0.022	1.01e-27	1.01e-27	29	0	2525	29	0	0	14126	74633
$HVA \rightarrow V1$	-0.027	8.19e-119	6.14e-119	90	0	6148	90	0	0	47811	608388

bioRxiv preprint doi: https://doi.org/10.1101/2023.03.13.531369; this version posted October 15, 2024. The copyright holder for this preprint (which was not certified by peer review) is the author/funder, who has granted bioRxiv a license to display the preprint in perpetuity. It is made available under aCC-BY 4.0 International license.

Supplemental Table 18. Estimated marginal means of linear trends for the effect of receptive field center distance on $N_{syn}/mm\ L_d$ in different projection types across brain areas. z and p-value are the z statistics and p-value of the marginal mean linear trends estimated from the fitted GLMMs. adjusted p-value is the adjusted p value through the BH multicomparison correction procedure.

Projection type	Coefficient	adjusted p-value	p-value	# of presynaptic neurons	# of postsynaptic neurons	# of ADP control neurons	# of same region control neurons	# of synapses	# of pre-post pairs	# of pre-ADP pairs	# of pre-'same region' pairs
$V1 \to V1$	0.030	1.89e-09	4.72e-10	36	1719	6237	0	2744	2411	77240	0
$HVA \to HVA$	0.010	1.54e-02	1.15e-02	99	1396	2635	0	2803	2543	92154	0
$V1 \to HVA$	-0.002	8.34e-01	8.34e-01	29	448	2525	0	584	515	14641	0
$HVA \to V1$	-0.018	1.54e-02	9.65e-03	90	974	6148	0	1255	1139	48950	0

bioRxiv preprint doi: https://doi.org/10.1101/2023.03.13.531369; this version posted October 15, 2024. The copyright holder for this preprint (which was not certified by peer review) is the author/funder, who has granted bioRxiv a license to display the preprint in perpetuity. It is made available under aCC-BY 4.0 International license.

Supplemental Table 19. Number of neurons and neuron pairs invovled in the visualization of the correlation between in silico ΔOri and L_d / neuron pair (synapses excluded) in different projection types across brain areas.

Projection type	Δ in silico ΔOri bin	# of presynaptic neurons	# of postsynaptic neurons	# of ADP control neurons	# of same region control neurons	# of pre-post pairs	# of pre-ADP pairs	# of pre-'same region' pairs	# of synapses	total L_d (mm)
$V1 \rightarrow V1$	-57.16 - -28.58	24	0	2307	24	0	5415	15129	0	171.067847
$V1 \rightarrow V1$	-28.58 - 0.00	24	0	2537	24	0	8817	21968	0	295.986023
$V1 \rightarrow V1$	0.00 - 28.58	24	0	2743	24	0	7084	17610	0	221.974391
$V1 \rightarrow V1$	28.58 - 57.16	24	0	2059	24	0	5518	16021	0	162.495971
$HVA \rightarrow HVA$	-57.16 - -28.58	60	0	1125	60	0	5729	13469	0	194.286636
$HVA \rightarrow HVA$	-28.58 - 0.00	60	0	1175	60	0	6887	15960	0	235.669897
$HVA \rightarrow HVA$	0.00 - 28.58	60	0	1179	60	0	6905	16323	0	231.975979
$HVA \rightarrow HVA$	28.58 - 57.16	60	0	1129	60	0	5634	13479	0	185.273829
$V1 \rightarrow HVA$	-57.16 - -28.58	18	0	502	18	0	1017	4946	0	26.483564
$V1 \rightarrow HVA$	-28.58 - 0.00	18	0	663	18	0	1232	5295	0	35.218815
$V1 \rightarrow HVA$	0.00 - 28.58	18	0	654	18	0	1179	5792	0	31.287205
$V1 \rightarrow HVA$	28.58 - 57.16	18	0	463	18	0	797	5100	0	20.293288
$HVA \rightarrow V1$	-57.16 - -28.58	53	0	1937	53	0	3351	41162	0	66.825000
$HVA \rightarrow V1$	-28.58 - 0.00	53	0	2540	53	0	5802	59529	0	120.864783
$HVA \rightarrow V1$	0.00 - 28.58	53	0	2630	53	0	5786	56326	0	123.706798
$HVA \rightarrow V1$	28.58 - 57.16	53	0	2011	53	0	3495	41530	0	70.913483

bioRxiv preprint doi: https://doi.org/10.1101/2023.03.13.531369; this version posted October 15, 2024. The copyright holder for this preprint (which was not certified by peer review) is the author/funder, who has granted bioRxiv a license to display the preprint in perpetuity. It is made available under aCC-BY 4.0 International license.

Supplemental Table 20. Number of neurons and neuron pairs invovled in the visualization of the correlation between in silico ΔOri and $N_{syn}/mm\ L_d$ in different projection types across brain areas.

Projection type	Δ in silico ΔOri bin	# of presynaptic neurons	# of postsynaptic neurons	# of ADP control neurons	# of same region control neurons	# of pre-post pairs	# of pre-ADP pairs	# of pre-'same region' pairs	# of synapses	total L_d (mm)
$V1 \rightarrow V1$	-57.16 - -28.58	24	184	2256	0	203	5252	0	254	171.406452
$V1 \rightarrow V1$	-28.58 - 0.00	24	302	2524	0	336	9435	0	394	329.222605
$V1 \rightarrow V1$	0.00 - 28.58	24	217	2816	0	241	7546	0	268	245.866967
$V1 \rightarrow V1$	28.58 - 57.16	24	144	1979	0	157	5538	0	180	166.321087
$HVA \rightarrow HVA$	-57.16 - -28.58	52	134	1113	0	155	5274	0	169	190.852637
$HVA \rightarrow HVA$	-28.58 - 0.00	52	169	1165	0	180	6698	0	193	241.124324
$HVA \rightarrow HVA$	0.00 - 28.58	52	157	1179	0	173	6701	0	181	240.263087
$HVA \rightarrow HVA$	28.58 - 57.16	52	129	1122	0	150	5299	0	165	182.145706
$V1 \rightarrow HVA$	-57.16 - -28.58	16	39	479	0	40	854	0	48	23.297975
$V1 \rightarrow HVA$	-28.58 - 0.00	16	35	655	0	36	1373	0	45	40.063947
$V1 \rightarrow HVA$	0.00 - 28.58	16	50	678	0	51	1216	0	54	34.018144
$V1 \rightarrow HVA$	28.58 - 57.16	16	28	481	0	30	925	0	33	24.334127
$HVA \rightarrow V1$	-57.16 - -28.58	46	73	1831	0	75	3198	0	81	66.090308
$HVA \rightarrow V1$	-28.58 - 0.00	47	138	2502	0	140	5974	0	159	128.419254
$HVA \rightarrow V1$	0.00 - 28.58	47	140	2641	0	149	5964	0	158	129.941363
$HVA \rightarrow V1$	28.58 - 57.16	47	63	1867	0	64	3187	0	69	67.486736

bioRxiv preprint doi: https://doi.org/10.1101/2023.03.13.531369; this version posted October 15, 2024. The copyright holder for this preprint (which was not certified by peer review) is the author/funder, who has granted bioRxiv a license to display the preprint in perpetuity. It is made available under aCC-BY 4.0 International license.

Supplemental Table 21. Estimated marginal means of linear trends for the effect of in silico ΔOri on L_d / neuron pair (synapses excluded) in different projection types across brain areas. z and p-value are the z statistics and p-value of the marginal mean linear trends estimated from the fitted GLMMs. adjusted p-value is the adjusted p value through the BH multicomparison correction procedure.

Projection type	Coefficient	adjusted p-value	p-value	# of presynaptic neurons	# of postsynaptic neurons	# of ADP control neurons	# of same region control neurons	# of synapses	# of pre-post pairs	# of pre-ADP pairs	# of pre-'same region' pairs
$V1 \rightarrow V1$	-0.001	1.22e-05	6.11e-06	24	0	3456	24	0	0	26834	70728
$HVA \rightarrow HVA$	-0.001	4.70e-02	4.70e-02	52	0	1222	52	0	0	23314	49735
$V1 \rightarrow HVA$	-0.004	4.65e-08	1.16e-08	16	0	1123	16	0	0	4211	18313
$HVA \rightarrow V1$	0.001	3.02e-02	2.27e-02	47	0	3392	47	0	0	17907	174465

bioRxiv preprint doi: https://doi.org/10.1101/2023.03.13.531369; this version posted October 15, 2024. The copyright holder for this preprint (which was not certified by peer review) is the author/funder, who has granted bioRxiv a license to display the preprint in perpetuity. It is made available under aCC-BY 4.0 International license.

Supplemental Table 22. Estimated marginal means of linear trends for the effect of in silico ΔOri on $N_{syn}/mm\ L_d$ in different projection types across brain areas. z and p-value are the z statistics and p-value of the marginal mean linear trends estimated from the fitted GLMMs. adjusted p-value is the adjusted p value through the BH multicomparison correction procedure.

Projection type	Coefficient	adjusted p-value	p-value	# of presynaptic neurons	# of postsynaptic neurons	# of ADP control neurons	# of same region control neurons	# of synapses	# of pre-post pairs	# of pre-ADP pairs	# of pre-'same region' pairs
$V1 \rightarrow V1$	-0.004	1.12e-03	2.79e-04	24	739	3456	0	1096	937	27771	0
$HVA \rightarrow HVA$	0.000	9.36e-01	9.36e-01	52	452	1222	0	708	658	23972	0
$V1 \rightarrow HVA$	-0.004	2.33e-01	1.75e-01	16	147	1123	0	180	157	4368	0
$HVA \rightarrow V1$	-0.002	2.33e-01	1.68e-01	47	392	3392	0	467	428	18335	0

bioRxiv preprint doi: https://doi.org/10.1101/2023.03.13.531369; this version posted October 15, 2024. The copyright holder for this preprint (which was not certified by peer review) is the author/funder, who has granted bioRxiv a license to display the preprint in perpetuity. It is made available under aCC-BY 4.0 International license.

Supplemental Table 23. Estimated marginal means of linear trends for the effect of in vivo signal correlation on L_d / neuron pair (synapses excluded) in different projection types across brain areas and layers. z and p-value are the z statistics and p-value of the marginal mean linear trends estimated from the fitted GLMMs. adjusted p-value is the adjusted p value through the BH multicomparison correction procedure.

Projection type	Coefficient	adjusted p-value	p-value	# of presynaptic neurons	# of postsynaptic neurons	# of ADP control neurons	# of same region control neurons	# of synapses	# of pre-post pairs	# of pre-ADP pairs	# of pre-'same region' pairs
$V1L2/3 \to V1L2/3$	1.074	2.49e-109	2.08e-110	20	0	2670	20	0	0	20511	44349
$V1L2/3 \to V1L4$	0.341	1.52e-07	7.59e-08	19	0	2090	19	0	0	13791	32623
$V1L2/3 \to V1L5$	0.580	6.10e-18	1.27e-18	20	0	1185	20	0	0	11845	14652
$V1L2/3 \to HVAL2/3$	1.210	1.21e-25	2.02e-26	15	0	1169	15	0	0	4610	18075
$V1L2/3 \to HVAL4$	0.798	1.26e-07	5.78e-08	14	0	856	14	0	0	3202	10900
$V1L2/3 \to HVAL5$	1.136	1.84e-11	4.61e-12	13	0	429	13	0	0	2444	4028
$V1L4 \to V1L2/3$	0.335	5.41e-03	4.51e-03	6	0	1784	6	0	0	3107	16451
$V1L4 \to V1L4$	0.422	1.22e-04	8.12e-05	6	0	1865	6	0	0	4503	10073
$V1L4 \to V1L5$	0.435	1.04e-04	6.51e-05	6	0	1138	6	0	0	3365	4636
$V1L5 \to V1L4$	0.407	1.85e-04	1.31e-04	6	0	1769	6	0	0	3980	10686
$V1L5 \to V1L5$	0.523	6.94e-09	2.60e-09	6	0	1145	6	0	0	3721	4280
$HVAL2/3 \to V1L2/3$	0.067	3.37e-01	3.09e-01	36	0	2626	36	0	0	12670	104893
$HVAL2/3 \to V1L4$	-0.024	8.66e-01	8.30e-01	28	0	1882	28	0	0	5122	63511
$HVAL2/3 \to V1L5$	0.361	4.28e-08	1.78e-08	59	0	1172	59	0	0	12966	66476
$HVAL2/3 \to HVAL2/3$	1.089	3.19e-121	1.33e-122	45	0	1264	45	0	0	19194	48691
$HVAL2/3 \to HVAL4$	0.831	3.91e-42	4.89e-43	38	0	893	38	0	0	13326	24937
$HVAL2/3 \to HVAL5$	0.280	1.10e-05	6.07e-06	62	0	439	62	0	0	13451	17560
$HVAL4 \to HVAL2/3$	0.633	5.28e-09	1.76e-09	12	0	1233	12	0	0	5899	12092
$HVAL4 \to HVAL4$	0.679	1.93e-09	5.62e-10	12	0	893	12	0	0	5266	6729
$HVAL4 \to HVAL5$	0.355	7.93e-03	6.94e-03	11	0	434	11	0	0	2992	2477
$HVAL5 \to V1L5$	-0.013	9.11e-01	9.11e-01	14	0	1093	14	0	0	3539	15315
$HVAL5 \to HVAL2/3$	0.332	1.38e-03	1.04e-03	17	0	1236	17	0	0	7564	18063
$HVAL5 \to HVAL4$	0.326	4.43e-03	3.51e-03	17	0	896	17	0	0	6110	11017
$HVAL5 \to HVAL5$	0.458	1.10e-05	6.39e-06	19	0	439	19	0	0	5390	4009

bioRxiv preprint doi: https://doi.org/10.1101/2023.03.13.531369; this version posted October 15, 2024. The copyright holder for this preprint (which was not certified by peer review) is the author/funder, who has granted bioRxiv a license to display the preprint in perpetuity. It is made available under aCC-BY 4.0 International license.

Supplemental Table 24. Estimated marginal means of linear trends for the effect of in vivo signal correlation on $N_{syn}/mm\ L_d$ in different projection types across brain areas and layers. z and p-value are the z statistics and p-value of the marginal mean linear trends estimated from the fitted GLMMs. adjusted p-value is the adjusted p value through the BH multicomparison correction procedure.

Projection type	Coefficient	adjusted p-value	p-value	# of presynaptic neurons	# of postsynaptic neurons	# of ADP control neurons	# of same region control neurons	# of synapses	# of pre-post pairs	# of pre-ADP pairs	# of pre-'same region' pairs
$V1L2/3 \rightarrow V1L2/3$	1.110	2.08e-06	1.73e-07	20	604	2670	0	792	736	21247	0
$V1L2/3 \rightarrow V1L4$	0.617	1.33e-01	8.35e-02	19	197	2090	0	235	219	14010	0
$V1L2/3 \rightarrow V1L5$	1.820	3.11e-19	1.29e-20	20	311	1185	0	687	539	12384	0
$V1L2/3 \rightarrow HVAL2/3$	0.618	2.18e-01	1.54e-01	15	176	1169	0	208	196	4806	0
$V1L2/3 \rightarrow HVAL4$	1.080	1.33e-01	8.90e-02	14	80	856	0	89	82	3284	0
$V1L2/3 \rightarrow HVAL5$	1.225	3.34e-02	1.81e-02	13	91	429	0	131	106	2550	0
$V1L4 \rightarrow V1L2/3$	0.674	2.20e-01	1.65e-01	6	108	1784	0	120	110	3217	0
$V1L4 \rightarrow V1L4$	1.162	1.10e-02	4.57e-03	6	141	1865	0	155	146	4649	0
$V1L4 \rightarrow V1L5$	1.759	4.71e-05	7.85e-06	6	101	1138	0	130	110	3475	0
$V1L5 \rightarrow V1L4$	-1.058	2.24e-01	1.78e-01	6	64	1769	0	65	64	4044	0
$V1L5 \rightarrow V1L5$	0.916	2.64e-02	1.21e-02	6	103	1145	0	121	104	3825	0
$HVAL2/3 \rightarrow V1L2/3$	0.880	8.21e-03	3.08e-03	36	381	2626	0	436	411	13081	0
$HVAL2/3 \rightarrow V1L4$	1.346	6.96e-02	4.06e-02	28	79	1882	0	88	81	5203	0
$HVAL2/3 \rightarrow V1L5$	1.378	4.12e-05	5.16e-06	59	213	1172	0	324	278	13244	0
$HVAL2/3 \rightarrow HVAL2/3$	-0.083	7.29e-01	6.98e-01	45	519	1264	0	801	732	19926	0
$HVAL2/3 \rightarrow HVAL4$	1.223	4.13e-04	1.03e-04	38	204	893	0	301	258	13584	0
$HVAL2/3 \rightarrow HVAL5$	1.188	5.20e-05	1.08e-05	62	216	439	0	410	361	13812	0
$HVAL4 \rightarrow HVAL2/3$	0.843	3.34e-02	1.70e-02	12	259	1233	0	334	316	6215	0
$HVAL4 \rightarrow HVAL4$	1.349	8.21e-03	2.89e-03	12	138	893	0	174	155	5421	0
$HVAL4 \rightarrow HVAL5$	1.836	4.71e-03	1.37e-03	11	89	434	0	108	97	3089	0
$HVAL5 \rightarrow V1L5$	-0.416	6.32e-01	5.79e-01	14	59	1093	0	67	62	3601	0
$HVAL5 \rightarrow HVAL2/3$	0.094	8.17e-01	8.17e-01	17	260	1236	0	331	308	7872	0
$HVAL5 \rightarrow HVAL4$	0.672	3.11e-01	2.62e-01	17	92	896	0	110	102	6212	0
$HVAL5 \rightarrow HVAL5$	0.460	3.11e-01	2.72e-01	19	148	439	0	214	196	5586	0

bioRxiv preprint doi: https://doi.org/10.1101/2023.03.13.531369; this version posted October 15, 2024. The copyright holder for this preprint (which was not certified by peer review) is the author/funder, who has granted bioRxiv a license to display the preprint in perpetuity. It is made available under aCC-BY 4.0 International license.

Supplemental Table 25. Estimated marginal means of linear trends for the effect of in silico signal correlation on L_d / neuron pair (synapses excluded) in different projection types across brain areas and layers. z and p-value are the z statistics and p-value of the marginal mean linear trends estimated from the fitted GLMMs. adjusted p-value is the adjusted p value through the BH multicomparison correction procedure.

Projection type	Coefficient	adjusted p-value	p-value	# of presynaptic neurons	# of postsynaptic neurons	# of ADP control neurons	# of same region control neurons	# of synapses	# of pre-post pairs	# of pre-ADP pairs	# of pre-'same region' pairs
$V1L2/3 \rightarrow V1L2/3$	1.692	6.20e-109	5.17e-110	20	0	2670	20	0	0	20511	44349
$V1L2/3 \rightarrow V1L4$	0.632	9.58e-10	5.19e-10	19	0	2090	19	0	0	13791	32623
$V1L2/3 \rightarrow V1L5$	0.929	1.39e-17	5.23e-18	20	0	1185	20	0	0	11845	14652
$V1L2/3 \rightarrow HVAL2/3$	2.276	5.44e-33	9.06e-34	15	0	1169	15	0	0	4610	18075
$V1L2/3 \rightarrow HVAL4$	1.520	4.26e-10	2.13e-10	14	0	856	14	0	0	3202	10900
$V1L2/3 \rightarrow HVAL5$	0.738	5.58e-03	4.65e-03	13	0	429	13	0	0	2444	4028
$V1L4 \rightarrow V1L2/3$	0.551	7.48e-03	6.54e-03	6	0	1784	6	0	0	3107	16451
$V1L4 \rightarrow V1L4$	0.525	1.65e-03	1.25e-03	6	0	1865	6	0	0	4503	10073
$V1L4 \rightarrow V1L5$	0.538	1.65e-03	1.31e-03	6	0	1138	6	0	0	3365	4636
$V1L5 \rightarrow V1L4$	0.491	7.49e-03	6.87e-03	6	0	1769	6	0	0	3980	10686
$V1L5 \rightarrow V1L5$	0.546	8.97e-04	5.98e-04	6	0	1145	6	0	0	3721	4280
$HVAL2/3 \rightarrow V1L2/3$	-0.020	8.30e-01	8.30e-01	36	0	2626	36	0	0	12670	104893
$HVAL2/3 \rightarrow V1L4$	0.473	1.45e-03	1.03e-03	28	0	1882	28	0	0	5122	63511
$HVAL2/3 \rightarrow V1L5$	0.973	7.73e-29	1.61e-29	59	0	1172	59	0	0	12966	66476
$HVAL2/3 \rightarrow HVAL2/3$	2.087	3.32e-246	1.38e-247	45	0	1264	45	0	0	19194	48691
$HVAL2/3 \rightarrow HVAL4$	1.410	2.16e-69	2.70e-70	38	0	893	38	0	0	13326	24937
$HVAL2/3 \rightarrow HVAL5$	0.116	1.45e-01	1.39e-01	62	0	439	62	0	0	13451	17560
$HVAL4 \rightarrow HVAL2/3$	1.264	2.34e-22	6.83e-23	12	0	1233	12	0	0	5899	12092
$HVAL4 \rightarrow HVAL4$	0.975	1.87e-13	8.58e-14	12	0	893	12	0	0	5266	6729
$HVAL4 \rightarrow HVAL5$	0.631	1.69e-04	1.05e-04	11	0	434	11	0	0	2992	2477
$HVAL5 \rightarrow V1L5$	0.873	5.35e-07	3.12e-07	14	0	1093	14	0	0	3539	15315
$HVAL5 \rightarrow HVAL2/3$	1.266	3.38e-24	8.45e-25	17	0	1236	17	0	0	7564	18063
$HVAL5 \rightarrow HVAL4$	1.146	1.40e-16	5.83e-17	17	0	896	17	0	0	6110	11017
$HVAL5 \rightarrow HVAL5$	1.215	9.66e-22	3.22e-22	19	0	439	19	0	0	5390	4009

bioRxiv preprint doi: https://doi.org/10.1101/2023.03.13.531369; this version posted October 15, 2024. The copyright holder for this preprint (which was not certified by peer review) is the author/funder, who has granted bioRxiv a license to display the preprint in perpetuity. It is made available under aCC-BY 4.0 International license.

Supplemental Table 26. Estimated marginal means of linear trends for the effect of in silico signal correlation on $N_{syn}/mm\ L_d$ in different projection types across brain areas and layers. z and p-value are the z statistics and p-value of the marginal mean linear trends estimated from the fitted GLMMs. adjusted p-value is the adjusted p value through the BH multicomparison correction procedure.

Projection type	Coefficient	adjusted p-value	p-value	# of presynaptic neurons	# of postsynaptic neurons	# of ADP control neurons	# of same region control neurons	# of synapses	# of pre-post pairs	# of pre-ADP pairs	# of pre-'same region' pairs
$V1L2/3 \rightarrow V1L2/3$	2.026	2.87e-09	3.58e-10	20	604	2670	0	792	736	21247	0
$V1L2/3 \rightarrow V1L4$	2.973	1.67e-07	3.47e-08	19	197	2090	0	235	219	14010	0
$V1L2/3 \rightarrow V1L5$	4.022	1.75e-39	7.29e-41	20	311	1185	0	687	539	12384	0
$V1L2/3 \rightarrow HVAL2/3$	0.808	3.52e-01	2.93e-01	15	176	1169	0	208	196	4806	0
$V1L2/3 \rightarrow HVAL4$	2.167	9.18e-02	6.12e-02	14	80	856	0	89	82	3284	0
$V1L2/3 \rightarrow HVAL5$	3.941	3.48e-06	1.16e-06	13	91	429	0	131	106	2550	0
$V1L4 \rightarrow V1L2/3$	1.097	2.48e-01	1.86e-01	6	108	1784	0	120	110	3217	0
$V1L4 \rightarrow V1L4$	2.180	5.08e-04	1.90e-04	6	141	1865	0	155	146	4649	0
$V1L4 \rightarrow V1L5$	3.194	7.21e-09	1.20e-09	6	101	1138	0	130	110	3475	0
$V1L5 \rightarrow V1L4$	-1.271	3.52e-01	2.87e-01	6	64	1769	0	65	64	4044	0
$V1L5 \rightarrow V1L5$	1.508	5.43e-02	2.94e-02	6	103	1145	0	121	104	3825	0
$HVAL2/3 \rightarrow V1L2/3$	0.837	9.18e-02	5.90e-02	36	381	2626	0	436	411	13081	0
$HVAL2/3 \rightarrow V1L4$	1.304	2.15e-01	1.52e-01	28	79	1882	0	88	81	5203	0
$HVAL2/3 \rightarrow V1L5$	2.730	1.70e-10	1.42e-11	59	213	1172	0	324	278	13244	0
$HVAL2/3 \rightarrow HVAL2/3$	-0.108	7.41e-01	7.19e-01	45	519	1264	0	801	732	19926	0
$HVAL2/3 \rightarrow HVAL4$	1.585	9.99e-04	4.16e-04	38	204	893	0	301	258	13584	0
$HVAL2/3 \rightarrow HVAL5$	1.834	2.24e-06	5.61e-07	62	216	439	0	410	361	13812	0
$HVAL4 \rightarrow HVAL2/3$	1.203	2.36e-02	1.18e-02	12	259	1233	0	334	316	6215	0
$HVAL4 \rightarrow HVAL4$	2.515	2.28e-06	6.64e-07	12	138	893	0	174	155	5421	0
$HVAL4 \rightarrow HVAL5$	2.154	5.01e-03	2.30e-03	11	89	434	0	108	97	3089	0
$HVAL5 \rightarrow V1L5$	1.878	8.64e-02	5.04e-02	14	59	1093	0	67	62	3601	0
$HVAL5 \rightarrow HVAL2/3$	0.308	5.91e-01	5.17e-01	17	260	1236	0	331	308	7872	0
$HVAL5 \rightarrow HVAL4$	0.248	7.41e-01	7.41e-01	17	92	896	0	110	102	6212	0
$HVAL5 \rightarrow HVAL5$	0.313	6.00e-01	5.50e-01	19	148	439	0	214	196	5586	0

bioRxiv preprint doi: https://doi.org/10.1101/2023.03.13.531369; this version posted October 15, 2024. The copyright holder for this preprint (which was not certified by peer review) is the author/funder, who has granted bioRxiv a license to display the preprint in perpetuity. It is made available under aCC-BY 4.0 International license.

Supplemental Table 27. Estimated marginal means of linear trends for the effect of feature weight similarity on L_d / neuron pair (synapses excluded) in different projection types across brain areas and layers. z and p-value are the z statistics and p-value of the marginal mean linear trends estimated from the fitted GLMMs. adjusted p-value is the adjusted p value through the BH multicomparison correction procedure.

Projection type	Coefficient	adjusted p-value	p-value	# of presynaptic neurons	# of postsynaptic neurons	# of ADP control neurons	# of same region control neurons	# of synapses	# of pre-post pairs	# of pre-ADP pairs	# of pre-'same region' pairs
$V1L2/3 \rightarrow V1L2/3$	1.005	8.41e-38	1.05e-38	20	0	2670	20	0	0	20511	44349
$V1L2/3 \rightarrow V1L4$	0.512	9.17e-07	4.59e-07	19	0	2090	19	0	0	13791	32623
$V1L2/3 \rightarrow V1L5$	0.602	8.40e-08	3.85e-08	20	0	1185	20	0	0	11845	14652
$V1L2/3 \rightarrow HVAL2/3$	0.849	1.12e-05	6.97e-06	15	0	1169	15	0	0	4610	18075
$V1L2/3 \rightarrow HVAL4$	0.804	1.27e-03	9.01e-04	14	0	856	14	0	0	3202	10900
$V1L2/3 \rightarrow HVAL5$	0.410	1.34e-01	1.28e-01	13	0	429	13	0	0	2444	4028
$V1L4 \rightarrow V1L2/3$	0.257	2.09e-01	2.09e-01	6	0	1784	6	0	0	3107	16451
$V1L4 \rightarrow V1L4$	0.769	1.79e-06	9.70e-07	6	0	1865	6	0	0	4503	10073
$V1L4 \rightarrow V1L5$	0.663	2.60e-04	1.73e-04	6	0	1138	6	0	0	3365	4636
$V1L5 \rightarrow V1L4$	0.465	1.66e-02	1.25e-02	6	0	1769	6	0	0	3980	10686
$V1L5 \rightarrow V1L5$	0.361	3.27e-02	2.87e-02	6	0	1145	6	0	0	3721	4280
$HVAL2/3 \rightarrow V1L2/3$	0.251	2.75e-02	2.17e-02	36	0	2626	36	0	0	12670	104893
$HVAL2/3 \rightarrow V1L4$	0.378	3.17e-02	2.64e-02	28	0	1882	28	0	0	5122	63511
$HVAL2/3 \rightarrow V1L5$	1.078	5.07e-22	1.06e-22	59	0	1172	59	0	0	12966	66476
$HVAL2/3 \rightarrow HVAL2/3$	2.692	3.67e-248	1.53e-249	45	0	1264	45	0	0	19194	48691
$HVAL2/3 \rightarrow HVAL4$	2.013	5.69e-83	4.74e-84	38	0	893	38	0	0	13326	24937
$HVAL2/3 \rightarrow HVAL5$	0.885	1.56e-17	5.20e-18	62	0	439	62	0	0	13451	17560
$HVAL4 \rightarrow HVAL2/3$	1.560	2.27e-24	3.78e-25	12	0	1233	12	0	0	5899	12092
$HVAL4 \rightarrow HVAL4$	1.436	1.03e-21	2.57e-22	12	0	893	12	0	0	5266	6729
$HVAL4 \rightarrow HVAL5$	0.884	2.15e-06	1.25e-06	11	0	434	11	0	0	2992	2477
$HVAL5 \rightarrow V1L5$	1.169	1.05e-09	4.38e-10	14	0	1093	14	0	0	3539	15315
$HVAL5 \rightarrow HVAL2/3$	0.273	6.10e-02	5.59e-02	17	0	1236	17	0	0	7564	18063
$HVAL5 \rightarrow HVAL4$	1.195	3.89e-14	1.46e-14	17	0	896	17	0	0	6110	11017
$HVAL5 \rightarrow HVAL5$	1.306	1.37e-19	3.99e-20	19	0	439	19	0	0	5390	4009

bioRxiv preprint doi: https://doi.org/10.1101/2023.03.13.531369; this version posted October 15, 2024. The copyright holder for this preprint (which was not certified by peer review) is the author/funder, who has granted bioRxiv a license to display the preprint in perpetuity. It is made available under aCC-BY 4.0 International license.

Supplemental Table 28. Estimated marginal means of linear trends for the effect of feature weight similarity on $N_{syn}/mm\ L_d$ in different projection types across brain areas and layers. z and p-value are the z statistics and p-value of the marginal mean linear trends estimated from the fitted GLMMs. adjusted p-value is the adjusted p value through the BH multicomparison correction procedure.

Projection type	Coefficient	adjusted p-value	p-value	# of presynaptic neurons	# of postsynaptic neurons	# of ADP control neurons	# of same region control neurons	# of synapses	# of pre-post pairs	# of pre-ADP pairs	# of pre-'same region' pairs
$V1L2/3 \rightarrow V1L2/3$	1.840	6.63e-07	1.11e-07	20	604	2670	0	792	736	21247	0
$V1L2/3 \rightarrow V1L4$	2.015	3.78e-03	1.57e-03	19	197	2090	0	235	219	14010	0
$V1L2/3 \rightarrow V1L5$	3.855	1.05e-25	4.39e-27	20	311	1185	0	687	539	12384	0
$V1L2/3 \rightarrow HVAL2/3$	0.715	3.77e-01	3.45e-01	15	176	1169	0	208	196	4806	0
$V1L2/3 \rightarrow HVAL4$	-0.143	9.04e-01	9.04e-01	14	80	856	0	89	82	3284	0
$V1L2/3 \rightarrow HVAL5$	3.330	4.87e-04	1.83e-04	13	91	429	0	131	106	2550	0
$V1L4 \rightarrow V1L2/3$	1.005	3.28e-01	2.59e-01	6	108	1784	0	120	110	3217	0
$V1L4 \rightarrow V1L4$	2.416	4.52e-04	1.51e-04	6	141	1865	0	155	146	4649	0
$V1L4 \rightarrow V1L5$	4.052	8.61e-08	7.18e-09	6	101	1138	0	130	110	3475	0
$V1L5 \rightarrow V1L4$	-1.410	3.11e-01	2.33e-01	6	64	1769	0	65	64	4044	0
$V1L5 \rightarrow V1L5$	0.304	7.36e-01	7.05e-01	6	103	1145	0	121	104	3825	0
$HVAL2/3 \rightarrow V1L2/3$	1.248	3.23e-02	1.89e-02	36	381	2626	0	436	411	13081	0
$HVAL2/3 \rightarrow V1L4$	3.178	8.70e-03	4.35e-03	28	79	1882	0	88	81	5203	0
$HVAL2/3 \rightarrow V1L5$	3.210	1.28e-07	1.59e-08	59	213	1172	0	324	278	13244	0
$HVAL2/3 \rightarrow HVAL2/3$	0.351	3.77e-01	3.44e-01	45	519	1264	0	801	732	19926	0
$HVAL2/3 \rightarrow HVAL4$	2.861	7.44e-06	1.86e-06	38	204	893	0	301	258	13584	0
$HVAL2/3 \rightarrow HVAL5$	2.452	3.18e-06	6.63e-07	62	216	439	0	410	361	13812	0
$HVAL4 \rightarrow HVAL2/3$	1.416	2.23e-02	1.21e-02	12	259	1233	0	334	316	6215	0
$HVAL4 \rightarrow HVAL4$	2.614	1.54e-04	4.51e-05	12	138	893	0	174	155	5421	0
$HVAL4 \rightarrow HVAL5$	2.486	8.70e-03	4.12e-03	11	89	434	0	108	97	3089	0
$HVAL5 \rightarrow V1L5$	1.423	2.89e-01	2.05e-01	14	59	1093	0	67	62	3601	0
$HVAL5 \rightarrow HVAL2/3$	0.794	2.51e-01	1.67e-01	17	260	1236	0	331	308	7872	0
$HVAL5 \rightarrow HVAL4$	-0.926	3.54e-01	2.95e-01	17	92	896	0	110	102	6212	0
$HVAL5 \rightarrow HVAL5$	1.090	1.09e-01	6.81e-02	19	148	439	0	214	196	5586	0

bioRxiv preprint doi: https://doi.org/10.1101/2023.03.13.531369; this version posted October 15, 2024. The copyright holder for this preprint (which was not certified by peer review) is the author/funder, who has granted bioRxiv a license to display the preprint in perpetuity. It is made available under aCC-BY 4.0 International license.

Supplemental Table 29. Estimated marginal means of linear trends for the effect of receptive field center distance on L_d / neuron pair (synapses excluded) in different projection types across brain areas and layers. z and p-value are the z statistics and p-value of the marginal mean linear trends estimated from the fitted GLMMs. adjusted p-value is the adjusted p value through the BH multicomparison correction procedure.

Projection type	Coefficient	adjusted p-value	p-value	# of presynaptic neurons	# of postsynaptic neurons	# of ADP control neurons	# of same region control neurons	# of synapses	# of pre-post pairs	# of pre-ADP pairs	# of pre-'same region' pairs
$V1L2/3 \rightarrow V1L2/3$	-0.167	0.00e+00	0.00e+00	20	0	2670	20	0	0	20511	44349
$V1L2/3 \rightarrow V1L4$	-0.141	0.00e+00	0.00e+00	19	0	2090	19	0	0	13791	32623
$V1L2/3 \rightarrow V1L5$	-0.111	0.00e+00	0.00e+00	20	0	1185	20	0	0	11845	14652
$V1L2/3 \rightarrow HVAL2/3$	-0.032	7.39e-21	6.16e-21	15	0	1169	15	0	0	4610	18075
$V1L2/3 \rightarrow HVAL4$	-0.030	5.27e-14	4.83e-14	14	0	856	14	0	0	3202	10900
$V1L2/3 \rightarrow HVAL5$	-0.014	4.94e-03	4.94e-03	13	0	429	13	0	0	2444	4028
$V1L4 \rightarrow V1L2/3$	-0.097	1.11e-68	7.42e-69	6	0	1784	6	0	0	3107	16451
$V1L4 \rightarrow V1L4$	-0.136	1.76e-189	5.14e-190	6	0	1865	6	0	0	4503	10073
$V1L4 \rightarrow V1L5$	-0.134	8.03e-134	4.01e-134	6	0	1138	6	0	0	3365	4636
$V1L5 \rightarrow V1L4$	-0.053	2.71e-27	2.14e-27	6	0	1769	6	0	0	3980	10686
$V1L5 \rightarrow V1L5$	-0.092	1.19e-80	7.44e-81	6	0	1145	6	0	0	3721	4280
$HVAL2/3 \rightarrow V1L2/3$	-0.015	7.37e-11	7.06e-11	36	0	2626	36	0	0	12670	104893
$HVAL2/3 \rightarrow V1L4$	-0.031	1.82e-16	1.60e-16	28	0	1882	28	0	0	5122	63511
$HVAL2/3 \rightarrow V1L5$	-0.035	1.25e-46	9.39e-47	59	0	1172	59	0	0	12966	66476
$HVAL2/3 \rightarrow HVAL2/3$	-0.099	0.00e+00	0.00e+00	45	0	1264	45	0	0	19194	48691
$HVAL2/3 \rightarrow HVAL4$	-0.083	0.00e+00	0.00e+00	38	0	893	38	0	0	13326	24937
$HVAL2/3 \rightarrow HVAL5$	-0.047	1.41e-112	7.63e-113	62	0	439	62	0	0	13451	17560
$HVAL4 \rightarrow HVAL2/3$	-0.084	4.83e-169	1.81e-169	12	0	1233	12	0	0	5899	12092
$HVAL4 \rightarrow HVAL4$	-0.093	2.33e-215	5.82e-216	12	0	893	12	0	0	5266	6729
$HVAL4 \rightarrow HVAL5$	-0.109	5.56e-172	1.85e-172	11	0	434	11	0	0	2992	2477
$HVAL5 \rightarrow V1L5$	-0.067	3.07e-53	2.18e-53	14	0	1093	14	0	0	3539	15315
$HVAL5 \rightarrow HVAL2/3$	-0.052	3.63e-86	2.12e-86	17	0	1236	17	0	0	7564	18063
$HVAL5 \rightarrow HVAL4$	-0.071	8.03e-134	3.74e-134	17	0	896	17	0	0	6110	11017
$HVAL5 \rightarrow HVAL5$	-0.082	4.99e-159	2.08e-159	19	0	439	19	0	0	5390	4009

bioRxiv preprint doi: https://doi.org/10.1101/2023.03.13.531369; this version posted October 15, 2024. The copyright holder for this preprint (which was not certified by peer review) is the author/funder, who has granted bioRxiv a license to display the preprint in perpetuity. It is made available under aCC-BY 4.0 International license.

Supplemental Table 30. Estimated marginal means of linear trends for the effect of receptive field center distance on $N_{syn}/mm\ L_d$ in different projection types across brain areas and layers. z and p-value are the z statistics and p-value of the marginal mean linear trends estimated from the fitted GLMMs. adjusted p-value is the adjusted p value through the BH multicomparison correction procedure.

Projection type	Coefficient	adjusted p-value	p-value	# of presynaptic neurons	# of postsynaptic neurons	# of ADP control neurons	# of same region control neurons	# of synapses	# of pre-post pairs	# of pre-ADP pairs	# of pre-'same region' pairs
$V1L2/3 \to V1L2/3$	0.020	2.56e-01	4.26e-02	20	604	2670	0	792	736	21247	0
$V1L2/3 \to V1L4$	0.050	5.19e-02	2.16e-03	19	197	2090	0	235	219	14010	0
$V1L2/3 \to V1L5$	0.012	5.26e-01	2.27e-01	20	311	1185	0	687	539	12384	0
$V1L2/3 \to HVAL2/3$	0.008	8.34e-01	6.26e-01	15	176	1169	0	208	196	4806	0
$V1L2/3 \to HVAL4$	0.014	8.18e-01	5.11e-01	14	80	856	0	89	82	3284	0
$V1L2/3 \to HVAL5$	0.001	9.50e-01	9.50e-01	13	91	429	0	131	106	2550	0
$V1L4 \to V1L2/3$	0.020	8.10e-01	4.59e-01	6	108	1784	0	120	110	3217	0
$V1L4 \to V1L4$	0.060	6.82e-02	5.69e-03	6	141	1865	0	155	146	4649	0
$V1L4 \to V1L5$	0.042	3.98e-01	8.30e-02	6	101	1138	0	130	110	3475	0
$V1L5 \to V1L4$	-0.016	8.34e-01	6.22e-01	6	64	1769	0	65	64	4044	0
$V1L5 \to V1L5$	0.034	5.26e-01	2.02e-01	6	103	1145	0	121	104	3825	0
$HVAL2/3 \to V1L2/3$	-0.015	5.26e-01	2.19e-01	36	381	2626	0	436	411	13081	0
$HVAL2/3 \to V1L4$	0.004	9.39e-01	8.88e-01	28	79	1882	0	88	81	5203	0
$HVAL2/3 \to V1L5$	-0.020	5.26e-01	1.37e-01	59	213	1172	0	324	278	13244	0
$HVAL2/3 \to HVAL2/3$	0.004	8.49e-01	6.74e-01	45	519	1264	0	801	732	19926	0
$HVAL2/3 \to HVAL4$	-0.002	9.39e-01	8.99e-01	38	204	893	0	301	258	13584	0
$HVAL2/3 \to HVAL5$	-0.004	8.49e-01	7.08e-01	62	216	439	0	410	361	13812	0
$HVAL4 \to HVAL2/3$	0.013	5.26e-01	2.41e-01	12	259	1233	0	334	316	6215	0
$HVAL4 \to HVAL4$	0.014	6.51e-01	3.26e-01	12	138	893	0	174	155	5421	0
$HVAL4 \to HVAL5$	0.048	1.25e-01	1.57e-02	11	89	434	0	108	97	3089	0
$HVAL5 \to V1L5$	0.016	8.34e-01	5.74e-01	14	59	1093	0	67	62	3601	0
$HVAL5 \to HVAL2/3$	0.008	8.10e-01	4.72e-01	17	260	1236	0	331	308	7872	0
$HVAL5 \to HVAL4$	0.021	5.26e-01	2.11e-01	17	92	896	0	110	102	6212	0
$HVAL5 \to HVAL5$	-0.004	8.78e-01	7.69e-01	19	148	439	0	214	196	5586	0

bioRxiv preprint doi: https://doi.org/10.1101/2023.03.13.531369; this version posted October 15, 2024. The copyright holder for this preprint (which was not certified by peer review) is the author/funder, who has granted bioRxiv a license to display the preprint in perpetuity. It is made available under aCC-BY 4.0 International license.

Supplemental Table 31. Estimated marginal means of linear trends for the effect of in silico ΔOri on L_d / neuron pair (synapses excluded) in different projection types across brain areas and layers. z and p-value are the z statistics and p-value of the marginal mean linear trends estimated from the fitted GLMMs. adjusted p-value is the adjusted p value through the BH multicomparison correction procedure.

Projection type	Coefficient	adjusted p-value	p-value	# of presynaptic neurons	# of postsynaptic neurons	# of ADP control neurons	# of same region control neurons	# of synapses	# of pre-post pairs	# of pre-ADP pairs	# of pre-'same region' pairs
$V1L2/3 \rightarrow V1L2/3$	-0.003	1.22e-10	7.61e-12	12	0	1409	12	0	0	6927	14702
$V1L2/3 \rightarrow V1L4$	-0.004	3.52e-07	4.40e-08	9	0	1050	9	0	0	4190	7845
$V1L2/3 \rightarrow V1L5$	-0.001	5.15e-01	3.22e-01	12	0	701	12	0	0	4527	4791
$V1L2/3 \rightarrow HVAL2/3$	-0.006	3.69e-06	6.93e-07	7	0	487	7	0	0	1354	3258
$V1L2/3 \rightarrow HVAL5$	-0.003	9.32e-02	4.21e-02	8	0	224	8	0	0	786	1249
$HVAL2/3 \rightarrow V1L2/3$	0.003	4.97e-04	1.24e-04	20	0	1299	20	0	0	4401	31930
$HVAL2/3 \rightarrow V1L4$	-0.000	9.42e-01	9.42e-01	16	0	885	16	0	0	1706	19798
$HVAL2/3 \rightarrow V1L5$	0.001	9.32e-02	4.66e-02	30	0	689	30	0	0	4433	19345
$HVAL2/3 \rightarrow HVAL2/3$	0.000	8.54e-01	7.47e-01	17	0	557	17	0	0	4103	7038
$HVAL2/3 \rightarrow HVAL4$	0.000	8.41e-01	6.83e-01	16	0	397	16	0	0	2812	4472
$HVAL2/3 \rightarrow HVAL5$	-0.001	4.10e-01	2.31e-01	27	0	229	27	0	0	3420	3482
$HVAL4 \rightarrow HVAL2/3$	-0.001	6.16e-01	4.24e-01	7	0	499	7	0	0	1262	3344
$HVAL4 \rightarrow HVAL5$	-0.000	8.41e-01	6.74e-01	7	0	225	7	0	0	937	848
$HVAL5 \rightarrow V1L5$	0.000	9.42e-01	9.22e-01	9	0	603	9	0	0	1414	5725
$HVAL5 \rightarrow HVAL2/3$	0.002	2.94e-02	9.20e-03	12	0	541	12	0	0	2307	5594
$HVAL5 \rightarrow HVAL5$	-0.002	9.32e-02	4.49e-02	14	0	228	14	0	0	2125	1430

bioRxiv preprint doi: https://doi.org/10.1101/2023.03.13.531369; this version posted October 15, 2024. The copyright holder for this preprint (which was not certified by peer review) is the author/funder, who has granted bioRxiv a license to display the preprint in perpetuity. It is made available under aCC-BY 4.0 International license.

Supplemental Table 32. Estimated marginal means of linear trends for the effect of in silico ΔOri on $N_{syn}/mm\ L_d$ in different projection types across brain areas and layers. z and p-value are the z statistics and p-value of the marginal mean linear trends estimated from the fitted GLMMs. adjusted p-value is the adjusted p value through the BH multicomparison correction procedure.

Projection type	Coefficient	adjusted p-value	p-value	# of presynaptic neurons	# of postsynaptic neurons	# of ADP control neurons	# of same region control neurons	# of synapses	# of pre-post pairs	# of pre-ADP pairs	# of pre-'same region' pairs
$V1L2/3 \rightarrow V1L2/3$	-0.003	4.73e-01	1.80e-01	12	246	1409	0	296	272	7199	0
$V1L2/3 \rightarrow V1L4$	0.005	4.73e-01	1.82e-01	9	82	1050	0	91	83	4273	0
$V1L2/3 \rightarrow V1L5$	-0.009	1.66e-03	1.04e-04	12	165	701	0	323	245	4772	0
$V1L2/3 \rightarrow HVAL2/3$	0.001	8.89e-01	8.89e-01	7	52	487	0	54	53	1407	0
$V1L2/3 \rightarrow HVAL5$	-0.014	8.07e-02	1.01e-02	8	38	224	0	51	40	826	0
$HVAL2/3 \rightarrow V1L2/3$	-0.004	4.73e-01	2.37e-01	20	123	1299	0	134	129	4530	0
$HVAL2/3 \rightarrow V1L4$	-0.012	4.04e-01	7.57e-02	16	33	885	0	36	33	1739	0
$HVAL2/3 \rightarrow V1L5$	-0.002	7.20e-01	6.06e-01	30	97	689	0	123	109	4542	0
$HVAL2/3 \rightarrow HVAL2/3$	-0.001	7.20e-01	6.07e-01	17	145	557	0	192	177	4280	0
$HVAL2/3 \rightarrow HVAL4$	0.002	7.31e-01	6.85e-01	16	59	397	0	70	67	2879	0
$HVAL2/3 \rightarrow HVAL5$	0.004	4.73e-01	2.07e-01	27	80	229	0	122	106	3526	0
$HVAL4 \rightarrow HVAL2/3$	0.005	5.95e-01	3.35e-01	7	55	499	0	59	59	1321	0
$HVAL4 \rightarrow HVAL5$	-0.009	4.73e-01	1.49e-01	7	29	225	0	32	30	967	0
$HVAL5 \rightarrow V1L5$	-0.003	7.20e-01	6.30e-01	9	25	603	0	31	26	1440	0
$HVAL5 \rightarrow HVAL2/3$	-0.002	7.20e-01	5.38e-01	12	87	541	0	106	98	2405	0
$HVAL5 \rightarrow HVAL5$	-0.004	6.13e-01	3.83e-01	14	56	228	0	71	67	2192	0

bioRxiv preprint doi: https://doi.org/10.1101/2023.03.13.531369; this version posted October 15, 2024. The copyright holder for this preprint (which was not certified by peer review) is the author/funder, who has granted bioRxiv a license to display the preprint in perpetuity. It is made available under aCC-BY 4.0 International license.

Supplemental Table 33. Paired t-tests for comparing the mean presyn-postsyn functional similarity between observation in the MICrONS dataset and values expected by GLMMs fit on the MICrONS dataset

Projection type	Comparison	t-statistic	p-value	adjusted p-value
$HVA \to HVA$	observed vs expected	660.0	7.92e-01	9.81e-01
$HVA \to V1$	observed vs expected	362.0	9.09e-01	9.81e-01
$V1 \to HVA$	observed vs expected	62.0	5.17e-01	9.81e-01
$V1 \to V1$	observed vs expected	313.0	9.81e-01	9.81e-01
$HVA \to HVA$	observed vs expected (synaptic scale)	675.0	8.99e-01	9.81e-01
$HVA \to V1$	observed vs expected (synaptic scale)	349.0	7.63e-01	9.81e-01
$V1 \to HVA$	observed vs expected (synaptic scale)	71.0	8.18e-01	9.81e-01
$V1 \to V1$	observed vs expected (synaptic scale)	280.0	5.76e-01	9.81e-01
$HVA \to HVA$	observed vs expected (axonal scale)	629.0	5.85e-01	9.81e-01
$HVA \to V1$	observed vs expected (axonal scale)	349.0	7.63e-01	9.81e-01
$V1 \to HVA$	observed vs expected (axonal scale)	63.0	5.48e-01	9.81e-01
$V1 \to V1$	observed vs expected (axonal scale)	284.0	6.22e-01	9.81e-01

bioRxiv preprint doi: https://doi.org/10.1101/2023.03.13.531369; this version posted October 15, 2024. The copyright holder for this preprint (which was not certified by peer review) is the author/funder, who has granted bioRxiv a license to display the preprint in perpetuity. It is made available under aCC-BY 4.0 International license.

Supplemental Table 34. Paired t-tests for comparing the mean postsyn-postsyn functional similarity between observation in the MICrONS dataset and values expected by GLMMs fit on the MICrONS dataset

Projection type	Comparison	t-statistic	p-value	adjusted p-value
$HVA \rightarrow HVA$	observed vs expected	254.0	7.45e-05	1.79e-04
$HVA \rightarrow V1$	observed vs expected	46.0	1.39e-07	5.56e-07
$V1 \rightarrow HVA$	observed vs expected	53.0	2.84e-01	2.84e-01
$V1 \rightarrow V1$	observed vs expected	45.0	9.74e-07	2.92e-06
$HVA \rightarrow HVA$	observed vs expected (synaptic scale)	344.0	1.68e-03	2.52e-03
$HVA \rightarrow V1$	observed vs expected (synaptic scale)	125.0	2.00e-04	3.99e-04
$V1 \rightarrow HVA$	observed vs expected (synaptic scale)	32.0	3.48e-02	4.64e-02
$V1 \rightarrow V1$	observed vs expected (synaptic scale)	197.0	5.35e-02	6.42e-02
$HVA \rightarrow HVA$	observed vs expected (axonal scale)	344.0	1.68e-03	2.52e-03
$HVA \rightarrow V1$	observed vs expected (axonal scale)	19.0	2.23e-09	1.34e-08
$V1 \rightarrow HVA$	observed vs expected (axonal scale)	53.0	2.84e-01	2.84e-01
$V1 \rightarrow V1$	observed vs expected (axonal scale)	5.0	5.82e-10	6.98e-09
