# Supplementary material for: Functional connectomics reveals general wiring rule in mouse visual cortex
Source: bioRxiv. 2023 Mar 30:2023.03.13.531369. Originally published 2023 Mar 14. Preprint. [Version 2] doi: 10.1101/2023.03.13.531369 (PMC10054929; doi:10.1101/2023.03.13.531369)
Supplement: Supplementary file 1 [file NIHPP2023.03.13.531369v2-supplement-1.pdf]

bioRxiv preprint doi: https://doi.org/10.1101/2023.03.13.531369; this version posted March 30, 2023. The copyright holder for this preprint (which was not certified by peer review) is the author/funder, who has granted bioRxiv a license to display the preprint in perpetuity. It is made available under aCC-BY 4.0 International license.

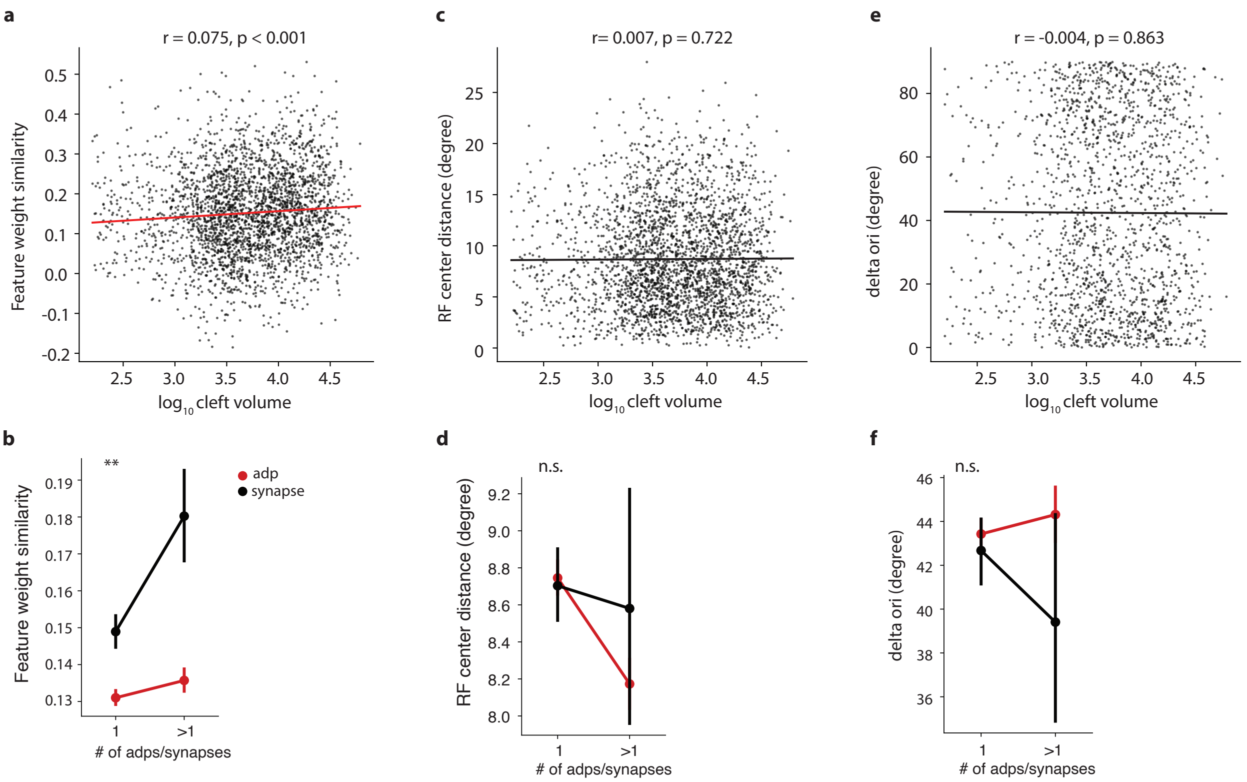

Supplemental Figure 1. Functional similarity predicts synaptic volume and number. a, c, e, Presynaptic-postsynaptic pairwise feature weight similarity (**a**), receptive field center distance (**c**), and difference in preferred orientation (**e**) as a function of synapse size (log_{10} cleft volume in voxels, r = pearson correlation coefficient, two sided p-value). **b, d, f**, Mean presynaptic-postsynaptic pairwise feature weight similarity (**b**), receptive field center distance (**d**), and difference in preferred orientation (**f**) for pairs with single versus multiple synapses (black) or ADPs (red). p-value by two way ANOVA.

bioRxiv preprint doi: https://doi.org/10.1101/2023.03.13.531369; this version posted March 30, 2023. The copyright holder for this preprint (which was not certified by peer review) is the author/funder, who has granted bioRxiv a license to display the preprint in perpetuity. It is made available under aCC-BY 4.0 International license.

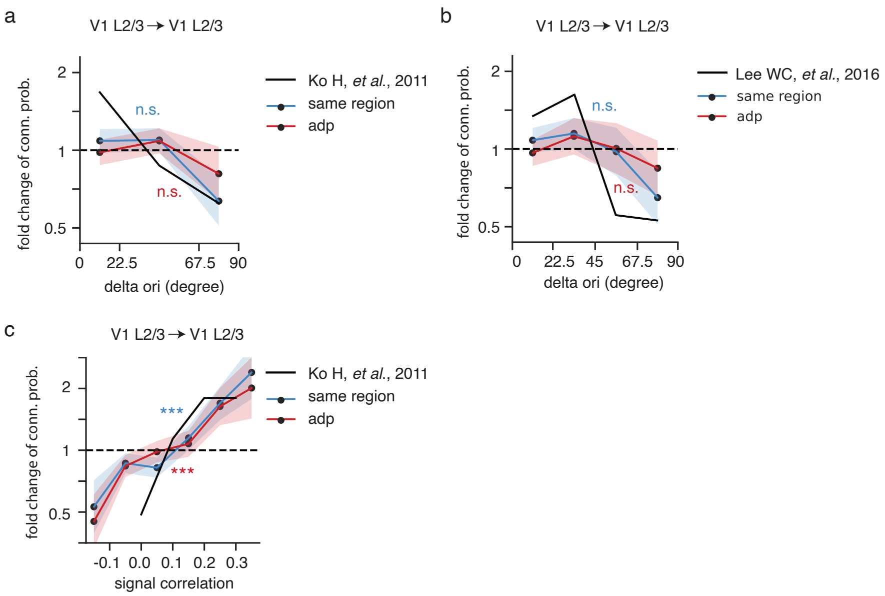

Supplemental Figure 2. Comparison of V1 L2/3 like-to-like findings to Ko et al. 2011 and Lee et al. 2016. a, As in Fig. 3i, but all presynaptic, postsynaptic, and control neurons restricted to V1 L2/3 (red,blue). Ko et al. 2011 data (black) and binning from Ko et al. 2011 Fig. 2b. **b**, As in **a**, but Lee et al. 2016 data (black) and binning from Lee et al. 2016 Fig. 2b. **c**, As in Fig. 2g, but all presynaptic, postsynaptic, and control neurons restricted to V1 L2/3 (red, blue). Ko et al. 2011 data (black) and binning from Ko et al. 2011 Fig. 3c.

bioRxiv preprint doi: https://doi.org/10.1101/2023.03.13.531369; this version posted March 30, 2023. The copyright holder for this preprint (which was not certified by peer review) is the author/funder, who has granted bioRxiv a license to display the preprint in perpetuity. It is made available under aCC-BY 4.0 International license.

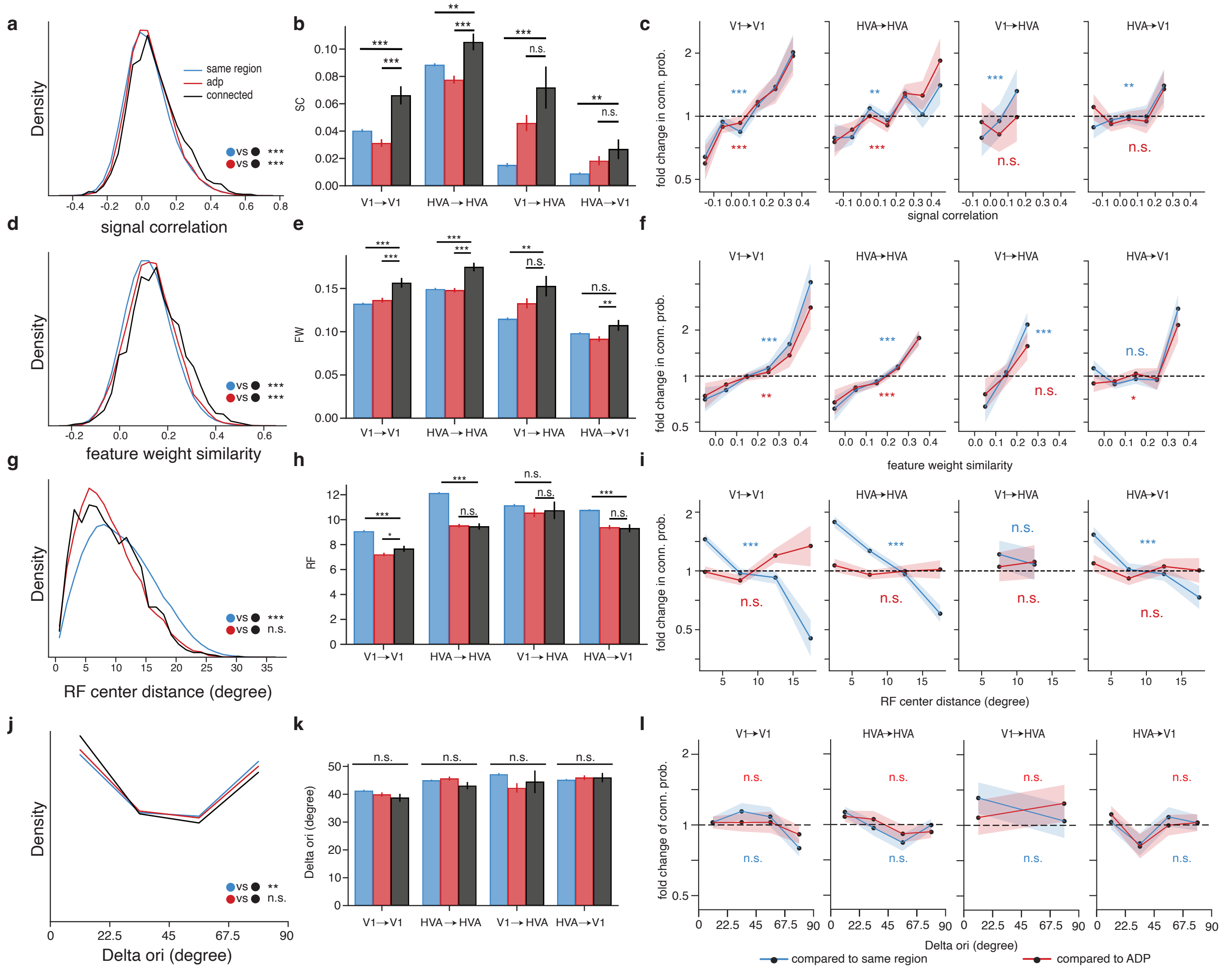

Supplemental Figure 3. Functional similarity selectivity findings consistent in orientation-tuned subsample. a, b, c, Selectivity with respect to signal correlation as Fig. 2c, e, g, but restricted to orientation-tuned neurons as in Fig. 3g, h, i. **d, e, f**, Same as **a, b, c**, but with respect to feature weight similarity as in Fig. 3a, b, c. **g, h, i**, Same as **a, b, c**, but with respect to receptive field center distance as in Fig. 3d, e, f. **j, k, l**, Same as Fig. 3g, h, i, duplicated here for reference.

bioRxiv preprint doi: https://doi.org/10.1101/2023.03.13.531369; this version posted March 30, 2023. The copyright holder for this preprint (which was not certified by peer review) is the author/funder, who has granted bioRxiv a license to display the preprint in perpetuity. It is made available under aCC-BY 4.0 International license.

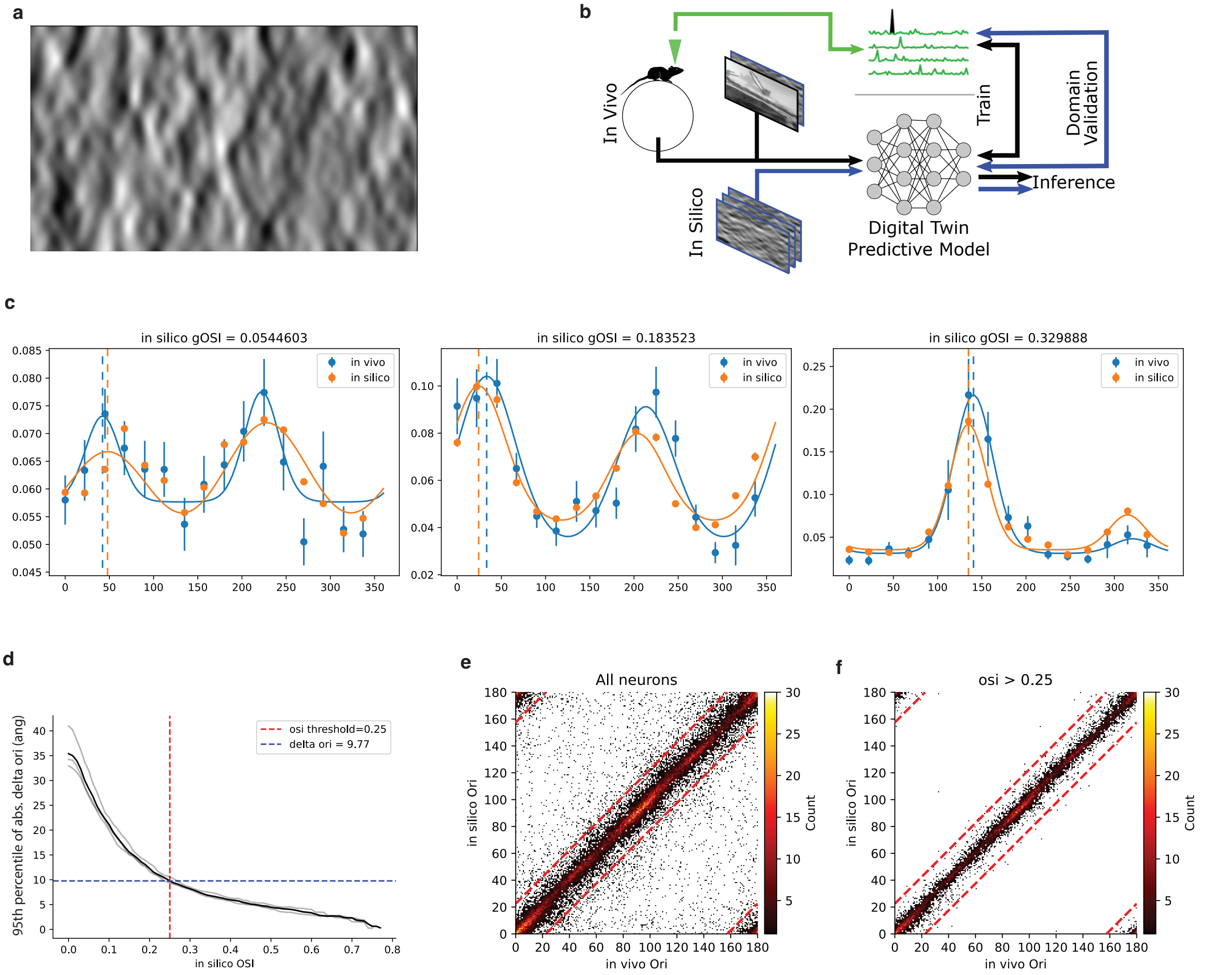

Supplemental Figure 4. In silico orientation tuning is consistent with in vivo orientation tuning a, Sample frame from global directional parametric stimulus ("Monet") used to characterize orientation and direction selectivity. Directional motion was orthogonal to orientation, and was tested at 22.5°intervals. **b**, Schematic of domain validation experimental design. In a single scan in a new animal, neuronal responses are collected in response to sufficient stimulus to both train the digital twin model and characterize orientation tuning from *in vivo* responses. Later, *in silico* orientation tuning is extracted from model responses to parametric stimuli, and compared against *in vivo* orientation tuning for the same neurons. **c**, Comparison of *in silico* and *in vivo* mean responses per stimulus direction (mean $\pm$ SEM), fitted tuning curves (lines), and extracted preferred orientation (dotted lines) for three neurons. **d**, 95th percentile difference in preferred orientation between *in silico* and *in vivo* fitted responses as a function of gOSI threshold. Dotted lines correspond to gOSI > 0.25 threshold applied for all analyses and resulting 95th percentile difference in preferred orientation $\approx 9.77°$ across all three animals imaged. Lines correspond to individual animals (gray) or cumulative across all animals (black). **e, f**, Two-dimensional histogram of *in silico* versus *in vivo* preferred orientation for all neurons across three animals (**e**) and only neurons with gOSI > 0.25 (**f**).

bioRxiv preprint doi: https://doi.org/10.1101/2023.03.13.531369; this version posted March 30, 2023. The copyright holder for this preprint (which was not certified by peer review) is the author/funder, who has granted bioRxiv a license to display the preprint in perpetuity. It is made available under aCC-BY 4.0 International license.

Supplemental Table 1. Independent two-sided t-tests performed to compare signal correlation between connected neuron pairs and control neuron pairs across brain areas
connected pair #, **connected pre #** and **connected post #** are the number of neuron pairs, the number of unique presynaptic neurons and the number of unique postsynaptic neurons included in the connected neuron pairs respectively. **control pair #**, **control pre #** and **control post #** are the number of neuron pairs, the number of unique presynaptic neurons and the number of unique postsynaptic neurons included in the control neuron pairs respectively. **t** and **p** are the test statistic and p value of the independent two-sample two-sided t tests. **adj. p** is the adjusted p value through the BH multicomparison correction procedure. **dof** is the degree of freedom of the t tests.

projection group	control	connected			control			t	p	adj. p	dof
		pair #	pre #	post #	pair #	pre #	post #				
V1->V1	adp	929	32	692	5136	32	1465	9.14	8.35e-20	6.68e-19	6063
HVA->HVA	adp	1260	86	680	7148	75	867	4.45	8.62e-06	1.38e-05	8406
V1->HVA	adp	191	22	174	1117	20	626	3.22	1.30e-03	1.49e-03	1306
HVA->V1	adp	456	62	399	2248	59	1171	4.24	2.33e-05	3.11e-05	2702
V1->V1	same region	929	32	692	62095	32	2131	8.67	4.35e-18	1.74e-17	63022
HVA->HVA	same region	1260	86	680	87826	86	1120	2.60	9.44e-03	9.44e-03	89084
V1->HVA	same region	191	22	174	23332	22	1120	5.28	1.32e-07	2.63e-07	23521
HVA->V1	same region	456	62	399	129418	62	2131	6.00	1.95e-09	5.20e-09	129872

bioRxiv preprint doi: https://doi.org/10.1101/2023.03.13.531369; this version posted March 30, 2023. The copyright holder for this preprint (which was not certified by peer review) is the author/funder, who has granted bioRxiv a license to display the preprint in perpetuity. It is made available under aCC-BY 4.0 International license.

Supplemental Table 2. Independent two-sided t-tests performed to compare signal correlation between connected neuron pairs and control neuron pairs across brain areas and layers

connected pair #, **connected pre #** and **connected post #** are the number of neuron pairs, the number of unique presynaptic neurons and the number of unique postsynaptic neurons included in the connected neuron pairs respectively. **control pair #**, **control pre #** and **control post #** are the number of neuron pairs, the number of unique presynaptic neurons and the number of unique postsynaptic neurons included in the control neuron pairs respectively. **t** and **p** are the test statistic and p value of the independent two-sample two-sided t tests. **adj. p** is the adjusted p value through the BH multicomparison correction procedure. **dof** is the degree of freedom of the t tests.

projection group	control	connected			control			t	p	adj. p	dof
		pair #	pre #	post #	pair #	pre #	post #				
V1, L2/3->V1, L2/3	adp	227	19	181	1023	19	447	5.13	3.40e-07	4.93e-06	1248
V1, L2/3->V1, L4	adp	112	17	103	353	16	215	1.99	4.68e-02	1.04e-01	463
V1, L2/3->V1, L5	adp	269	19	194	1161	18	460	5.98	2.78e-09	8.07e-08	1428
V1, L2/3->HVA, L2/3	adp	68	11	62	321	11	214	0.54	5.92e-01	7.44e-01	387
V1, L2/3->HVA, L4	adp	28	9	27	63	8	60	0.43	6.70e-01	7.93e-01	89
V1, L2/3->HVA, L5	adp	68	14	65	317	14	192	2.81	5.26e-03	2.18e-02	383
V1, L4->V1, L2/3	adp	12	1	12	84	1	84	-0.07	9.47e-01	9.63e-01	94
V1, L4->V1, L4	adp	11	1	11	27	1	27	0.79	4.37e-01	6.07e-01	36
V1, L4->V1, L5	adp	12	1	12	29	1	29	0.40	6.92e-01	8.03e-01	39
V1, L5->V1, L2/3	adp	72	9	67	303	8	249	0.70	4.85e-01	6.40e-01	373
V1, L5->V1, L4	adp	46	10	46	113	9	104	1.29	2.00e-01	3.38e-01	157
V1, L5->V1, L5	adp	119	10	110	497	10	341	3.81	1.51e-04	1.09e-03	614
V1, L5->HVA, L2/3	adp	13	2	13	89	1	89	2.19	3.09e-02	8.14e-02	100
HVA, L2/3->V1, L2/3	adp	147	27	138	593	24	377	2.13	3.31e-02	8.36e-02	738
HVA, L2/3->V1, L4	adp	23	11	23	70	9	64	0.01	9.89e-01	9.89e-01	91
HVA, L2/3->V1, L5	adp	129	39	109	503	37	318	3.63	3.10e-04	1.80e-03	630
HVA, L2/3->HVA, L2/3	adp	296	46	207	1266	26	335	0.94	3.47e-01	5.17e-01	1560
HVA, L2/3->HVA, L4	adp	93	27	77	276	22	150	2.66	8.11e-03	3.14e-02	367
HVA, L2/3->HVA, L5	adp	226	44	146	1111	41	272	3.57	3.75e-04	1.98e-03	1335
HVA, L4->V1, L5	adp	12	3	12	52	3	51	0.09	9.30e-01	9.63e-01	62
HVA, L4->HVA, L2/3	adp	116	9	99	531	8	267	-0.09	9.27e-01	9.63e-01	645
HVA, L4->HVA, L4	adp	61	10	56	171	9	121	-0.50	6.16e-01	7.45e-01	230
HVA, L4->HVA, L5	adp	77	9	69	312	9	172	2.60	9.80e-03	3.34e-02	387
HVA, L5->V1, L2/3	adp	72	8	71	301	7	267	0.77	4.39e-01	6.07e-01	371
HVA, L5->V1, L4	adp	19	6	19	43	5	43	0.86	3.94e-01	5.71e-01	60
HVA, L5->V1, L5	adp	47	14	44	148	13	126	-1.27	2.04e-01	3.38e-01	193
HVA, L5->HVA, L2/3	adp	159	17	136	699	17	290	1.23	2.21e-01	3.56e-01	856
HVA, L5->HVA, L4	adp	60	18	50	146	17	111	1.42	1.58e-01	2.92e-01	204
HVA, L5->HVA, L5	adp	172	20	129	748	19	254	1.02	3.06e-01	4.67e-01	918
V1, L2/3->V1, L2/3	same region	227	19	181	15812	19	899	5.29	1.26e-07	2.44e-06	16037
V1, L2/3->V1, L4	same region	112	17	103	8120	17	505	2.03	4.25e-02	9.86e-02	8230
V1, L2/3->V1, L5	same region	269	19	194	12060	19	710	6.72	1.90e-11	1.10e-09	12327
V1, L2/3->HVA, L2/3	same region	68	11	62	4627	11	456	1.36	1.74e-01	3.06e-01	4693
V1, L2/3->HVA, L4	same region	28	9	27	2564	9	295	1.84	6.56e-02	1.41e-01	2590
V1, L2/3->HVA, L5	same region	68	14	65	4781	14	369	5.04	4.89e-07	5.68e-06	4847
V1, L4->V1, L2/3	same region	12	1	12	803	1	803	-0.52	6.03e-01	7.44e-01	813
V1, L4->V1, L4	same region	11	1	11	466	1	466	1.56	1.18e-01	2.29e-01	475
V1, L4->V1, L5	same region	12	1	12	669	1	669	2.35	1.90e-02	5.24e-02	679
V1, L5->V1, L2/3	same region	72	9	67	7716	9	899	-0.54	5.91e-01	7.44e-01	7786
V1, L5->V1, L4	same region	46	10	46	4891	10	505	1.40	1.61e-01	2.92e-01	4935
V1, L5->V1, L5	same region	119	10	110	6474	10	710	4.88	1.07e-06	1.04e-05	6591
V1, L5->HVA, L2/3	same region	13	2	13	810	2	456	-2.07	3.86e-02	9.33e-02	821
HVA, L2/3->V1, L2/3	same region	147	27	138	23533	27	899	2.58	9.78e-03	3.34e-02	23678
HVA, L2/3->V1, L4	same region	23	11	23	5462	11	505	0.75	4.52e-01	6.10e-01	5483
HVA, L2/3->V1, L5	same region	129	39	109	27058	39	710	3.82	1.36e-04	1.09e-03	27185
HVA, L2/3->HVA, L2/3	same region	296	46	207	19368	46	456	0.12	9.05e-01	9.63e-01	19662
HVA, L2/3->HVA, L4	same region	93	27	77	7596	27	295	1.76	7.76e-02	1.61e-01	7687
HVA, L2/3->HVA, L5	same region	226	44	146	14899	44	369	3.36	7.74e-04	3.74e-03	15123
HVA, L4->V1, L5	same region	12	3	12	2066	3	710	2.38	1.76e-02	5.24e-02	2076
HVA, L4->HVA, L2/3	same region	116	9	99	3457	9	456	3.25	1.18e-03	5.27e-03	3571
HVA, L4->HVA, L4	same region	61	10	56	2708	10	295	0.07	9.45e-01	9.63e-01	2767
HVA, L4->HVA, L5	same region	77	9	69	2932	9	369	3.62	3.01e-04	1.80e-03	3007
HVA, L5->V1, L2/3	same region	72	8	71	6819	8	899	1.18	2.40e-01	3.76e-01	6889
HVA, L5->V1, L4	same region	19	6	19	2968	6	505	1.73	8.34e-02	1.67e-01	2985
HVA, L5->V1, L5	same region	47	14	44	9745	14	710	0.31	7.53e-01	8.40e-01	9790
HVA, L5->HVA, L2/3	same region	159	17	136	6894	17	456	2.35	1.90e-02	5.24e-02	7051
HVA, L5->HVA, L4	same region	60	18	50	5104	18	295	2.54	1.11e-02	3.59e-02	5162

Continued on next page

bioRxiv preprint doi: https://doi.org/10.1101/2023.03.13.531369; this version posted March 30, 2023. The copyright holder for this preprint (which was not certified by peer review) is the author/funder, who has granted bioRxiv a license to display the preprint in perpetuity. It is made available under aCC-BY 4.0 International license.

Supplemental Table 2. Independent two-sided t-tests performed to compare signal correlation between connected neuron pairs and control neuron pairs across brain areas and layers
connected pair #, **connected pre #** and **connected post #** are the number of neuron pairs, the number of unique presynaptic neurons and the number of unique postsynaptic neurons included in the connected neuron pairs respectively. **control pair #**, **control pre #** and **control post #** are the number of neuron pairs, the number of unique presynaptic neurons and the number of unique postsynaptic neurons included in the control neuron pairs respectively. **t** and **p** are the test statistic and p value of the independent two-sample two-sided t tests. **adj. p** is the adjusted p value through the BH multicomparison correction procedure. **dof** is the degree of freedom of the t tests.

projection group	control	connected			control			t	p	adj. p	dof
		pair #	pre #	post #	pair #	pre #	post #				
HVA, L5->HVA, L5	same region	172	20	129	6440	20	369	-0.35	7.29e-01	8.29e-01	6610

bioRxiv preprint doi: https://doi.org/10.1101/2023.03.13.531369; this version posted March 30, 2023. The copyright holder for this preprint (which was not certified by peer review) is the author/funder, who has granted bioRxiv a license to display the preprint in perpetuity. It is made available under aCC-BY 4.0 International license.

Supplemental Table 3. Two-sided Cochran-Armitage test of trend performed to test whether connection probability increases with respect to signal correlation
statistic and **p** are the test statistic and p value of the two-sided Cochran-Armitage test of trend. **adj. p** is the adjusted p value through the BH multicomparison correction procedure.

projection group	control	statistic	p	adj. p
V1->V1	adp	23235	2.27e-14	9.08e-14
HVA->HVA	adp	37686	1.07e-04	2.13e-04
V1->HVA	adp	5149	1.43e-02	1.43e-02
HVA->V1	adp	10037	1.37e-03	1.82e-03
V1->V1	same region	305178	1.03e-17	4.14e-17
HVA->HVA	same region	493604	8.08e-03	8.08e-03
V1->HVA	same region	110311	1.51e-07	2.01e-07
HVA->V1	same region	596230	5.45e-08	1.09e-07

bioRxiv preprint doi: https://doi.org/10.1101/2023.03.13.531369; this version posted March 30, 2023. The copyright holder for this preprint (which was not certified by peer review) is the author/funder, who has granted bioRxiv a license to display the preprint in perpetuity. It is made available under aCC-BY 4.0 International license.

Supplemental Table 4. Independent two-sided t-tests performed to compare feature weight similarity between connected neuron pairs and control neuron pairs across brain areas

connected pair #, **connected pre #** and **connected post #** are the number of neuron pairs, the number of unique presynaptic neurons and the number of unique postsynaptic neurons included in the connected neuron pairs respectively. **control pair #**, **control pre #** and **control post #** are the number of neuron pairs, the number of unique presynaptic neurons and the number of unique postsynaptic neurons included in the control neuron pairs respectively. **t** and **p** are the test statistic and p value of the independent two-sample two-sided t tests. **adj. p** is the adjusted p value through the BH multicomparison correction procedure. **dof** is the degree of freedom of the t tests.

projection group	control	connected			control			t	p	adj. p	dof
		pair #	pre #	post #	pair #	pre #	post #				
V1->V1	adp	929	32	692	5136	32	1465	6.71	2.14e-11	8.58e-11	6063
HVA->HVA	adp	1260	86	680	7148	75	867	5.27	1.41e-07	2.25e-07	8406
V1->HVA	adp	191	22	174	1117	20	626	3.24	1.24e-03	1.24e-03	1306
HVA->V1	adp	456	62	399	2248	59	1171	4.14	3.64e-05	4.85e-05	2702
V1->V1	same region	929	32	692	62095	32	2131	7.80	6.41e-15	5.13e-14	63022
HVA->HVA	same region	1260	86	680	87826	86	1120	5.47	4.51e-08	1.20e-07	89084
V1->HVA	same region	191	22	174	23332	22	1120	3.91	9.17e-05	1.05e-04	23521
HVA->V1	same region	456	62	399	129418	62	2131	5.37	7.78e-08	1.56e-07	129872

bioRxiv preprint doi: https://doi.org/10.1101/2023.03.13.531369; this version posted March 30, 2023. The copyright holder for this preprint (which was not certified by peer review) is the author/funder, who has granted bioRxiv a license to display the preprint in perpetuity. It is made available under aCC-BY 4.0 International license.

Supplemental Table 5. Independent two-sided t-tests performed to compare feature weight similarity between connected neuron pairs and control neuron pairs across brain areas and layers

connected pair #, **connected pre #** and **connected post #** are the number of neuron pairs, the number of unique presynaptic neurons and the number of unique postsynaptic neurons included in the connected neuron pairs respectively. **control pair #**, **control pre #** and **control post #** are the number of neuron pairs, the number of unique presynaptic neurons and the number of unique postsynaptic neurons included in the control neuron pairs respectively. **t** and **p** are the test statistic and p value of the independent two-sample two-sided t tests. **adj. p** is the adjusted p value through the BH multicomparison correction procedure. **dof** is the degree of freedom of the t tests.

projection group	control	connected			control			t	p	adj. p	dof
		pair #	pre #	post #	pair #	pre #	post #				
V1, L2/3->V1, L2/3	adp	227	19	181	1023	19	447	3.54	4.21e-04	2.44e-03	1248
V1, L2/3->V1, L4	adp	112	17	103	353	16	215	1.04	2.99e-01	4.95e-01	463
V1, L2/3->V1, L5	adp	269	19	194	1161	18	460	4.95	8.31e-07	2.41e-05	1428
V1, L2/3->HVA, L2/3	adp	68	11	62	321	11	214	0.68	4.99e-01	6.58e-01	387
V1, L2/3->HVA, L4	adp	28	9	27	63	8	60	1.13	2.60e-01	4.58e-01	89
V1, L2/3->HVA, L5	adp	68	14	65	317	14	192	3.09	2.16e-03	1.05e-02	383
V1, L4->V1, L2/3	adp	12	1	12	84	1	84	-0.94	3.52e-01	5.47e-01	94
V1, L4->V1, L4	adp	11	1	11	27	1	27	-0.10	9.20e-01	9.52e-01	36
V1, L4->V1, L5	adp	12	1	12	29	1	29	0.25	8.07e-01	8.90e-01	39
V1, L5->V1, L2/3	adp	72	9	67	303	8	249	-0.25	8.05e-01	8.90e-01	373
V1, L5->V1, L4	adp	46	10	46	113	9	104	0.24	8.13e-01	8.90e-01	157
V1, L5->V1, L5	adp	119	10	110	497	10	341	2.98	3.03e-03	1.35e-02	614
V1, L5->HVA, L2/3	adp	13	2	13	89	1	89	-0.18	8.56e-01	9.19e-01	100
HVA, L2/3->V1, L2/3	adp	147	27	138	593	24	377	2.30	2.17e-02	5.99e-02	738
HVA, L2/3->V1, L4	adp	23	11	23	70	9	64	-0.07	9.42e-01	9.58e-01	91
HVA, L2/3->V1, L5	adp	129	39	109	503	37	318	4.28	2.13e-05	1.76e-04	630
HVA, L2/3->HVA, L2/3	adp	296	46	207	1266	26	335	1.89	5.93e-02	1.56e-01	1560
HVA, L2/3->HVA, L4	adp	93	27	77	276	22	150	2.51	1.26e-02	3.84e-02	367
HVA, L2/3->HVA, L5	adp	226	44	146	1111	41	272	4.68	3.21e-06	6.20e-05	1335
HVA, L4->V1, L5	adp	12	3	12	52	3	51	0.34	7.37e-01	8.55e-01	62
HVA, L4->HVA, L2/3	adp	116	9	99	531	8	267	0.61	5.43e-01	6.84e-01	645
HVA, L4->HVA, L4	adp	61	10	56	171	9	121	0.69	4.91e-01	6.58e-01	230
HVA, L4->HVA, L5	adp	77	9	69	312	9	172	1.82	6.92e-02	1.67e-01	387
HVA, L5->V1, L2/3	adp	72	8	71	301	7	267	-0.14	8.86e-01	9.35e-01	371
HVA, L5->V1, L4	adp	19	6	19	43	5	43	1.37	1.76e-01	3.64e-01	60
HVA, L5->V1, L5	adp	47	14	44	148	13	126	-1.01	3.12e-01	5.03e-01	193
HVA, L5->HVA, L2/3	adp	159	17	136	699	17	290	0.35	7.29e-01	8.55e-01	856
HVA, L5->HVA, L4	adp	60	18	50	146	17	111	0.49	6.24e-01	7.54e-01	204
HVA, L5->HVA, L5	adp	172	20	129	748	19	254	1.33	1.85e-01	3.71e-01	918
V1, L2/3->V1, L2/3	same region	227	19	181	15812	19	899	4.52	6.11e-06	8.85e-05	16037
V1, L2/3->V1, L4	same region	112	17	103	8120	17	505	2.30	2.16e-02	5.99e-02	8230
V1, L2/3->V1, L5	same region	269	19	194	12060	19	710	5.31	1.12e-07	6.48e-06	12327
V1, L2/3->HVA, L2/3	same region	68	11	62	4627	11	456	1.82	6.86e-02	1.67e-01	4693
V1, L2/3->HVA, L4	same region	28	9	27	2564	9	295	0.72	4.74e-01	6.54e-01	2590
V1, L2/3->HVA, L5	same region	68	14	65	4781	14	369	4.42	1.00e-05	9.69e-05	4847
V1, L4->V1, L2/3	same region	12	1	12	803	1	803	-0.90	3.69e-01	5.49e-01	813
V1, L4->V1, L4	same region	11	1	11	466	1	466	1.10	2.72e-01	4.64e-01	475
V1, L4->V1, L5	same region	12	1	12	669	1	669	1.78	7.51e-02	1.74e-01	679
V1, L5->V1, L2/3	same region	72	9	67	7716	9	899	-1.61	1.08e-01	2.32e-01	7786
V1, L5->V1, L4	same region	46	10	46	4891	10	505	0.54	5.92e-01	7.31e-01	4935
V1, L5->V1, L5	same region	119	10	110	6474	10	710	4.11	4.00e-05	2.90e-04	6591
V1, L5->HVA, L2/3	same region	13	2	13	810	2	456	-3.22	1.33e-03	7.00e-03	821
HVA, L2/3->V1, L2/3	same region	147	27	138	23533	27	899	2.69	7.25e-03	2.63e-02	23678
HVA, L2/3->V1, L4	same region	23	11	23	5462	11	505	0.78	4.37e-01	6.19e-01	5483
HVA, L2/3->V1, L5	same region	129	39	109	27058	39	710	4.05	5.08e-05	3.27e-04	27185
HVA, L2/3->HVA, L2/3	same region	296	46	207	19368	46	456	1.20	2.31e-01	4.19e-01	19662
HVA, L2/3->HVA, L4	same region	93	27	77	7596	27	295	2.71	6.83e-03	2.63e-02	7687
HVA, L2/3->HVA, L5	same region	226	44	146	14899	44	369	4.46	8.18e-06	9.49e-05	15123
HVA, L4->V1, L5	same region	12	3	12	2066	3	710	2.80	5.10e-03	2.11e-02	2076
HVA, L4->HVA, L2/3	same region	116	9	99	3457	9	456	2.57	1.01e-02	3.27e-02	3571
HVA, L4->HVA, L4	same region	61	10	56	2708	10	295	0.92	3.58e-01	5.47e-01	2767
HVA, L4->HVA, L5	same region	77	9	69	2932	9	369	2.66	7.82e-03	2.67e-02	3007
HVA, L5->V1, L2/3	same region	72	8	71	6819	8	899	0.86	3.90e-01	5.65e-01	6889
HVA, L5->V1, L4	same region	19	6	19	2968	6	505	1.25	2.13e-01	4.12e-01	2985
HVA, L5->V1, L5	same region	47	14	44	9745	14	710	-0.04	9.67e-01	9.67e-01	9790
HVA, L5->HVA, L2/3	same region	159	17	136	6894	17	456	0.64	5.25e-01	6.77e-01	7051
HVA, L5->HVA, L4	same region	60	18	50	5104	18	295	1.21	2.26e-01	4.19e-01	5162

Continued on next page

bioRxiv preprint doi: https://doi.org/10.1101/2023.03.13.531369; this version posted March 30, 2023. The copyright holder for this preprint (which was not certified by peer review) is the author/funder, who has granted bioRxiv a license to display the preprint in perpetuity. It is made available under aCC-BY 4.0 International license.

Supplemental Table 5. Independent two-sided t-tests performed to compare feature weight similarity between connected neuron pairs and control neuron pairs across brain areas and layers

connected pair #, **connected pre #** and **connected post #** are the number of neuron pairs, the number of unique presynaptic neurons and the number of unique postsynaptic neurons included in the connected neuron pairs respectively. **control pair #**, **control pre #** and **control post #** are the number of neuron pairs, the number of unique presynaptic neurons and the number of unique postsynaptic neurons included in the control neuron pairs respectively. **t** and **p** are the test statistic and p value of the independent two-sample two-sided t tests. **adj. p** is the adjusted p value through the BH multicomparison correction procedure. **dof** is the degree of freedom of the t tests.

projection group	control	connected			control			t	p	adj. p	dof
		pair #	pre #	post #	pair #	pre #	post #				
HVA, L5->HVA, L5	same region	172	20	129	6440	20	369	1.71	8.75e-02	1.95e-01	6610

bioRxiv preprint doi: https://doi.org/10.1101/2023.03.13.531369; this version posted March 30, 2023. The copyright holder for this preprint (which was not certified by peer review) is the author/funder, who has granted bioRxiv a license to display the preprint in perpetuity. It is made available under aCC-BY 4.0 International license.

Supplemental Table 6. Two-sided Cochran-Armitage test of trend performed to test whether connection probability increases with respect to feature weight similarity

statistic and **p** are the test statistic and p value of the two-sided Cochran-Armitage test of trend. **adj. p** is the adjusted p value through the BH multicomparison correction procedure.

projection group	control	statistic	p	adj. p
V1->V1	adp	16891	8.98e-08	3.59e-07
HVA->HVA	adp	25625	3.32e-06	6.64e-06
V1->HVA	adp	4801	2.11e-02	2.11e-02
HVA->V1	adp	6713	2.85e-03	3.80e-03
V1->V1	same region	235787	7.03e-13	2.81e-12
HVA->HVA	same region	358809	2.21e-08	4.42e-08
V1->HVA	same region	84906	1.70e-04	1.70e-04
HVA->V1	same region	444800	3.29e-06	4.39e-06

bioRxiv preprint doi: https://doi.org/10.1101/2023.03.13.531369; this version posted March 30, 2023. The copyright holder for this preprint (which was not certified by peer review) is the author/funder, who has granted bioRxiv a license to display the preprint in perpetuity. It is made available under aCC-BY 4.0 International license.

Supplemental Table 7. Independent two-sided t-tests performed to compare receptive field center distance between connected neuron pairs and control neuron pairs across brain areas

connected pair #, **connected pre #** and **connected post #** are the number of neuron pairs, the number of unique presynaptic neurons and the number of unique postsynaptic neurons included in the connected neuron pairs respectively. **control pair #**, **control pre #** and **control post #** are the number of neuron pairs, the number of unique presynaptic neurons and the number of unique postsynaptic neurons included in the control neuron pairs respectively. **t** and **p** are the test statistic and p value of the independent two-sample two-sided t tests. **adj. p** is the adjusted p value through the BH multicomparison correction procedure. **dof** is the degree of freedom of the t tests.

projection group	control	connected			control			t	p	adj. p	dof
		pair #	pre #	post #	pair #	pre #	post #				
V1->V1	adp	929	32	692	5136	32	1465	2.10	3.61e-02	5.78e-02	6063
HVA->HVA	adp	1260	86	680	7148	75	867	-0.46	6.45e-01	7.37e-01	8406
V1->HVA	adp	191	22	174	1117	20	626	-0.31	7.58e-01	7.58e-01	1306
HVA->V1	adp	456	62	399	2248	59	1171	0.91	3.61e-01	4.82e-01	2702
V1->V1	same region	929	32	692	62095	32	2131	-9.07	1.22e-19	4.86e-19	63022
HVA->HVA	same region	1260	86	680	87826	86	1120	-14.72	5.86e-49	4.69e-48	89084
V1->HVA	same region	191	22	174	23332	22	1120	-3.34	8.42e-04	1.68e-03	23521
HVA->V1	same region	456	62	399	129418	62	2131	-5.37	7.69e-08	2.05e-07	129872

bioRxiv preprint doi: https://doi.org/10.1101/2023.03.13.531369; this version posted March 30, 2023. The copyright holder for this preprint (which was not certified by peer review) is the author/funder, who has granted bioRxiv a license to display the preprint in perpetuity. It is made available under aCC-BY 4.0 International license.

Supplemental Table 8. Independent two-sided t-tests performed to compare receptive field center distance between connected neuron pairs and control neuron pairs across brain areas and layers

connected pair #, **connected pre #** and **connected post #** are the number of neuron pairs, the number of unique presynaptic neurons and the number of unique postsynaptic neurons included in the connected neuron pairs respectively. **control pair #**, **control pre #** and **control post #** are the number of neuron pairs, the number of unique presynaptic neurons and the number of unique postsynaptic neurons included in the control neuron pairs respectively. **t** and **p** are the test statistic and p value of the independent two-sample two-sided t tests. **adj. p** is the adjusted p value through the BH multicomparison correction procedure. **dof** is the degree of freedom of the t tests.

projection group	control	connected			control			t	p	adj. p	dof
		pair #	pre #	post #	pair #	pre #	post #				
V1, L2/3->V1, L2/3	adp	227	19	181	1023	19	447	0.32	7.46e-01	8.61e-01	1248
V1, L2/3->V1, L4	adp	112	17	103	353	16	215	0.83	4.06e-01	5.75e-01	463
V1, L2/3->V1, L5	adp	269	19	194	1161	18	460	1.20	2.31e-01	3.93e-01	1428
V1, L2/3->HVA, L2/3	adp	68	11	62	321	11	214	-0.68	4.98e-01	6.71e-01	387
V1, L2/3->HVA, L4	adp	28	9	27	63	8	60	-0.94	3.50e-01	5.20e-01	89
V1, L2/3->HVA, L5	adp	68	14	65	317	14	192	0.24	8.07e-01	8.84e-01	383
V1, L4->V1, L2/3	adp	12	1	12	84	1	84	2.03	4.47e-02	1.30e-01	94
V1, L4->V1, L4	adp	11	1	11	27	1	27	0.25	8.08e-01	8.84e-01	36
V1, L4->V1, L5	adp	12	1	12	29	1	29	-1.06	2.97e-01	4.54e-01	39
V1, L5->V1, L2/3	adp	72	9	67	303	8	249	0.43	6.70e-01	8.44e-01	373
V1, L5->V1, L4	adp	46	10	46	113	9	104	0.57	5.70e-01	7.35e-01	157
V1, L5->V1, L5	adp	119	10	110	497	10	341	-0.16	8.70e-01	9.35e-01	614
V1, L5->HVA, L2/3	adp	13	2	13	89	1	89	-1.44	1.52e-01	3.04e-01	100
HVA, L2/3->V1, L2/3	adp	147	27	138	593	24	377	-0.91	3.65e-01	5.29e-01	738
HVA, L2/3->V1, L4	adp	23	11	23	70	9	64	0.57	5.70e-01	7.35e-01	91
HVA, L2/3->V1, L5	adp	129	39	109	503	37	318	0.01	9.90e-01	9.90e-01	630
HVA, L2/3->HVA, L2/3	adp	296	46	207	1266	26	335	-1.36	1.75e-01	3.28e-01	1560
HVA, L2/3->HVA, L4	adp	93	27	77	276	22	150	-1.42	1.57e-01	3.04e-01	367
HVA, L2/3->HVA, L5	adp	226	44	146	1111	41	272	-0.36	7.22e-01	8.61e-01	1335
HVA, L4->V1, L5	adp	12	3	12	52	3	51	0.34	7.32e-01	8.61e-01	62
HVA, L4->HVA, L2/3	adp	116	9	99	531	8	267	-0.37	7.08e-01	8.61e-01	645
HVA, L4->HVA, L4	adp	61	10	56	171	9	121	-1.49	1.39e-01	3.04e-01	230
HVA, L4->HVA, L5	adp	77	9	69	312	9	172	1.18	2.40e-01	3.99e-01	387
HVA, L5->V1, L2/3	adp	72	8	71	301	7	267	1.49	1.37e-01	3.04e-01	371
HVA, L5->V1, L4	adp	19	6	19	43	5	43	-2.01	4.85e-02	1.34e-01	60
HVA, L5->V1, L5	adp	47	14	44	148	13	126	-0.08	9.35e-01	9.64e-01	193
HVA, L5->HVA, L2/3	adp	159	17	136	699	17	290	1.34	1.81e-01	3.29e-01	856
HVA, L5->HVA, L4	adp	60	18	50	146	17	111	0.11	9.16e-01	9.64e-01	204
HVA, L5->HVA, L5	adp	172	20	129	748	19	254	1.25	2.11e-01	3.70e-01	918
V1, L2/3->V1, L2/3	same region	227	19	181	15812	19	899	-7.96	1.81e-15	1.05e-13	16037
V1, L2/3->V1, L4	same region	112	17	103	8120	17	505	-4.27	2.00e-05	1.16e-04	8230
V1, L2/3->V1, L5	same region	269	19	194	12060	19	710	-4.57	4.96e-06	3.20e-05	12327
V1, L2/3->HVA, L2/3	same region	68	11	62	4627	11	456	-3.28	1.05e-03	4.04e-03	4693
V1, L2/3->HVA, L4	same region	28	9	27	2564	9	295	-1.07	2.85e-01	4.54e-01	2590
V1, L2/3->HVA, L5	same region	68	14	65	4781	14	369	-1.45	1.47e-01	3.04e-01	4847
V1, L4->V1, L2/3	same region	12	1	12	803	1	803	1.48	1.39e-01	3.04e-01	813
V1, L4->V1, L4	same region	11	1	11	466	1	466	-1.70	9.02e-02	2.27e-01	475
V1, L4->V1, L5	same region	12	1	12	669	1	669	-3.49	5.05e-04	2.09e-03	679
V1, L5->V1, L2/3	same region	72	9	67	7716	9	899	0.71	4.77e-01	6.58e-01	7786
V1, L5->V1, L4	same region	46	10	46	4891	10	505	-2.01	4.48e-02	1.30e-01	4935
V1, L5->V1, L5	same region	119	10	110	6474	10	710	-1.45	1.47e-01	3.04e-01	6591
V1, L5->HVA, L2/3	same region	13	2	13	810	2	456	-2.35	1.92e-02	6.18e-02	821
HVA, L2/3->V1, L2/3	same region	147	27	138	23533	27	899	-3.65	2.58e-04	1.15e-03	23678
HVA, L2/3->V1, L4	same region	23	11	23	5462	11	505	0.07	9.47e-01	9.64e-01	5483
HVA, L2/3->V1, L5	same region	129	39	109	27058	39	710	0.31	7.57e-01	8.61e-01	27185
HVA, L2/3->HVA, L2/3	same region	296	46	207	19368	46	456	-7.03	2.16e-12	6.26e-11	19662
HVA, L2/3->HVA, L4	same region	93	27	77	7596	27	295	-4.58	4.63e-06	3.20e-05	7687
HVA, L2/3->HVA, L5	same region	226	44	146	14899	44	369	-4.05	5.09e-05	2.68e-04	15123
HVA, L4->V1, L5	same region	12	3	12	2066	3	710	-1.71	8.74e-02	2.27e-01	2076
HVA, L4->HVA, L2/3	same region	116	9	99	3457	9	456	-5.54	3.31e-08	6.40e-07	3571
HVA, L4->HVA, L4	same region	61	10	56	2708	10	295	-5.22	1.89e-07	2.74e-06	2767
HVA, L4->HVA, L5	same region	77	9	69	2932	9	369	-4.90	9.94e-07	1.15e-05	3007
HVA, L5->V1, L2/3	same region	72	8	71	6819	8	899	-4.59	4.42e-06	3.20e-05	6889
HVA, L5->V1, L4	same region	19	6	19	2968	6	505	-2.75	6.04e-03	2.06e-02	2985
HVA, L5->V1, L5	same region	47	14	44	9745	14	710	-1.04	2.97e-01	4.54e-01	9790
HVA, L5->HVA, L2/3	same region	159	17	136	6894	17	456	-3.93	8.66e-05	4.18e-04	7051
HVA, L5->HVA, L4	same region	60	18	50	5104	18	295	-2.91	3.69e-03	1.34e-02	5162

Continued on next page

bioRxiv preprint doi: https://doi.org/10.1101/2023.03.13.531369; this version posted March 30, 2023. The copyright holder for this preprint (which was not certified by peer review) is the author/funder, who has granted bioRxiv a license to display the preprint in perpetuity. It is made available under aCC-BY 4.0 International license.

Supplemental Table 8. Independent two-sided t-tests performed to compare receptive field center distance between connected neuron pairs and control neuron pairs across brain areas and layers
connected pair #, **connected pre #** and **connected post #** are the number of neuron pairs, the number of unique presynaptic neurons and the number of unique postsynaptic neurons included in the connected neuron pairs respectively. **control pair #**, **control pre #** and **control post #** are the number of neuron pairs, the number of unique presynaptic neurons and the number of unique postsynaptic neurons included in the control neuron pairs respectively. **t** and **p** are the test statistic and p value of the independent two-sample two-sided t tests. **adj. p** is the adjusted p value through the BH multicomparison correction procedure. **dof** is the degree of freedom of the t tests.

projection group	control	connected			control			t	p	adj. p	dof
		pair #	pre #	post #	pair #	pre #	post #				
HVA, L5->HVA, L5	same region	172	20	129	6440	20	369	-4.64	3.50e-06	3.20e-05	6610

bioRxiv preprint doi: https://doi.org/10.1101/2023.03.13.531369; this version posted March 30, 2023. The copyright holder for this preprint (which was not certified by peer review) is the author/funder, who has granted bioRxiv a license to display the preprint in perpetuity. It is made available under aCC-BY 4.0 International license.

Supplemental Table 9. Two-sided Cochran-Armitage test of trend performed to test whether connection probability increases with respect to receptive field center distances
statistic and **p** are the test statistic and p value of the two-sided Cochran-Armitage test of trend. **adj. p** is the adjusted p value through the BH multicomparison correction procedure.

projection group	control	statistic	p	adj. p
V1->V1	adp	5709	9.13e-02	3.65e-01
HVA->HVA	adp	11528	8.66e-01	8.98e-01
V1->HVA	adp	1835	8.98e-01	8.98e-01
HVA->V1	adp	3543	4.85e-01	8.98e-01
V1->V1	same region	80432	3.36e-17	6.72e-17
HVA->HVA	same region	163453	7.74e-43	3.09e-42
V1->HVA	same region	38762	4.50e-03	4.50e-03
HVA->V1	same region	210855	1.27e-07	1.70e-07

bioRxiv preprint doi: https://doi.org/10.1101/2023.03.13.531369; this version posted March 30, 2023. The copyright holder for this preprint (which was not certified by peer review) is the author/funder, who has granted bioRxiv a license to display the preprint in perpetuity. It is made available under aCC-BY 4.0 International license.

Supplemental Table 10. Independent two-sided t-tests performed to compare difference in preferred orientation between connected neuron pairs and control neuron pairs across brain areas
connected pair #, **connected pre #** and **connected post #** are the number of neuron pairs, the number of unique presynaptic neurons and the number of unique postsynaptic neurons included in the connected neuron pairs respectively. **control pair #**, **control pre #** and **control post #** are the number of neuron pairs, the number of unique presynaptic neurons and the number of unique postsynaptic neurons included in the control neuron pairs respectively. **t** and **p** are the test statistic and p value of the independent two-sample two-sided t tests. **adj. p** is the adjusted p value through the BH multicomparison correction procedure. **dof** is the degree of freedom of the t tests.

projection group	control	connected			control			t	p	adj. p	dof
		pair #	pre #	post #	pair #	pre #	post #				
V1->V1	adp	543	20	458	2864	20	1215	-0.89	3.73e-01	6.46e-01	3405
HVA->HVA	adp	719	60	480	3531	51	766	-2.14	3.25e-02	1.83e-01	4248
V1->HVA	adp	71	13	66	382	13	297	0.58	5.65e-01	6.46e-01	451
HVA->V1	adp	390	47	351	1865	44	1051	0.04	9.71e-01	9.71e-01	2253
V1->V1	same region	543	20	458	38373	20	2090	-2.00	4.59e-02	1.83e-01	38914
HVA->HVA	same region	719	60	480	57550	60	1031	-1.73	8.45e-02	2.25e-01	58267
V1->HVA	same region	71	13	66	12950	13	1031	-0.77	4.41e-01	6.46e-01	13019
HVA->V1	same region	390	47	351	95975	47	2090	0.58	5.62e-01	6.46e-01	96363

bioRxiv preprint doi: https://doi.org/10.1101/2023.03.13.531369; this version posted March 30, 2023. The copyright holder for this preprint (which was not certified by peer review) is the author/funder, who has granted bioRxiv a license to display the preprint in perpetuity. It is made available under aCC-BY 4.0 International license.

Supplemental Table 11. Independent two-sided t-tests performed to compare differences in perferred orientation between connected neuron pairs and control neuron pairs across brain areas and layers

connected pair #, **connected pre #** and **connected post #** are the number of neuron pairs, the number of unique presynaptic neurons and the number of unique postsynaptic neurons included in the connected neuron pairs respectively. **control pair #**, **control pre #** and **control post #** are the number of neuron pairs, the number of unique presynaptic neurons and the number of unique postsynaptic neurons included in the control neuron pairs respectively. **t** and **p** are the test statistic and p value of the independent two-sample two-sided t tests. **adj. p** is the adjusted p value through the BH multicomparison correction procedure. **dof** is the degree of freedom of the t tests.

projection group	control	connected			control			t	p	adj. p	dof
		pair #	pre #	post #	pair #	pre #	post #				
V1, L2/3->V1, L2/3	adp	126	9	115	634	9	347	-0.53	5.96e-01	8.45e-01	758
V1, L2/3->V1, L4	adp	50	8	50	176	8	124	0.36	7.18e-01	8.97e-01	224
V1, L2/3->V1, L5	adp	149	9	120	513	9	296	-1.32	1.87e-01	5.50e-01	660
V1, L2/3->HVA, L2/3	adp	17	4	17	83	4	73	0.49	6.25e-01	8.45e-01	98
V1, L2/3->HVA, L5	adp	23	8	21	47	8	43	0.18	8.55e-01	9.50e-01	68
V1, L4->V1, L2/3	adp	10	1	10	65	1	65	1.76	8.18e-02	4.54e-01	73
V1, L4->V1, L4	adp	17	1	17	58	1	58	-0.32	7.50e-01	9.12e-01	73
V1, L5->V1, L2/3	adp	25	7	25	110	6	97	0.08	9.37e-01	9.55e-01	133
V1, L5->V1, L4	adp	26	7	26	88	7	82	1.11	2.72e-01	5.90e-01	112
V1, L5->V1, L5	adp	79	8	74	314	8	228	-1.35	1.78e-01	5.50e-01	391
V1, L5->HVA, L2/3	adp	12	1	12	82	1	82	0.18	8.58e-01	9.50e-01	92
HVA, L2/3->V1, L2/3	adp	125	19	119	552	18	379	0.75	4.54e-01	7.32e-01	675
HVA, L2/3->V1, L4	adp	22	10	22	81	9	74	-0.16	8.74e-01	9.50e-01	101
HVA, L2/3->V1, L5	adp	109	31	98	426	30	266	-2.66	8.05e-03	2.01e-01	533
HVA, L2/3->HVA, L2/3	adp	172	28	137	661	15	262	-1.10	2.71e-01	5.90e-01	831
HVA, L2/3->HVA, L4	adp	63	19	53	139	16	98	0.70	4.88e-01	7.62e-01	200
HVA, L2/3->HVA, L5	adp	123	28	100	562	23	222	-2.06	3.96e-02	3.77e-01	683
HVA, L4->HVA, L2/3	adp	55	7	53	227	7	180	1.56	1.20e-01	5.45e-01	280
HVA, L4->HVA, L4	adp	27	7	26	67	7	57	-1.01	3.16e-01	6.09e-01	92
HVA, L4->HVA, L5	adp	34	7	33	135	7	100	-1.18	2.38e-01	5.90e-01	167
HVA, L5->V1, L2/3	adp	76	7	75	320	7	274	2.84	4.70e-03	2.01e-01	394
HVA, L5->V1, L5	adp	29	10	27	95	7	82	0.84	4.03e-01	7.06e-01	122
HVA, L5->HVA, L2/3	adp	101	14	87	424	12	242	-0.39	7.00e-01	8.97e-01	523
HVA, L5->HVA, L4	adp	23	11	21	41	10	41	-2.30	2.48e-02	3.77e-01	62
HVA, L5->HVA, L5	adp	121	15	92	410	14	201	-0.64	5.25e-01	7.96e-01	529
V1, L2/3->V1, L2/3	same region	126	9	115	7511	9	920	-1.82	6.94e-02	4.54e-01	7635
V1, L2/3->V1, L4	same region	50	8	50	3902	8	516	0.06	9.55e-01	9.55e-01	3950
V1, L2/3->V1, L5	same region	149	9	120	4927	9	621	-1.62	1.05e-01	5.25e-01	5074
V1, L2/3->HVA, L2/3	same region	17	4	17	1592	4	423	-1.50	1.34e-01	5.50e-01	1607
V1, L2/3->HVA, L5	same region	23	8	21	2682	8	344	-0.82	4.12e-01	7.06e-01	2703
V1, L4->V1, L2/3	same region	10	1	10	845	1	845	1.78	7.48e-02	4.54e-01	853
V1, L4->V1, L4	same region	17	1	17	440	1	440	-0.80	4.24e-01	7.06e-01	455
V1, L5->V1, L2/3	same region	25	7	25	6305	7	920	0.96	3.38e-01	6.26e-01	6328
V1, L5->V1, L4	same region	26	7	26	3498	7	516	0.60	5.49e-01	8.07e-01	3522
V1, L5->V1, L5	same region	79	8	74	4567	8	621	-1.38	1.69e-01	5.50e-01	4644
V1, L5->HVA, L2/3	same region	12	1	12	329	1	329	-0.11	9.10e-01	9.55e-01	339
HVA, L2/3->V1, L2/3	same region	125	19	119	16803	19	920	0.28	7.76e-01	9.12e-01	16926
HVA, L2/3->V1, L4	same region	22	10	22	5057	10	516	0.27	7.84e-01	9.12e-01	5077
HVA, L2/3->V1, L5	same region	109	31	98	18716	31	621	-1.39	1.63e-01	5.50e-01	18823
HVA, L2/3->HVA, L2/3	same region	172	28	137	10983	28	423	-1.07	2.85e-01	5.94e-01	11153
HVA, L2/3->HVA, L4	same region	63	19	53	4814	19	264	0.46	6.47e-01	8.52e-01	4875
HVA, L2/3->HVA, L5	same region	123	28	100	8947	28	344	-1.46	1.43e-01	5.50e-01	9068
HVA, L4->HVA, L2/3	same region	55	7	53	2679	7	423	1.01	3.14e-01	6.09e-01	2732
HVA, L4->HVA, L4	same region	27	7	26	1747	7	264	-1.10	2.70e-01	5.90e-01	1772
HVA, L4->HVA, L5	same region	34	7	33	2239	7	344	-1.16	2.48e-01	5.90e-01	2271
HVA, L5->V1, L2/3	same region	76	7	75	6044	7	920	2.13	3.29e-02	3.77e-01	6118
HVA, L5->V1, L5	same region	29	10	27	6086	10	621	-0.50	6.17e-01	8.45e-01	6113
HVA, L5->HVA, L2/3	same region	101	14	87	5397	14	423	0.10	9.18e-01	9.55e-01	5496
HVA, L5->HVA, L4	same region	23	11	21	2840	11	264	-2.00	4.53e-02	3.77e-01	2861
HVA, L5->HVA, L5	same region	121	15	92	4614	15	344	-1.17	2.43e-01	5.90e-01	4733

bioRxiv preprint doi: https://doi.org/10.1101/2023.03.13.531369; this version posted March 30, 2023. The copyright holder for this preprint (which was not certified by peer review) is the author/funder, who has granted bioRxiv a license to display the preprint in perpetuity. It is made available under aCC-BY 4.0 International license.

Supplemental Table 12. Two-sided Cochran-Armitage test of trend performed to test whether connection probability increases with respect to differences in preferred orientation

statistic and **p** are the test statistic and p value of the two-sided Cochran-Armitage test of trend. **adj. p** is the adjusted p value through the BH multicomparison correction procedure.

projection group	control	statistic	p	adj. p
V1->V1	adp	4370	4.10e-01	7.89e-01
HVA->HVA	adp	6405	8.32e-02	3.33e-01
V1->HVA	adp	638	7.23e-01	7.89e-01
HVA->V1	adp	3491	7.89e-01	7.89e-01
V1->V1	same region	52233	4.75e-02	1.90e-01
HVA->HVA	same region	87116	1.02e-01	2.03e-01
V1->HVA	same region	20714	3.89e-01	5.18e-01
HVA->V1	same region	145106	6.51e-01	6.51e-01
