# Supplementary material for: Functional connectomics reveals general wiring rule in mouse visual cortex
Source: bioRxiv. 2023 Mar 14:2023.03.13.531369. Preprint. [Version 1] doi: 10.1101/2023.03.13.531369 (PMC10054929; doi:10.1101/2023.03.13.531369)
Supplement: Supplementary file 1 [file NIHPP2023.03.13.531369V1-supplement-1.pdf]

bioRxiv preprint doi: https://doi.org/10.1101/2023.03.13.531369; this version posted March 14, 2023. The copyright holder for this preprint (which was not certified by peer review) is the author/funder, who has granted bioRxiv a license to display the preprint in perpetuity. It is made available under aCC-BY 4.0 International license.

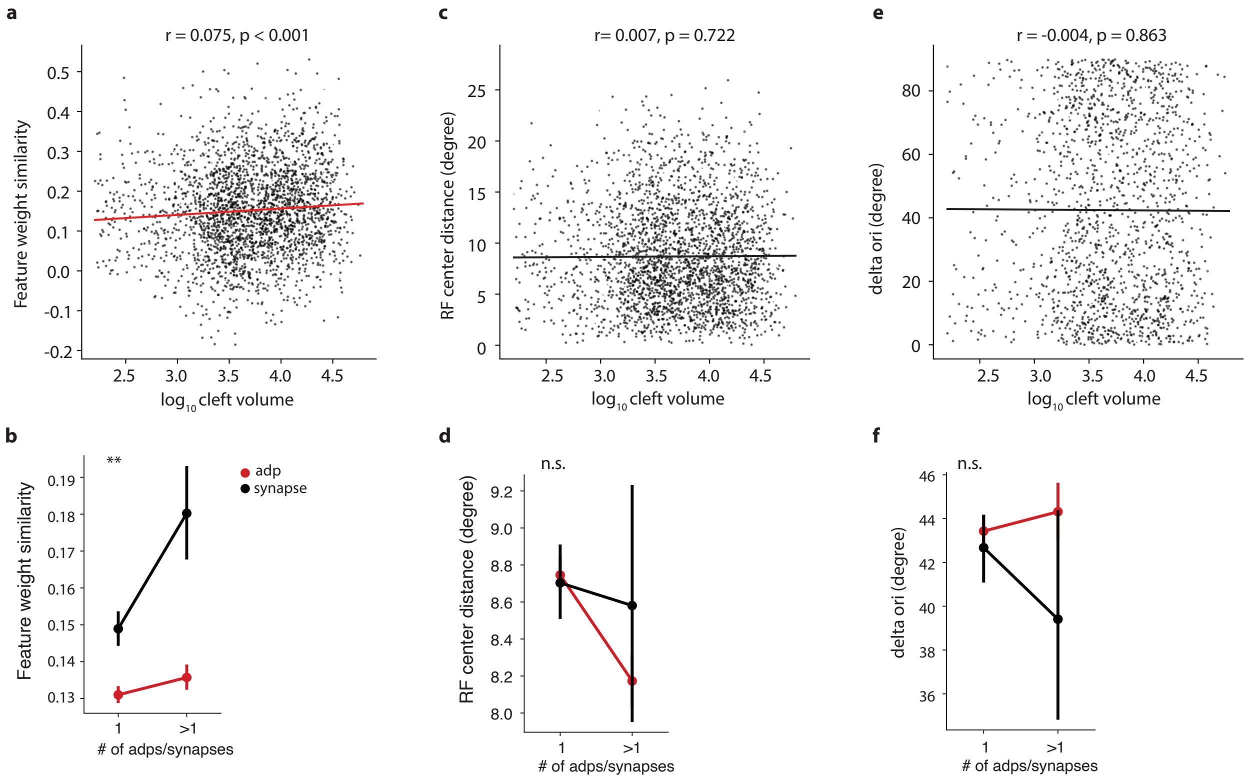

Supplemental Figure 1. Functional similarity predicts synaptic volume and number. a, c, e, Presynaptic-postsynaptic pairwise feature weight similarity (**a**), receptive field center distance (**c**), and difference in preferred orientation (**e**) as a function of synapse size (log_{10} cleft volume in voxels, r = pearson correlation coefficient, two sided p-value). **b, d, f**, Mean presynaptic-postsynaptic pairwise feature weight similarity (**b**), receptive field center distance (**d**), and difference in preferred orientation (**f**) for pairs with single versus multiple synapses (black) or ADPs (red). p-value by two way ANOVA.

bioRxiv preprint doi: https://doi.org/10.1101/2023.03.13.531369; this version posted March 14, 2023. The copyright holder for this preprint (which was not certified by peer review) is the author/funder, who has granted bioRxiv a license to display the preprint in perpetuity. It is made available under aCC-BY 4.0 International license.

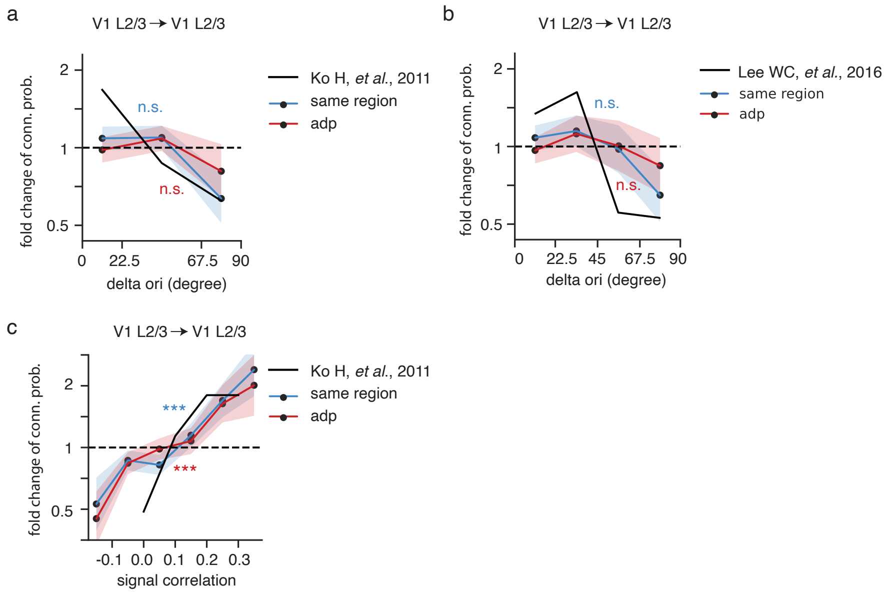

Supplemental Figure 2. Comparison of V1 L2/3 like-to-like findings to Ko et al. 2011 and Lee et al. 2016. a, As in Fig. 3i, but all presynaptic, postsynaptic, and control neurons restricted to V1 L2/3 (red,blue). Ko et al. 2011 data (black) and binning from Ko et al. 2011 Fig. 2b. **b**, As in **a**, but Lee et al. 2016 data (black) and binning from Lee et al. 2016 Fig. 2b. **c**, As in Fig. 2g, but all presynaptic, postsynaptic, and control neurons restricted to V1 L2/3 (red, blue). Ko et al. 2011 data (black) and binning from Ko et al. 2011 Fig. 3c.

bioRxiv preprint doi: https://doi.org/10.1101/2023.03.13.531369; this version posted March 14, 2023. The copyright holder for this preprint (which was not certified by peer review) is the author/funder, who has granted bioRxiv a license to display the preprint in perpetuity. It is made available under aCC-BY 4.0 International license.

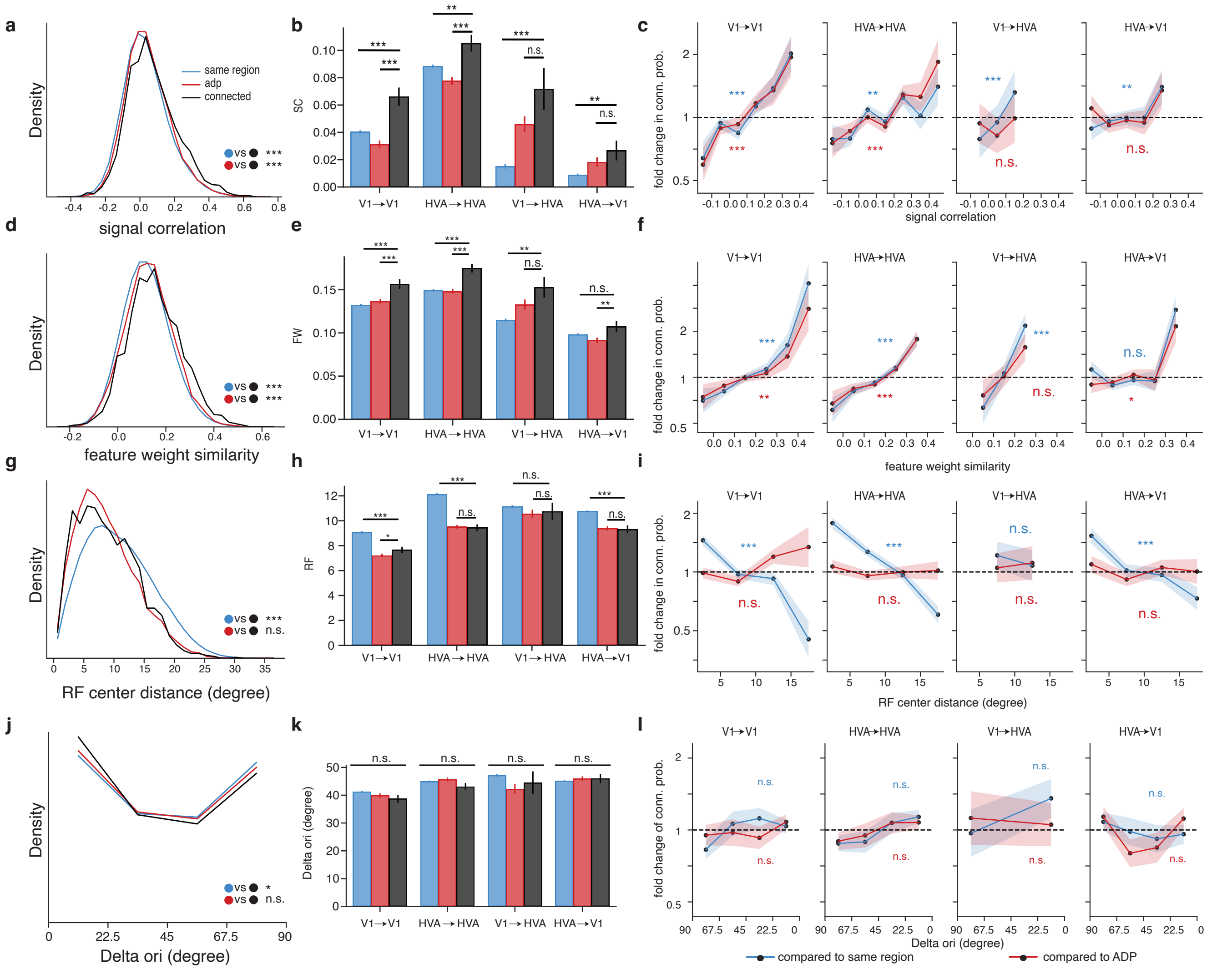

Supplemental Figure 3. Functional similarity selectivity findings consistent in orientation-tuned subsample. a, b, c, Selectivity with respect to signal correlation as Fig. 2c, e, g, but restricted to orientation-tuned neurons as in Fig. 3g, h, i. **d, e, f**, Same as **a, b, c**, but with respect to feature weight similarity as in Fig. 3a, b, c. **g, h, i**, Same as **a, b, c**, but with respect to receptive field center distance as in Fig. 3d, e, f. **j, k, l**, Same as Fig. 3g, h, i, duplicated here for reference.

bioRxiv preprint doi: https://doi.org/10.1101/2023.03.13.531369; this version posted March 14, 2023. The copyright holder for this preprint (which was not certified by peer review) is the author/funder, who has granted bioRxiv a license to display the preprint in perpetuity. It is made available under aCC-BY 4.0 International license.

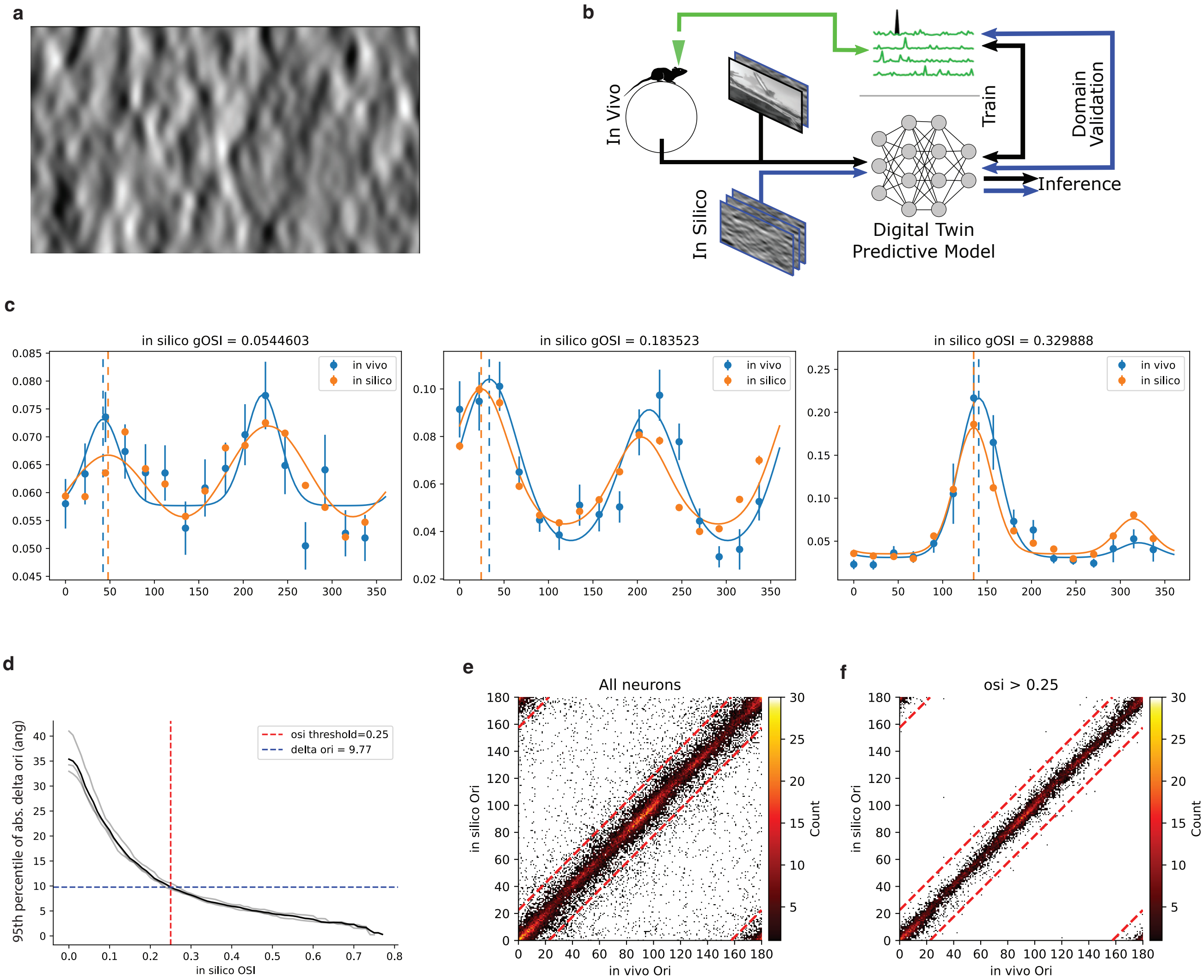

Supplemental Figure 4. In silico orientation tuning is consistent with in vivo orientation tuning a, Sample frame from global directional parametric stimulus ("Monet") used to characterize orientation and direction selectivity. Directional motion was orthogonal to orientation, and was tested at 22.5°intervals. **b**, Schematic of domain validation experimental design. In a single scan in a new animal, neuronal responses are collected in response to sufficient stimulus to both train the digital twin model and characterize orientation tuning from *in vivo* responses. Later, *in silico* orientation tuning is extracted from model responses to parametric stimuli, and compared against *in vivo* orientation tuning for the same neurons. **c**, Comparison of *in silico* and *in vivo* mean responses per stimulus direction (mean $\pm$ SEM), fitted tuning curves (lines), and extracted preferred orientation (dotted lines) for three neurons. **d**, 95th percentile difference in preferred orientation between *in silico* and *in vivo* fitted responses as a function of gOSI threshold. Dotted lines correspond to gOSI > 0.25 threshold applied for all analyses and resulting 95th percentile difference in preferred orientation $\approx 9.77\,\mathrm{deg}$ across all three animals imaged. Lines correspond to individual animals (gray) or cumulative across all animals (black). **e, f**, Two-dimensional histogram of *in silico* versus *in vivo* preferred orientation for all neurons across three animals (**e**) and only neurons with gOSI > 0.25 (**f**).
